# Self-Assembly of Functionalized Lipophilic Guanosines into Cation-Free Stacked Guanine-Quartets

**DOI:** 10.1021/acs.joc.1c00502

**Published:** 2021-07-19

**Authors:** Marilena Campitiello, Alessio Cremonini, Marco A. Squillaci, Silvia Pieraccini, Artur Ciesielski, Paolo Samorì, Stefano Masiero

**Affiliations:** † Dipartimento di Chimica “Giacomo Ciamician”, Alma Mater Studiorum−Università di Bologna, Via S. Giacomo 11, Bologna 40126, Italy; ‡ Université de Strasbourg and CNRS, ISIS, 8 allée Gaspard Monge, Strasbourg 67000, France

## Abstract

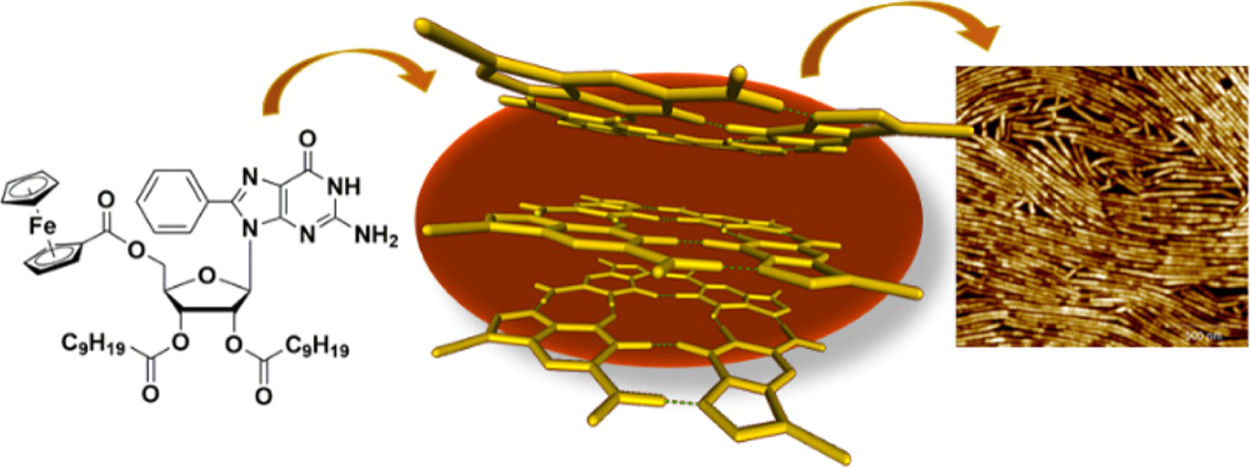

The hierarchical self-assembly of various lipophilic guanosines exposing either a phenyl or a ferrocenyl group in the C(8) position was investigated. In a solution, all the derivatives were found to self-assemble primarily into isolated guanine (G)-quartets. In spite of the apparent similar bulkiness of the two substituents, most of the derivatives form disordered structures in the solid state, whereas a specific 8-phenyl derivative self-assembles into an unprecedented, cation-free stacked G-quartet architecture.

## Introduction

Among DNA bases, guanine (G) exhibits a unique ability to undergo self-assembly, forming various distinct supramolecular structures.^1,2^ In particular, lipophilic guanosine derivatives have been reported to generate either supramolecular ribbon-like architectures^3−6^ or tetrameric array (G-quartets)-based assemblies.^7,8^ The formation of a specific assembly motif is ruled by both the specific chemical structure of the derivative^9,10^ and the environmental conditions.^10,11^ Among various G assemblies, G-quartet (hereafter *G*_4_)-based supramolecular architectures are by far the most important because of the biological relevance of this structural motif in DNA.^12,13^ Many *G*_4_-based lipophilic complexes can be found in the literature, ranging from octamers (two stacked *G*_4_s) and hexadecamers,^14−16^ these being the most frequent and studied examples, up to pseudopolymeric stacked assemblies.^17^ All these aggregates form spontaneously only in the presence of a metal cation, with the latter playing a crucial role in templating and stabilizing these architectures. For this reason, simple, isolated *G*_4_s are probably the least-described supramolecular structures that originate from guanosine because, in the absence of templating cations, the common self-assembled motif of lipophilic guanosines has a ribbon-like architecture. Isolated *G*_4_s as the main species, either in the absence^18−21^ or in the presence^22,23^ of a metal cation, have been indeed seldom reported. Isolated *G*_4_s lacking a templating ion are observed when a bulky substituent is placed at the C(8) position of the nucleobase or, more generally, in cases where the formation of G-ribbons is hindered by the molecular structure. The ability of isolated *G*_4_s, in the absence of a templating cation, to form higher-order organized assemblies has never been reported.

**Scheme 1:**
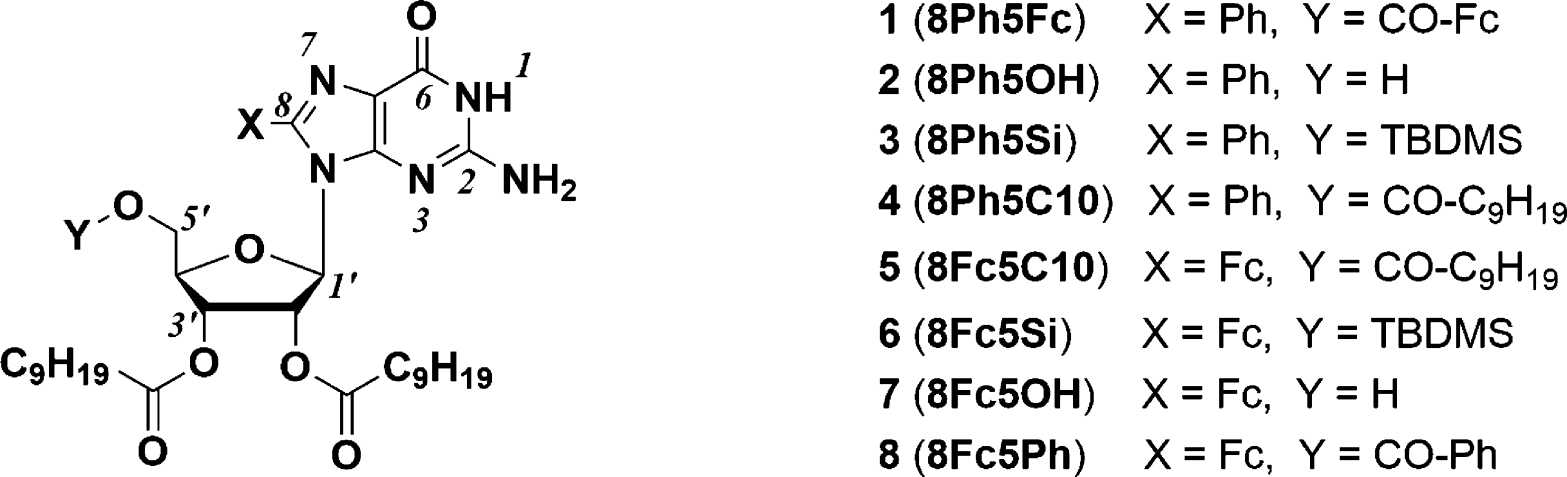
Chemical Structures of the Investigated Guanosine Derivatives

Here, we have focused our attention on both 8-ferrocenyl and 8-phenyl functionalized lipophilic guanosines carrying groups with different bulkiness at the 5′ position (Scheme 1), and we show that subtle modifications in the molecular structure cause a dramatic change in the propensity of the molecules to undergo a hierarchical self-assembly. In particular, lipophilic guanosine derivative **1** (**8Ph5Fc**) was found to form isolated *G*_4_s, which further self-assemble into stacked cation-free architectures. Conversely, derivatives **2–8**, and derivative **8** (**8Fc5Ph**) especially, where phenyl and ferrocenyl substituents are swapped with respect to **1**, exhibit no clear tendency to form supramolecular assemblies arising from piling up of *G*_4_s.

## Results and Discussion

All investigated G derivatives were synthesized, starting from commercial guanosine, as described in Schemes S1–S4.

The circular dichroism (CD) spectrum of all compounds in CH_2_Cl_2_ shows only a weak molecular induced circular dichroism (ICD) band, attributed to the chiral perturbation of the guanine chromophore by the chiral sugar residue, and does not change upon the addition of either MeOH or [2.2.2] cryptand: this rules out the presence of templating ions residual from synthetic procedures. In contrast, deliberate addition of KI produces a strong excitonic spectrum, characteristic of *G*_4_ stacking^24^ (Figure S1).

A typical feature of the ^1^H-NMR spectra of *G*_4_ structures is the splitting of the signal for exocyclic amino protons into two signals as hydrogen bonding produces a large downfield shift of the proton involved in *G*_4_ formation.^16^ Usually, this signal splitting is not observed at room temperature because the two protons are in fast exchange regime, but it can be easily monitored by lowering the temperature. Figures S2–S9 show the downfield portion of the ^1^H-NMR spectrum of the investigated compounds in CD_2_Cl_2_ at different temperatures in the absence of added metal cations. In all cases, signals that can be ascribed to amino groups start to appear at −5 to −20 °C both in the 9–10 ppm and in the 5–5.5 ppm ranges, and they become progressively sharper upon further lowering the temperature. In the case of **8Ph5Fc** (Figure S2), the amino proton signal is baseline-broadened in the CD_2_Cl_2_ room temperature spectrum. At −5 °C, two signals start to appear at 9.8 and 5.5 ppm for bound-N(2)H and free-N(2)H, respectively, and they shift slightly downfield and upfield, respectively, by further lowering the temperature. Moreover, the N(1)H imino proton signal resonates at 12.6 ppm at room temperature and undergoes deshielding upon lowering the temperature. The same signal resonates at 10.9 ppm in dmso-d_6_, suggesting that in CD_2_Cl_2_, G is H-bonded already at room temperature. All the other investigated compounds show the same behavior, yet they differ in two aspects. First, the two amino signals start to appear only at lower temperatures (compare −20 °C spectra shown in Figures S2–S9). Second, imino and amino signals of the second assembled species (e.g., 12.38 and 9.98 ppm for **8Fc5C10**, Figure S6) become visible in most cases (with the exception of the two 5’OH derivatives **8Ph5OH** and **8Fc5OH,** see below) in the downfield portion of the spectrum. No clear evidence could be obtained on the architecture of these minor species. Nonetheless, this suggests a higher stability for the aggregate formed by **8Ph5Fc**, while for derivatives **2**–**8,** other, although minor, self-assembly pathways are viable.

**Figure 1:**
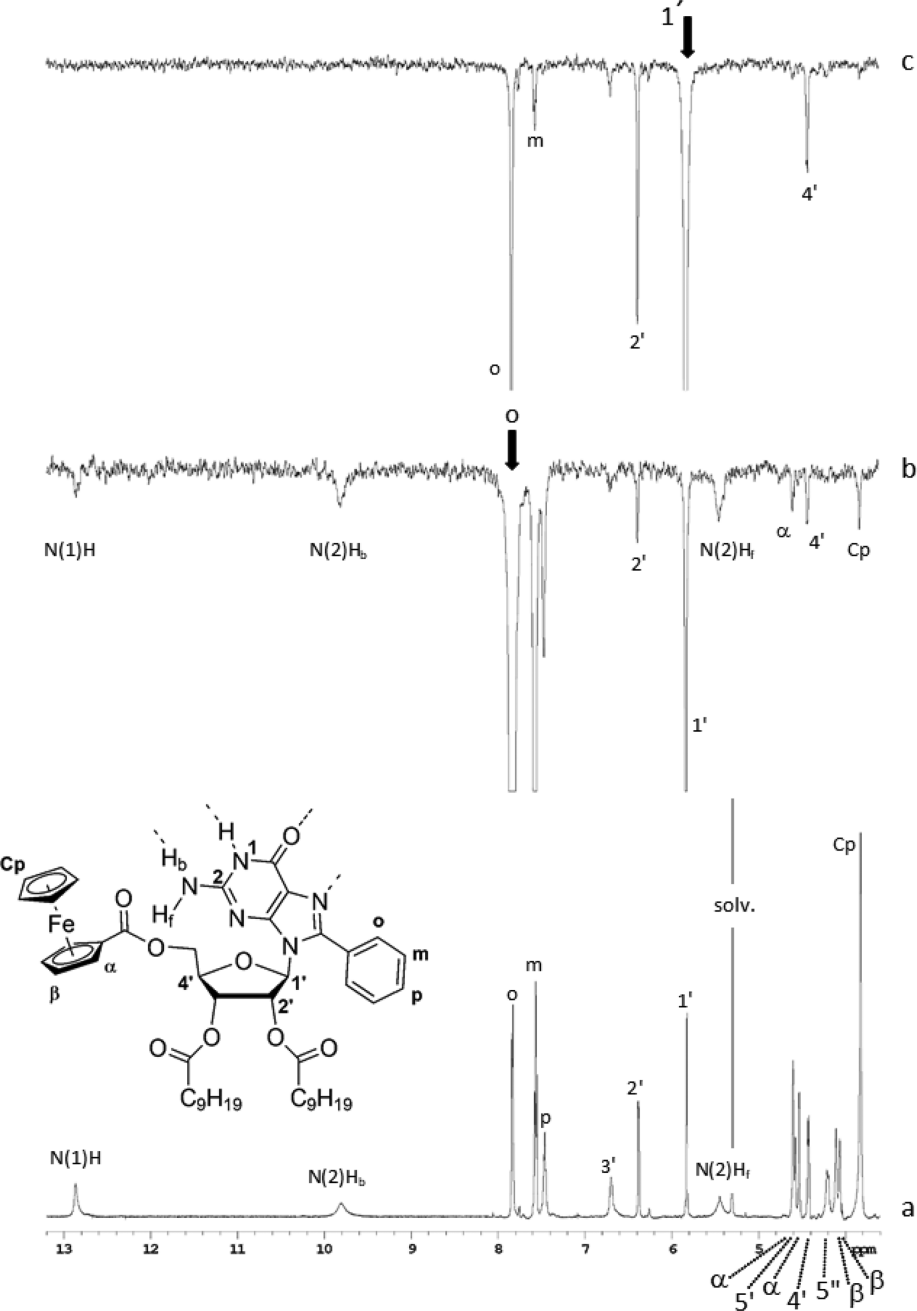
(a) Downfield portion of the 600 MHz ^1^H-NMR spectrum of **8Ph5Fc** in CD_2_Cl_2_ (4.3 mM) at −20 °C and signal assignment (diastereotopic protons were not assigned); (b) NOESY-1D spectrum of the same sample upon irradiation at 7.83 ppm (“*o*” protons); (c) NOESY-1D spectrum of the same sample upon irradiation at 5.82 ppm (1′ proton). In each NOE spectrum were used 512 coadded transients, a recycle delay of 0.6 s, a mixing time of 0.6 s, and a 50 Hz shaped pulse. Irradiated frequencies are indicated with an arrow.

Further evidence of the existence of self-assembled species in solution is given by nuclear Overhauser effect (NOE) spectra (Figures 1 and S10–S16). As the first general observation, in all cases, NOE spectra display cross-peaks having the same phase as the diagonal. This feature is characteristic of a slow tumbling regime, implying that the objects in the solution have a molecular weight above ca. 2000 Da,^25^ whereas the molecular weight of an isolated molecule of **1–8** falls in the range of 667–929 Da. Another common feature of the main species formed by all of the investigated compounds is their preference for the *syn* conformation around the glycosidic bond. Figure 1b,c shows selected one-dimensional NOE spectroscopy (1D-NOESY) spectra of **1** recorded at −20 °C as an example. The interaction between “*o*” and H1’ protons, already strong at room temperature (not shown), and the absence of any correlation between H5’/H5” and phenyl protons points to an exclusive *syn* conformation for **1**, as depicted in the inset of Figure 1a. Spectra reported in Figures S10–S16 point to the same conclusion. In the case of derivatives **8Ph5OH** and **8Fc5OH**, lacking a bulky substituent at the 5′ position, the *syn* conformer is possibly stabilized by an intramolecular H-bond between 5’OH and N(3) (see spectrum d in Figure S10).

It is known^26^ that a downfield shift of the ribose H2’ proton parallels an increase in the percentage of *syn* conformer population. In particular, 8-bromoguanosine (δ_H2’_ = 5.01 ppm in DMSO-d_6_) is ca. 90% *syn*, while guanosine (δ_H2’_ = 4.429 ppm) is only 40% *syn*. Although a direct comparison with these figures is not possible, an analysis of the observed chemical shifts for derivatives **1**–**8** (see Table S1) allows some qualitative conclusions. The introduction of ferrocenyl substituent at the 8-position exhibits a marked effect on the chemical shift of both H1’ and H2’ sugar protons, which move downfield by about 1 ppm. On the other hand, the 8-phenyl substituent exhibits a smaller effect and is limited to the H2’ and H3’ protons. Taking this result into account, it is possible to notice that the H2’ proton tends to resonate at lower fields than H1’ for the 8-phenyl derivatives, while 8-ferrocenyl derivatives show the same chemical shift order (δ_H1’_ > δ_H2’_ > δ_H3’_) as the reference compounds (entries **h**–**n** in Table S1). This suggests that the phenyl ring has a higher ability to drive the conformational equilibrium toward the *syn* isomer than the ferrocenyl substituent. In particular, the unique sequence δ_H3’_ > δ_H2’_ > δ_H1’_ shown by **8Ph5Fc** in CD_2_Cl_2_ should be noticed.

**Figure 2:**
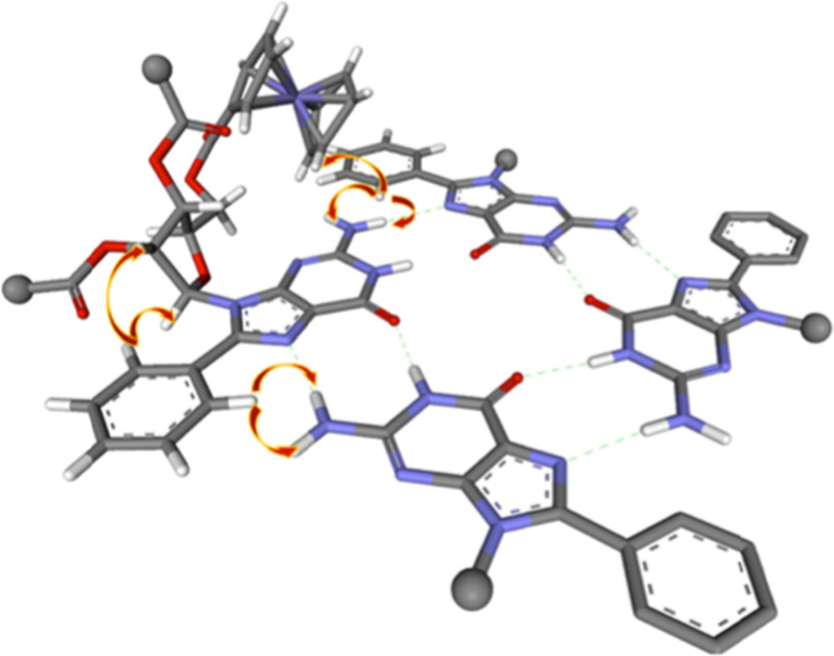
Proposed model for the isolated *G*_4_ formed by **8Ph5Fc**. Arrows indicate selected NOEs. Some atoms are omitted for clarity.

NOE spectra also provide unambiguous information on the structure of the main self-assembled species in solution. Another feature common to the main species formed by **1**–**8** is the dipolar coupling between the protons of the substituent at the C(8) position and the exocyclic amino group. For example, in the case of **8Ph5Fc**, the *o* protons interact with both exocyclic amino and ferrocene protons (Figure 1b). These proximities can only occur at the intermolecular level and rule out G-ribbon assemblies (see Figure S17) but point to a *G*_4_ structure (such as the one presented in Figure 2 in the case of **8Ph5Fc**) as the main self-assembled aggregate formed by all the derivatives.

Stabilization of this structure by residual water^27^ seems unlikely because in no case did we observe in NOE spectra a signal in the 1.5–2.5 ppm region, except for the expected exchange signals with N(1)H and N(2)H.

**Figure 3:**
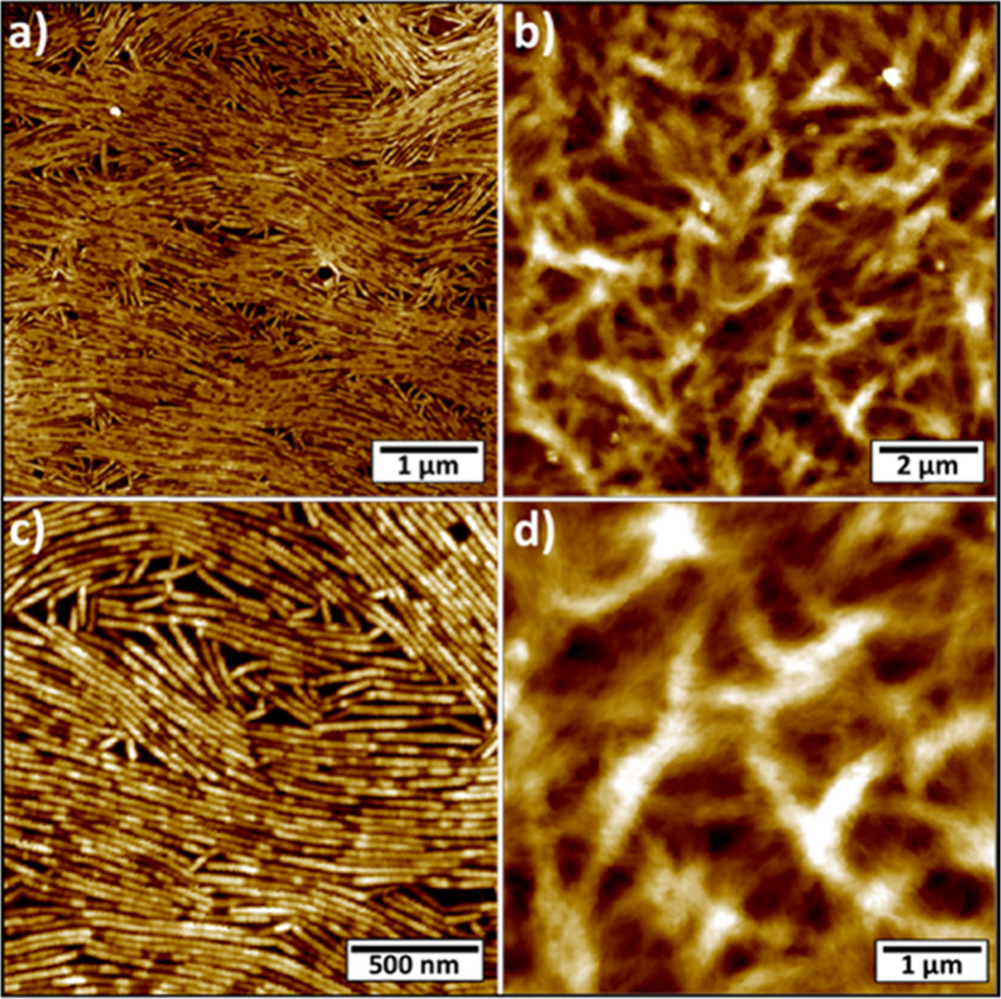
AFM images of supramolecular metal-free G aggregates drop-cast from CH_2_Cl_2_ 0.6 mg/mL on HMDS-functionalized SiO_2_. (a,c) Molecule **8Ph5Fc**. (b,d) Molecule **8Fc5C10**. Images Z-scales: (a) 10 nm; (b) 140 nm; (c) 7 nm; and (d) 110 nm.

To gain deeper insights into the propensity of the derivatives to undergo self-assembly, we have extended our study to atomic force microscopy (AFM) imaging. In addition to **8Ph5Fc**, derivatives **8Fc5C10** and **8Ph5C10** were studied for comparison. The samples were prepared by drop-casting 150 μL of guanosine solutions in CH_2_Cl_2_ (concentration 0.6 mg/mL) onto the basal plane of thermally grown SiO_2_/Si (230 nm SiO_2_ on Si [100]). Prior use, the substrates were cleaned and functionalized with a monolayer of hexamethyldisilazane (HMDS), covalently bonded to the pending SiOH groups, in order to render the surface hydrophobic and to avoid the formation of hydrogen bonds between the molecules and the substrate. Figure 3 displays the topographical AFM images of films of **8Ph5Fc** (Figure 3a,c) and **8Fc5C10** (Figure 3b,d) prepared using the same experimental conditions.

The observed morphologies are markedly different. Molecule **8Fc5C10** forms a disordered network of entangled bundles of fibers, while **8Ph5Fc** self-assembles into layers of ordered and well-aligned fibers possessing an average height of 4.1 ± 0.5 nm (measured with respect to the widest empty areas) and an average width of 32 ± 4 nm. Being the lateral size of the fibers comparable to the curvature radius of the used AFM tips (nominal radius 7 nm), the measured width value must be corrected to exclude the broadening artifact given by the mechanical convolution effect. Considering the measured width and an interfiber penetration of the tip of ∼1.8 nm, we can apply well-established models^28^ to conclude that the real width of such fibers amounts to 19 ± 4 nm. As the radius of the G-quartet structure in columnar aggregates, both in water^29^ and in organic solvents,^17^ amounts to 12.5 Å and a fully elongated decanoyl residue measures ca.12 Å, it is possible to estimate a diameter of 4.8 nm for the *G*_4_*s* formed by **8Ph5Fc**. This value is in good agreement with the average measured height for the fibers. Assuming a 3.3 Å spacing (characteristic of optimal nucleobase stacking^30^), the cylindrical fibers shown in Figure 3a,c are composed on an average of six stacked *G*_4_s.

**Figure 4:**
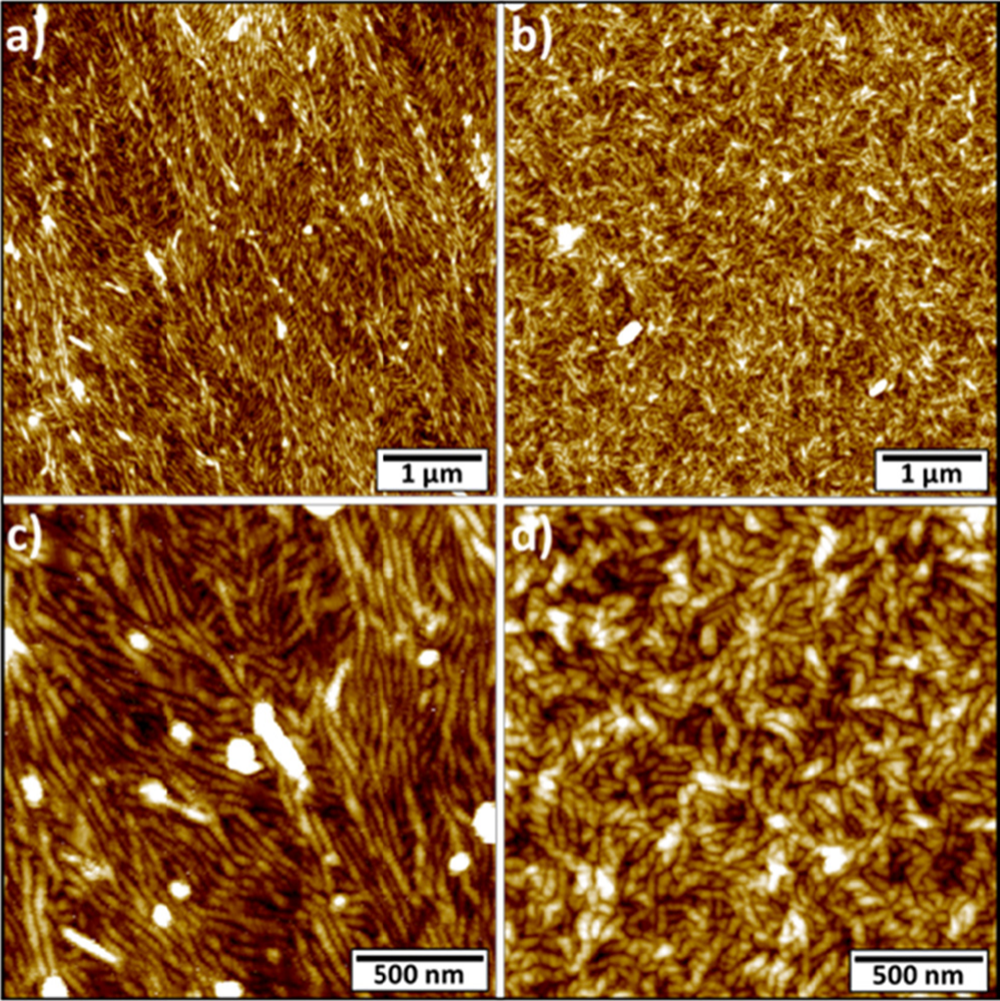
AFM images of supramolecular metal-free G aggregates drop-cast from CH_2_Cl_2_ 0.6 mg/mL on HMDS-functionalized SiO_2_ after SVA (CH_2_Cl_2_ for 48 h). (a,c) Compound **8Ph5Fc**. (b,d) Compound **8Fc5C10**. Images Z-scales: (a) 13 nm; (b) 25 nm; (c) 12 nm; and (d) 20 nm.

To promote the aggregation into more stable and ordered supramolecular structures, the films were then exposed to solvent vapor annealing (SVA) in CH_2_Cl_2_ for 48 h under ambient conditions. Such a method makes it possible to trigger the molecular reorganization toward the generation of thermodynamically favored architectures. It is noteworthy that this method has previously been successfully used to finely tune the self-assembly of various molecular systems, including *n*-type perylene nanowires,^31^ p-type pentacene,^32^ hexa-*peri*-hexabenzocoronene (HBC)^33^ structures, and porphyrins.^34^Figure 4 portrays the AFM images of the **8Ph5Fc** (a,c) and **8Fc5C10** (b,d) fibers formed on the surface after the SVA treatment.

Interestingly, the two compounds showed a similar behavior: in both cases, SVA caused an increased entanglement of the fibrillar structures, resulting in an extremely uniform coverage of the underneath substrate, which is no longer exposed. In particular, in the case of **8Ph5Fc**, the as-deposited fibers show high rigidity and no tendency to entangle, causing the formation of holes and discontinuities in the resulting films, as shown in Figure 3a,c. After the SVA treatment, the fibers assemble into less-ordered bundles, forming a multilayered uniform film (Figure 4a,c). In the latter case, the fibers feature an average measured width of 41 ± 3 nm, suggesting that the packing at the molecular level is not affected by the SVA treatment, but it is not possible to correct the data with the previously used model because of the multilayer nature of the film after SVA. In the case of compound **8Fc5C10**, the SVA process exhibits a strong impact over the morphology of the films that undergo a dramatic decrease in roughness from 23.2 nm (before SVA, Figures 3b,d) to 4.15 nm (after SVA, Figures 4b,d). Moreover, the compound **8Fc5C10** fibers show completely different conformations: before SVA, the fibrillar structure is hardly visible, while after that, the fibrillar units are extremely well defined and form a strongly entangled network.

On the other hand, samples of **8Ph5C10** showed no tendency to form crystalline structures. In this case, AFM characterization has not revealed any well-defined structures, and the films look amorphous (see Figure S18).

## Conclusions

In summary, we have designed and synthesized several organic soluble guanosines carrying either a phenyl or a ferrocenyl group at the C(8) position. Their self-assembly has been studied in solution by NMR and in thin films by means of AFM. Derivative **8Ph5Fc** shows the exclusive existence of isolated *G*_4_s in the CD_2_Cl_2_ solution, while compounds **8Fc5C10** and **8Ph5C10** do form isolated *G*_4_s as well, but another aggregated species is present in equilibrium in solution. The hierarchical self-assembly of **8Ph5Fc** and **8Fc5C10** leads to the formation of fibers, which can be deposited and characterized on surfaces. Guanosine **8Fc5C10** shows the formation of a disordered film, arising from the coexistence of different self-assembled species (probably a mixture of stacked *G*_4_s and H-bonded dimeric/oligomeric ribbon-like aggregates), whereas **8Ph5Fc** cleanly assembles into unprecedented, stacked cation-free *G*_4_ architectures. The ferrocenyl moiety of **8Fc5C10** seems to be not large enough to drive the self-assembly toward the exclusive existence of isolated *G*_4_s in solution, while its shape seems to hamper π-stacking and hence a shift in equilibria toward (stacked) quartets in the solid state. On the other hand, the results obtained for **8Ph5C10** indicate that the self-assembly is not ruled uniquely by the nature of the C(8) substituent: the behavior observed only for **8Ph5Fc** suggests that also the nature of the 5′ substituent, although irrelevant for *G*_4_ formation, affects the subsequent self-assembly. These results further expand the range of supramolecular architectures that can be obtained in thin films and provide unambiguous evidence for the key role played by the subtle design of guanosine starting building blocks to control the hierarchical self-assembly motifs that can be attained.

## Experimental Procedures

### General Methods

All reactions that require anhydrous conditions were carried out under a dry argon atmosphere in oven-dried glassware. Macherey-Nagel polygram silica gel plates (layer thickness 0.20 mm) were used for thin layer chromatography (TLC) analyses. Column chromatography was performed on Geduran silica gel 60 (40–63 μm). Reagents and solvents, including dry solvents, were purchased from Sigma-Aldrich or TCI. Electrospray ionization (ESI) mass spectra were obtained from methanol solutions in either the positive or negative mode, with Micromass ZMD 4000 or ZQ-4000 instruments. High-resolution mass spectrometry (HRMS) spectra were recorded on a Waters Xevo G2-XS QTof system. Nuclear magnetic resonance (NMR) spectra were recorded on Varian Inova (600, 400, or 300 MHz) spectrometers and referenced to the residual solvent resonance (s = singlet, bs = broad singlet, d = doublet, t = triplet, q = quartet, qi = quintet, m = multiplet; coupling constant are in Hz). Structural assignments were made with additional information from gCOSY (gradient-selected correlation spectroscopy), gHSQC (gradient-selected heteronuclear single-quantum correlation), and gHMBC (gradient-selected heteronuclear multiple bond correlation) experiments. Diastereotopic protons/carbons were not assigned. CD spectra were recorded on a Jasco J-710 spectropolarimeter. Synthetic schemes with compound numbering are reported in the Supporting Information (S1–S4)

### Microscopy Studies

The AFM study of the self-assembled **1**, **4**, and **5** was performed using Veeco/Bruker Dimension 3100 that runs with a Nanoscope IV controller. Solutions of investigated molecules were prepared in 0.6 mg/mL CH_2_Cl_2_ and deposited by drop-casting (150 μL) on clean SiO_2_/Si (230 ± 10 nm SiO_2_ on [100] Si) functionalized with a monolayer of HMDS. The SVA treatment was performed by placing the compound films in a sealed environment and saturating with CH_2_Cl_2_ vapor under ambient conditions, for 48 h. The raw AFM data were processed through the application of background flattening.

#### 8-Bromo Guanosine **9**

The compound was prepared according to a literature procedure.^35^ Thus, commercial guanosine **5** (1.00 g, 3.53 mmol) was suspended in 60 mL of an acetonitrile/water 2:1 mixture, and *N*-bromosuccinimide (943 mg, 5.3 mmol, 1.5 eq) was added in three portions over 20 min. Stirring was continued until TLC (CH_2_Cl_2_/MeOH 8:2) revealed the disappearance of the starting material (2 h). Solvents were then removed in vacuo, and the pale-yellow solid thus obtained was suspended in acetone (20 mL) and stirred at room temperature for 2 h. The flask was then placed in a refrigerator and left overnight at −20 °C. The precipitate was filtered and washed several times with cold acetone to afford 1.09 g, 3.02 mmol (yield 86%) of 8-bromo guanosine **9** as a white solid.

ESI-MS (*m*/*z*): 362.1/363.9 [M + H]^+^; 383.9/386.0 [M + Na]^+^; 360.1/362.0 [M – H]^−^.

HRMS (ESI/Q-TOF) *m*/*z*: [M + Na]^+^ calcd for C_10_H_12_BrN_5_O_5_Na 383.9914; found 383.9919.

^1^H-NMR (dmso-d_6_, 600 MHz): δ 10.81 (bs, 1H, NH), 6.50 (bs, 2H, NH_2_), 5.68 (d, 1H, *J* = 6.0 Hz, H1’), 5.44 (d, 1H, *J* = 6.0 Hz, OH2’), 5.08 (d, 1H, *J* = 5.1 Hz, OH3’), 5.01 (m, 1H, H2’), 4.92 (m, 1H, OH5’), 4.13 (m, 1H, H3’), 3.85 (m, 1H, H4’), 3.63 (m, 1H, H5’), 3.54 (m, 1H, H5’). ^13^C{^1^H} NMR (dmso-d_6_, 75 MHz): δ 155.9, 153.9, 152.5, 121.5, 118.0, 90.1, 86.3, 70.9, 70.7, 62.5

#### 8-Bromo-5’-O-*tert*-butyldimethylsilyl Guanosine **10**

8-Bromo guanosine **9** (1.274 g, 3.52 mmol) was dried at 50 °C over P_2_O_5_ in vacuo for 2 h and dissolved in dimethylformaimde (DMF) (17 mL). Imidazole (491 mg, 7.21 mmol, 2.05 eq) was added. To the resulting mixture was then added dropwise a solution of *t*-butyldimethylsilyl chloride (558 mg, 3.70 mmol, 1.05 eq.) in tetrahydrofuran (THF) (9 mL), and stirring was continued for 2 h. As TLC (CH_2_Cl_2_/MeOH 8:2) revealed the presence of unreacted starting material, an extra amount of imidazole (120 mg, 1.76 mmol, 0.5 eq.) and *t*-butyldimethylsilyl chloride (266 mg, 1.76 mmol, 0.5 eq.) was added, and reaction was continued for 2 h. The crude mixture was poured into water (50 mL), and the precipitate was filtered, washed with water and Et_2_O, and dried to afford the product (1.04 g, 2.19 mmol, 62%) as a white solid.

RF = 0.6 (CH_2_Cl_2_/MeOH 8:2).

ESI-MS (*m*/*z*): 473.9/475.9 [M – H]^−^; 475.9/477.9 [M + H]^+^.

HRMS (ESI/Q-TOF) *m*/*z*: [M + H]^+^ calcd for C_16_H_27_BrN_5_O_5_Si 476.0959; found 476.0965.

^1^H-NMR (CD_3_OD, 600 MHz): δ 5.85 (d, 1H, *J* = 4.2 Hz, H1’), 5.26 (dd, 1H, *J* = 5.7 Hz, *J* = 4.2 Hz, H2’), 4.56 (m, 1H, *J* = 5.7 Hz, H3’), 3.95 (m, 1H, *J* = 11.4 Hz, *J* = 5.7 Hz, *J* = 4.0 Hz, H4’), 3.92 (1H, dd, *J* = 11.4 Hz, *J* = 4.0 Hz, H5’), 3.83 (dd, 1H, *J* = 11.4 Hz, *J* = 5.7 Hz, H5’), 0.84 (s, 9H, *t*BuSi), 0.01 and −0.03 (s,s, 6H, SiMe_2_). ^13^C{^1^H} NMR (CD_3_OD, 151 MHz): δ

#### 8-Phenyl-5’-O-*tert*-butyldimethylsilyl Guanosine **11**

PdCl_2_(PPh_3_)_2_ (59.0 mg, 0.084 mmol, 0.2 eq.) was added to a degassed solution of **10** (200 mg, 0.421 mmol), phenylboronic acid (77 mg, 0.630 mmol, 1.5 eq.), and K_3_PO_4_ (223 mg, 1.05 mmol) in 7 mL of a 6:1 mixture of dioxane/water. The mixture was heated at 95 °C for 22 h in an oil bath. After cooling to room temperature, solvents were removed by distillation, the dark residue was suspended in 50 mL of Et_2_O and filtered. The filtrate was washed with brine (40 mL) and 1 N HCl (6 mL) and then dried over MgSO_4_. The precipitate was washed with a CH_2_Cl_2_/MeOH 4:1 mixture; washings were combined with the ethereal residue, and solvents were removed in vacuo. The residue was purified by column chromatography on silica gel (CH_2_Cl_2_/MeOH 95:5), and the product was obtained in a 61% yield (121 mg, 0.256 mmol) as a white solid.

RF = 0.2 (CH_2_Cl_2_/MeOH 9:1).

ESI-MS (*m*/*z*): 472.4 [M – H]^−^; 474.1 [M + H]^+^; 496.2 [M + Na]^+^.

HRMS (ESI/Q-TOF) *m*/*z*: [M + H]^+^ calcd for C_22_H_32_N_5_O_5_Si 474.2167; found 474.2173.

^1^H-NMR (dmso-d_6_, 600 MHz): δ 10.73 (bs, 1H, NH), 7.67–7.64 (m, 2H, ArH), 7.54–7.52 (m, 3H, ArH), 6.43 (bs, 2H, NH_2_), 5.60 (d, 1H, *J* = 5.5 Hz, H1’), 5.36 (d, 1H, *J* = 6.0 Hz, OH), 5.07 (m, 1H), 4.94 (d, 1H, *J* = 6.0 Hz, OH), 4.13 (m, 1H), 3.84–3.74 (m, 3H, H4’,H5’, H5″), 0.841 (s, 9H, *t*BuSi), 0.006 and 0.003 (s,s, 6H, SiMe_2_). ^13^C{^1^H} NMR (dmso-d_6_, 75 MHz): δ 157.2 (C), 153.5 (C), 152.7 (C), 147.8 (C), 130.7 (C), 129.9 (CH, Ph), 129.5 (CH, Ph), 129.1 (CH, Ph), 117.4 (C), 89.7 (C1’), 85.3 (C4’), 70.7 (C3’), 70.5 (C2’), 64.1 (C5’), 26.3 (CH_3_), 18.5 (C), −4.8 (CH_3_), −4.8 (CH_3_).

#### 8-Phenyl-5’-O-*tert*-butyldimethylsilyl-2′,3’-O-didecanoyl Guanosine **3** (8Ph5Si)

8-Phenyl-5’-O-*tert*-butyldimethylsilyl guanosine **11** (100 mg, 0.211 mmol) was dried in vacuo over P_2_O_5_ at 50 °C for 1 h and dissolved in 15 mL of an acetonitrile/toluene 2:1 mixture. Decanoic anhydride (163 μL, 0.444 mmol, 2.10 eq.), Et_3_N (64 μL, 0.444 mmol, 2.10 eq.), and a small amount of 4-dimethylaminopyridine (DMAP) were then added, and the mixture was heated at 80 °C in an oil bath for 7 h. After cooling to room temperature, MeOH (0.5 mL) was added, and stirring was continued for 20 min. Solvents were removed by distillation, the residue was dissolved in CH_2_Cl_2_, washed with 5% NaHCO_3_ and brine, and dried over MgSO_4_. After the removal of solvents, the residue was purified by chromatography on silica gel (CH_2_Cl_2_/MeOH 99:1), and the product (120 mg, 0.153 mmol) was obtained as a white solid in 72% yield.

RF = 0.36 (CH_2_Cl_2_/MeOH 97:3).

ESI-MS (*m*/*z*): 780.6 [M – H]^−^; 782.6 [M + H]^+^; 804.6 [M + Na]^+^.

HRMS (ESI/Q-TOF) *m*/*z*: [M + Na]^+^ calcd for C_42_H_67_N_5_O_7_SiNa 804.4702; found 804.4713.

^1^H-NMR (CD_2_Cl_2_, 600 MHz): δ 12.38 (bs, 1H, NH), 7.77 (d, 2H, *orto*-ArH), 7.54–7.59 (m, 3H, ArH), 6.37 (m, 1H, H2’), 6.17 (m, 1H, H3’), 5.77 (d, 1H, *J* = 3.2 Hz, H1’), 4.14 (m, 1H, H4’), 3.90 (1H, dd, *J* = 12.0 Hz, 4.2 Hz, H5’), 3.78 (dd, 1H, *J* = 12.0 Hz, 5.0 Hz, H5’), 2.28 (t, 2H, −CH_2_–CO), 2.24 (t, 2H, −CH_2_–CO), 1.60 (m, 2H, CH_2_–CH_2_–CO), 1.52 (m, 2H, CH_2_–CH_2_–CO), 1.28–1.23 (m, 24H, CH_2_), 0.87 and 0.85 (t,t, 6H, CH_3_), 0.78 (s, 9H, *t*BuSi), 0.07 and 0.04 (s,s, 6H, SiMe_2_). ^13^C{^1^H} NMR (CD_2_Cl_2_, 151 MHz): δ 172.4 (C=O), 172.3 (C=O), 159.4 (C), 153.6 (C), 152.4 (C4), 148.6 (C8), 130.0 (CH, Ph), 129.5 (C), 129.3 (CH, Ph), 129.1 (CH, Ph), 116.9 (C), 87.5 (C1’), 82.0 (C4’), 72.1 (C2’), 70.3 (C3’), 62.6 (C5’), 34.0 (CH_2_), 33.9 (CH_2_), 32.01 (CH_2_), 31.97 (CH_2_), 29.6 (CH_2_), 29.5 (CH_2_), 29.5 (CH_2_), 29.4 (CH_2_), 29.4 (CH_2_), 29.2 (CH_2_), 29.1 (CH_2_), 25.6 (CH_3_), 24.9 (CH_3_), 24.9 (CH_2_), 22.8 (CH_2_), 22.8 (CH_2_), 18.2 (C), 14.0 (CH_3_), 14.0 (CH_3_), −5.6 (CH_3_), −5.7 (CH_3_).

#### 8-Phenyl-2′,3’-O-didecanoyl Guanosine **2** (8Ph5OH)

To a solution of **3** (120 mg, 0.153 mmol) in THF (4 mL) were added 72.5 mg (0.230 mmol, 1.5 eq) of tetra-*n*-butylammonium fluoride (TBAF). The mixture was stirred at room temperature until TLC (CH_2_Cl_2_/MeOH 92:8) showed complete disappearance of the starting material. The reaction mixture was concentrated, redissolved in CH_2_Cl_2_, and washed with sat. NaHCO_3_ (3 × 10 mL). The organic phase was dried over MgSO_4_, and solvents were removed by distillation. The residue was purified on silica gel by first eluting with Et_2_O and then with CH_2_Cl_2_/MeOH 95:5. The product was obtained as a white solid in a 73% yield (75 mg, 0.112 mmol).

RF = 0.28 (CH_2_Cl_2_/MeOH 92:8).

ESI-MS (*m/z*): 666.6 [M – H]^−^; 668.4 [M + H]^+^; 690.4 [M + Na]^+^.

HRMS (ESI/Q-TOF) m/z: [M + Na]^+^ calcd for C_36_H_53_N_5_O_7_Na 690.3837; found 690.3846.

^1^H-NMR (dmso-d_6_, 600 MHz): δ 10.84 (bs, 1H, NH), 7.61–7.58 (m, 2H, *o*-ArH), 7.55–7.53 (m, 3H, *m*- and *p*-ArH), 6.48 (bs, 2H, NH_2_), 6.18 (t, 1H, OH), 5.78 (d, 1H, *J* = 6.0 Hz, 1’H), 5.49 (m, 1H, 2’H), 5.18 (m, 1H, 3’H), 4.08 (m, 1H, 4’H), 3.75 (m, 1H, 5’H), 3.64 (m, 1H, 5’H), 2.35–2.16 (m, 4H, CH_2_–CO), 1.48 and 1.40 (qi, qi, 4H, CH_2_–CH_2_–CO), 1.27–1.12 (m, 24H, CH_2_), 0.85 and 0.84 (t,t, 6H, CH_3_). ^13^C{^1^H} NMR (dmso-d_6_, 151 MHz): δ 172.3 (C=O 3′), 171.8 (C=O 2′), 159.6 (C), 153.7 (C), 153.5 (C), 147.4 (C8), 131.9 (CH, *p*-Ph), 130.5 (C, Ph), 129.9 (CH, *o*-Ph), 129.2 (CH, *m*-Ph), 119.6 (C), 88.1 (C1’), 85.1 (C4’), 72.1 (C3’), 71.7 (C2’), 61.6 (C5’), 33.9 (CH_2_), 33.6 (CH_2_), 31.8 (CH_2_), 29.3 (CH_2_), 29.3 (CH_2_), 29.3 (CH_2_), 29.2 (CH_2_), 29.2 (CH_2_), 29.2 (CH_2_), 29.0 (CH_2_), 28.9 (CH_2_), 24.8 (CH_2_), 24.5 (CH_2_), 22.6 (CH_2_), 14.0 (CH_3_).

#### 8-Phenyl-5’-O-ferrocenoyl-2′,3′-O-didecanoyl Guanosine **1** (8Ph5Fc)

Ferrocene carboxylic acid (77.5 mg, 0.337 mmol, 1.3 eq.) was dried in vacuo at 50 °C for 1 h and dissolved in THF (9 mL). Et_3_N (175 μL, 1.217 mmol, 4.7 eq.) was added, and the resulting solution was cooled to 0 °C. Methanesulfonyl chloride (25.5 μL, 0.311 mmol, 1.2 eq.) was added, and the mixture was allowed to warm to room temperature and stirred for 2 h. A solution of vacuum-dried 8-phenyl-2′,3′-O-didecanoyl guanosine **2** (173 mg, 0.259 mmol) in THF (6 mL) was then added, followed by a catalytic amount of DMAP. Stirring was continued for 24 h, MeOH (0.5 mL) was added, and the reaction mixture was concentrated in vacuo. The residue was dissolved in CH_2_Cl_2_, washed with water, and dried over MgSO_4_. The solvent was removed by distillation, and the residue was purified by chromatography on silica gel (gradient from CH_2_Cl_2_ to CH_2_Cl_2_/MeOH 99:1), affording the product (85 mg, 0.097 mmol) as a yellow solid in 38% yield.

RF = 0.24 (CH_2_Cl_2_/MeOH 9:1).

ESI-MS (*m/z*): 878.6 [M – H]^−^; 880.5 [M + H]^+^; 902.5 [M + Na]^+^.

HRMS (ESI/Q-TOF) *m*/*z*: [M + Na]^+^ calcd for C_47_H_61_FeN_5_O_8_Na 902.3762; found 902.3776.

^1^H-NMR (CD_2_Cl_2_, 600 MHz): δ 12.57 (bs, 1H, NH), 7.81 (d, 2H, *J* = 7.1 Hz, Ph), 7.56 (t, 2H, *J* = 7.1 Hz, Ph), 7.49 (t, 1H, *J* = 7.1 Hz, Ph), 6.48 (dd, 1H, *J* = 7.8 Hz, 5.4 Hz, H3′), 6.43 (dd, 1H, *J* = 5.4 Hz, 3.0 Hz, H2’), 5.85 (d, 1H, *J* = 3.0 Hz, H1’), 4.67 (s, 1H, Fc), 4.62 (dd, 1H, *J* = 12.0 Hz, 4.3 Hz, H5’), 4.61 (s, 1H, Fc), 4.41 (m, 1H, H4’), 4.34 (dd, 1H, *J* = 12.0 Hz, 4.3 Hz, H5’), 4.20 (s, 1H, Fc), 4.17 (s, 1H, Fc), 3.97 (s, 5H, Fc), 2.36–2.26 (m, 4H, CH_2_–CO), 1.67–1.52 (m, 4H, CH_2_–CH_2_–CO), 1.33–1.20 (m, 24H, CH_2_), 0.88–0.84 (m, 6H, CH_3_). ^13^C{^1^H} NMR (CD_2_Cl_2_, 151 MHz): δ 172.5 (C, CO(3’)), ’)), 117.1 (C), 87.6 (CH, C1’), 79.9 (CH, C4’), 72.5 (CH, C2’), 71.8 (CH, Fc), 71.7 (CH, Fc), 70.1 (C, Fc), 70.1 (CH, Fc), 70.0 (CH, C3’), 69.8 (CH, Fc), 61.7 (CH_2_, C5’), 34.0 (CH_2_), 33.9 (CH_2_), 32.0 (CH_2_), 32.0 (CH_2_), 29.8 (CH_2_), 29.6 (CH_2_), 29.5 (CH_2_), 29.5 (CH_2_), 29.4 (CH_2_), 29.4 (CH_2_), 29.2 (CH_2_), 29.1 (CH_2_), 25.7 (CH_2_), 24.9 (CH_2_), 22.8 (CH_2_), 14.0 (CH_3_)

#### 8-Phenylguanosine **12**

To a degassed mixture of **9** (273 mg, 0.754 mmol), phenylboronic acid (110 mg, 0.902 mmol), Na_2_CO_3_ (160 mg, 1.51 mmol), tris(3-sulfonatophenyl)phosphine (TPPTS) (11 mg, 0.0194 mmol) in H_2_O (2.4 mL), and acetonitrile (1.2 mL) were added 44 mg (0.0196 mmol) of Pd(OAc)_2_, and the reaction mixture was stirred at 83 °C in an oil bath overnight. TLC (RP18, H_2_0/CH_3_OH 1:1) confirmed the disappearance of the starting material. The reaction mixture was then cooled to room temperature and diluted with water (20 mL). The pH was adjusted to 7 with 1 N HCl. The resulting suspension was refluxed for a short time, cooled to room temperature, and maintained at 4 °C for 2 h. The precipitate was filtered, washed with water, and air-dried to afford the product as a white solid in 80% yield (216 mg, 0.60 mmol). The compound was sufficiently pure and was used in the following step without further purification.

ESI-MS (*m*/*z*): 360.2 [M – H]^+^.

HRMS (ESI/Q-TOF) *m*/*z*: [M + H]^+^ calcd for C_16_H_18_N_5_O_5_, 360.1302; found 360.1308.

^1^H-NMR (dmso-d_6_, 400 MHz): δ 10.75 (s, 1H, NH), 7.68–7.65 (m, 2H, Ph), 7.54–7.52 (m, 3H, Ph), 6.38 (bs, 2H, NH_2_), 5.64 (d, 1H, *J* = 6.4 Hz, H1’), 5.36 (d, 1H, *J* = 6.4 Hz, OH_2′_), 5.05–5.00 (m, 2H, H2′, OH_5′_), 4.96 (d, 1H, *J* = 5.6 Hz, OH_3′_), 4.09–4.06 (m, 1H, H3’), 3.85–3.81 (m, 1H, H4’), 3.70–3.65 (m, 1H, H5’), 3.58–3.52 (m, 1H, H5’).^13^C{^1^H} NMR (dmso-d_6_, 101 MHz): δ 157.0 (C), 153.5 (C), 152.4 (C), 147.9 (C), 130.5 (C, Ph), 129.8 (CH, Ph), 129.6 (CH, Ph), 129.0 (CH, Ph), 117.6 (C), 89.4 (CH), 86.3 (CH), 71.0 (CH), 70.7 (CH), 62.5 (CH_2_).

#### 8-Phenyl-2′,3′,5′-O-tridecanoyl Guanosine **4** (**8Ph5C10**)

To a stirred suspension of **12** (97 mg, 0.27 mmol) in MeCN (4 mL) were added Et_3_N (0.19 mL, 1.35 mmol), decanoic anhydride (0.33 mL, 0.89 mmol), and a catalytic amount of DMAP. The mixture was stirred at room temperature and monitored by TLC (DCM/MeOH 8:2). After 2 h, the mixture was concentrated under reduced pressure, and the resulting oil was partitioned between DCM and water. The organic layer was collected, the solvent was removed by distillation, and the residue was purified by column chromatography on silica (DCM/acetone 9:2.5 to remove decanoic acid and then DCM/MeOH 9:1) to afford triester **4** as a white waxy solid in 59% yield (131 mg, 0.16 mmol).

ESI-MS (*m/z*): 822.6 [M + H]^+^, 844.6 [M + Na]^+^.

HRMS (ESI/Q-TOF) *m*/*z*: [M + Na]^+^ calcd for C_46_H_71_N_5_O_8_Na 844.5195; found 844.5200.

^1^H-NMR (CD_2_Cl_2_, 600 MHz): δ 12.43 (bs, 1H, NH), 7.74 (m, 2H, *o*-Ph), 7.58 (m, 2H, *m*-Ph), 7.53 (m, 1H, *p*-Ph), 6.29 (dd, 1H, *J* = 5.6 Hz, 3.8 Hz, H2’), 6.16 (t, 1H, *J* = 5.9 Hz, H3’), 5.86 (d, 1H, *J* = 3.7 Hz, H1’), 4.52 (m, 1H, H5’), 4.30 (m, 1H, H4’), 4.26 (m, 1H, H5’), 2.33–2.21 (m, 6H, CH_2_–CH_2_–CO), 1.64–1.59 (m, 2H, CH_2_–CH_2_–CO), 1.53–1.49 (m, 4H, 2 CH_2_–CH_2_–CO), 1.31–1.18 (m, 36H, CH_2_), 0.87 (t, 3H, CH_3_), 0.85 (t, 3H, CH_3_), 0.81 (t, 3H, CH_3_).^13^C{^1^H} NMR (CD_2_Cl_2_, 151 MHz): δ 173.4 (CO^5’^) 172.4 (CO), 172.2 (CO), 159.2 (C), 153.7 (C), 152.2 (C4), 148.3 (C8), 129.9 (Ph), 129.4 (Ph), 129.3 (Ph), 129.1 (Ph), 117.1 (C5), 87.7 (CH, C1’), 79.4 (CH, C4’), 72.3 (CH, C2’), 70.6 (CH, C3’), 62.8 (CH_2_, C5’), 34.0 (CH_2_), 34.0 (CH_2_), 33.9 (CH_2_), 32.0 (CH_2_), 32.0 (CH_2_), 31.9 (CH_2_), 29.6 (CH_2_), 29.5 (CH_2_), 29.5 (CH_2_), 29.4 (CH_2_), 29.4 (CH_2_), 29.4 (CH_2_), 29.3 (CH_2_), 29.2 (CH_2_), 29.1 (CH_2_), 24.9 (CH_2_), 24.9 (CH_2_), 24.9 (CH_2_), 22.8 (CH_2_), 22.8 (CH_2_), 22.7 (CH_2_), 14.0 (CH_2_), 13.9 (CH_3_).

#### 8-Ferrocenylguanosine **13**

A mixture of **9** (0.300 g, 0.83 mmol), dimethoxyethane (DME) (12 mL), ferroceneboronic acid (0.286 g, 1.24 mmol, 1.5 eq), and NaOH (3 M, 5.25 mL, 15.75 mmol) was degassed with a stream of Ar for 40 min in an ultrasonic bath. PdCl_2_(PPh_3_)_2_ (0.058 g, 0.1 eq) was added, and the resulting solution was refluxed at 85 °C under argon in an oil bath for 48 h. The solvent was then removed under reduced pressure, the reaction mixture was neutralized with 10% HCl, and the solid was filtered, washed with water, and dried. The product thus obtained was used in the subsequent step without further purification.

An analytical sample was obtained by chromatography on silica (DCM/MeOH 8:2).

RF = 0.17 (DCM/MeOH 9:1).

ESI-MS (*m/z*): 466.3 [M – H]; 468.1 [M + H]^+^; 490.1 [M + Na]^+^.

HRMS (ESI/Q-TOF) *m*/*z*: [M + Na]^+^ calcd for C_20_H_21_FeN_5_O_5_Na 490.0784; found 490.0790.

^1^H-NMR (dmso-d_6_, 600 MHz): δ 10.67 (bs, 1H, NH), 6.65 (d, 1H, *J* = 7.2 Hz, H1’), 6.3 (bs, 2H, NH_2_), 5.53 (d, 1H, *J* = 6.0 Hz, OH^2’^), 5.36 (d, 1H, *J* = 6.0 Hz, OH^3’^), 5.24 (m, 1H, OH^5’^), 5.16 (m, 1H, H2’), 4.68 (s, 2H, Fc), 4.62 (s, 2H, Fc), 4.31 (s, 5H, Fc), 4.19 (m, 1H, H3’), 3.97 (m, 1H, H4’), 3.71 and 3.57 (m, m, 2H, H5’,H5).”^13^C{^1^H} NMR (dmso-d_6_, 151 MHz): δ 157.8, 153.3, 152.4, 146.9, 117.7, 86.3, 80.4, 75.2, 71.8, 70.8, 70.2, 70.1, 69.9, 69.6, 68.9, 62.8.

#### 8-Ferrocenyl-2′,3′,5’-O-tridecanoyl Guanosine **5** (**8Fc5C10**)

Crude 8-ferrocenylguanosine **13** (0.430 g, 1.0 eq, 0.92 mmol) was dried over P_2_O_5_ in vacuo for 2 h at 55 °C and then suspended in 30 mL of an acetonitrile–toluene 1:1 mixture. Decanoic anhydride (443 μL, 3.15 eq, 2.90 mmol) and triethylamine (209 μL, 3.15 eq, 2.90 mmol) were then added, followed by a catalytic amount of 4-dimethylamino pyridine. The mixture was stirred under argon for 14 h at 80 °C in an oil bath. A second aliquot of decanoic anhydride (443 μL, 3.15 eq, 2.90 mmol) and triethylamine (TEA) (209 μL, 3.15 eq, 2.90 mmol) was added, and stirring was continued for 12 h at the same temperature. The solvent was removed under reduced pressure, and the crude was dissolved in dichloromethane and extracted with a sat. NaHCO_3_ and brine. The organic layer was dried over MgSO_4_. The crude reaction mixture was then applied to a silica gel column packed in DCM and eluted with a mixture of DCM–methanol (from 99:1 to 95:5). The product was obtained as an orange glass in 23% yield (196 mg, 0.21 mmol).

ESI-MS (*m/z*): 930.5 [M + H]^+^; 952.4 [M + Na]^+^.

HRMS (ESI/Q-TOF) *m*/*z*: [M + H]^+^ calcd for C_50_H_76_FeN_5_O_8_ 930.5038; found 930.5042.

^1^H-NMR (dmso-d_6_, 600 MHz): δ 10.77 (bs, 1H, NH), 6.75 (d, 1H, *J* = 6.0 Hz, H1’), 6.58 (t, 1H, *J* = 6.0 Hz, H2’), 6.40 (bs, 2H, NH_2_), 5.74 (dd, 1H, *J* = 6.0 Hz, 4.2 Hz, H3’), 4.64 (s, 1H, Fc), 4.60 (s, 1H, Fc), 4.50 (bs, 2H, Fc), 4.48 (m, 1H, H5’), 4.37 (m, 1H, H4’), 4.30 (m, 1H, H5’), 4.28 (s, 5H, Fc), 2.45–2.27 (m, 6H, CH_2_–CH_2_–CO), 1.58 (qi, 2H, *J* = 6.6 Hz, CH_2_–CH_2_–CO), 1.47 (m, 4H, 2 CH_2_–CH_2_–CO), 1.24–1.17 (m, 36H, CH_2_), 0.85–0.82 (m, 9H, CH_3_). ^13^C{^1^H} NMR (dmso-d_6_, 151 MHz): δ 175.0 (CO), 173.1 (CO^5’^), 172.2 (CO^2’^), 156.6 (C), 153.6 (C), 152.6 (C4), 146.1 (C8), 117.3 (C5), 86.6 (CH, C1’), 80.1 (CH, C4’), 74.5 (C, Fc), 71.1 (CH, C3’), 70.6 (CH, C2’), 70.2 (CH, Fc), 70.0 (CH, Fc), 69.8 (CH, Fc), 68.7 (CH, Fc), 68.6 (CH, Fc), 63.0 (CH_2_, C5’), 34.1 (CH_2_), 33.9 (CH_2_), 33.8 (CH_2_), 33.6 (CH_2_), 31.7 (CH_2_), 29.3 (CH_2_), 29.3 (CH_2_), 29.2 (CH_2_), 29.2 (CH_2_), 29.1 (CH_2_), 29.1 (CH_2_), 29.0 (CH_2_), 29.0 (CH_2_), 28.8 (CH_2_), 28.8 (CH_2_), 25.0 (CH_2_), 24.9 (CH_2_), 24.8 (CH_2_), 24.7 (CH_2_), 22.5 (CH_2_), 14.4 (CH_3_), 14.4 (CH_3_).

#### 8-Ferrocenyl-5’-O-*tert*-butyldimethylsilyl Guanosine **14**

A mixture of **13** (0.135 g, 0.29 mmol), imidazole (39 mg, 0.58 mmol), and *t*-butyldimethylsilyl chloride (48 mg, 0.32 mmol) in DMF (3 mL) was stirred at room temperature for 2 h. After the completion of the reaction (TLC DCM/MeOH 8:2), water (10 mL) was added, and the resulting precipitate was filtered and washed with water. The dry residue was purified by column chromatography (DCM/MeOH 92:8) to afford the product as a white solid in 62% yield (104 mg, 0.18 mmol).

RF = 0.6 (CH_2_Cl_2_/MeOH 8:2).

ESI-MS (*m/z*): 580.4 [M – H]^−^; 582.1 [M + H]^+^; 604.2 [M + Na]^+^.

HRMS (ESI/Q-TOF) *m*/*z*: [M + H]^+^ calcd for C_26_H_36_FeN_5_O_5_Si 582.1830; found 582.1835.

^1^H-NMR (dmso-d_6_, 400 MHz): δ 10.64 (s, 1H, NH), 6.51 (d, 1H, *J* = 5.6 Hz, H1’), 6.34 (bs, 2H, NH_2_), 5.50 (d, 1H, *J* = 5.6 Hz, OH_2’_), 5.27–5.23 (m, 1H, H2’), 5.07 (d, 1H, *J* = 5.6 Hz, OH_3’_), 4.67–4.66 (m, 2H, Fc), 4.47–4.45 (m, 2H, Fc), 4.29 (s, 5H, Fc), 4.27 (m, 1H, H3’), 3.92–3.85 (m, 2H, H5’, H4’), 3.79–3.75 (m, 1H, H5’), 0.82 (s, 9H, *t*BuSi), −0.014 and −0.010 (s,s, 6H, SiMe_2_). ^13^C{^1^H} NMR (dmso-d_6_, 101 MHz): δ 156.7, 153.1, 152.8, 146.7, 117.4, 89.3, 85.1, 75.2, 71.0, 70.4, 69.9, 69.9, 69.7, 69.2, 68.8, 64.2, 26.2, 18.4, −4.7, −4.8.

#### 8-Ferrocenyl-5’-O-*tert*-butyldimethylsilyl-2′,3’-O-didecanoyl Guanosine **6** (8Fc5Si)

To a suspension of **14** (71 mg, 0.12 mmol) in MeCN (4 mL) were added Et_3_N (45 μL, 0.32 mmol), decanoic anhydride (100 μL, 0.27 mmol), and a catalytic amount of DMAP. The mixture was stirred at room temperature for 4 h. MeOH (0.2 mL) was then added, and solvents were removed under reduced pressure. The residue was partitioned between DCM (20 mL) and water (10 mL). The organic layer was further washed with water (10 mL) and concentrated in vacuo to give an orange glassy solid, which was purified by column chromatography on silica (gradient from pure DCM to DCM/MeOH 97:3). Pure DCM (300 mL ca.) was used to elute decanoic acid, and then, DCM/MeOH 97:3 was used to elute two orange bands: the first one consisting of pure **6** (55 mg, 0.06 mmol, yield 50%), while the second one containing the desilylated derivative **7** formed during work-up.

ESI-MS (*m/z*): 888.7 [M – H]^−^; 890.5 [M + H]^+^, 912.6 [M + Na]^+^.

HRMS (ESI/Q-TOF) *m*/*z*: [M + H]^+^ calcd for C_46_H_72_FeN_5_O_7_Si 890.4545; found 890.4533.

^1^H-NMR (CD_2_Cl_2_, 600 MHz): δ 12.33 (bs, 1H, NH), 6.82 (d, 1H, *J* = 4.7 Hz, H1’), 6.74 (m, 1H, H2’), 6.18 (m,1H, H3’), 4.82 (bs, 2H, Fc), 4.53 (bs, 1H, Fc), 4.48 (bs, 1H, Fc), 4.27 (s, 5H, Fc), 4.28–4.24 (m, 1H, H4’), 3.94 (dd, 1H, *J* = 11.4 Hz, 4.7 Hz, H5’), 3.83 (dd, 1H, *J* = 11.4 Hz, 4.7 Hz, H5’), 2.38 (m, 2H, CH_2_–CH_2_–CO), 2.33 (t, 2H, CH_2_–CH_2_–CO), 1.70–1.64 (m, 2H, CH_2_–CH_2_–CO), 1.61–1.56 (m, 2H, CH_2_–CH_2_–CO), 1.36–1.23 (m, 24H, CH_2_), 0.87 (t, 3H, CH_3_), 0.84 (t, 3H, CH_3_), 0.82 (s, 9H, t-Bu), 0.00 (s, 3H, SiMe), −0.030 (s, 3H, SiMe). ^13^C{^1^H} NMR (CD_2_Cl_2_, 151 MHz): δ 172.5 (CO), 172.2 (CO), 158.8 (C), 153.2 (C), 152.8 (C4), 148.2 (C8), 116.9 (C), 86.4 (CH, C1’), 82.4 (CH, C4’), 73.8 (C, Fc), 71.4 (CH, C2’), 70.7 (CH, C3’), 70.5 (CH, Fc), 70.2 (CH, Fc), 69.8 (CH, Fc), 69.0 (CH, Fc), 68.3 (CH, Fc), 62.6 (CH_2_, C5’), 34.2 (CH_2_), 34.0 (CH_2_), 32.0 (CH_2_), 31.9 (CH_2_), 29.6 (CH_2_), 29.5 (CH_2_), 29.4 (CH_2_), 29.4 (CH_2_), 29.3 (CH_2_), 29.2 (CH_2_), 25.6 (CH_3_), 25.1 (CH_2_), 24.9 (CH_2_), 22.8 (CH_2_), 22.7 (CH_2_), 18.2 (C), 14.0 (CH_3_), −5.6 (SiMe).

#### 8-Ferrocenyl-2′,3’-O-didecanoyl Guanosine **7** (**8Fc5OH**)

To a solution of **6** (55 mg, 0.06 mmol) in 3 mL of THF were added 30 mg (0.09 mmol) of TBAF^.^3H_2_O, and the mixture was stirred at room temperature for 20 h. The solvent was removed by evaporation, and the residue was dissolved in DCM (15 mL) and washed sequentially with 5% NaHCO_3_ (5 mL) and water (5 mL, 4 times). The organic fraction was then dried over Na_2_SO_4_, and the solvent was removed by distillation. The residue was purified by chromatography on silica gel (gradient from pure DCM to DCM/MeOH 96:4) to afford **7** (33 mg, 0.04 mmol) as yellowish glass in 69% yield.

RF = 0.32 (DCM/MeOH 95:5).

ESI-MS (*m/z*): 774.5 [M – H]^−^; 776.3 [M + H]^+^, 798.3 [M + Na]^+^.

HRMS (ESI/Q-TOF) *m*/*z*: [M + Na]^+^ calcd for C_40_H_57_FeN_5_O_7_Na 798.3500; found 798.3506.

^1^H-NMR (CD_2_Cl_2_, 600 MHz): δ 12.31 (bs, 1H, NH), 7.22 (d, 1H, *J* = 13.8 Hz, H1’), 6.98 (bs, 2H, NH_2_), 6.50 (bs, 1H, OH), 6.12 (dd, 1H, *J* = 13.8 Hz, 7.2 Hz, H2’), 5.71 (d, 1H, *J* = 7.2 Hz, H3’), 4.78 (bs, 1H, Fc), 4.57 (bs, 2H, Fc), 4.52 (bs, 1H, Fc), 4.35 (s, 5H, Fc), 4.37–4.31 (m, 1H, H4’), 4.01–3.96 (m, 1H, H5’), 3.87–3.80 (m, 1H, H5’), 2.47 (t, 2H, CH_2_–CO), 2.28 (t, 2H, CH_2_–CO), 1.72 (m, 2H, CH_2_–CH_2_–CO), 1.54 (m, 2H, CH_2_–CH_2_–CO), 1.40–1.20 (m, 24H, CH_2_), 0.88 (t, 3H, CH_3_), 0.82 (t, 3H, CH_3_). ^13^C{^1^H} NMR (CD_2_Cl_2_, 151 MHz): δ 172.5 (CO), 171.9 (CO), 158.5 (C), 153.5 (C), 151.7 (C), 148.1 (C), 117.5 (C), 86.2 (CH, C1’), 85.7 (CH, C4’), 73.6 (C, Fc), 72.9 (CH, C3’), 71.6 (CH, C2’), 70.9 (CH, Fc), 70.2 (CH, Fc), 69.7 (CH, Fc), 69.5 (CH, Fc), 68.8 (CH, Fc), 63.1 (CH_2_, C5’), 34.5 (CH_2_), 33.8 (CH_2_), 32.0 (CH_2_), 31.9 (CH_2_), 29.6 (CH_2_), 29.5 (CH_2_), 29.4 (CH_2_), 29.3 (CH_2_), 29.1 (CH_2_), 25.2 (CH_2_), 24.7 (CH_2_), 22.8 (CH_2_), 14.0 (CH_3_).

#### 8-Ferrocenyl-5’-O-benzoyl-2′,3’-O-didecanoyl Guanosine **8** (**8Fc5Ph**)

Guanosine derivative **7** (54 mg, 0.07 mmol) was dried in vacuo over P_2_O_5_ at 50 °C for 2 h and then dissolved in DMF (1 mL). To the resulting solution were added Et_3_N (15 μL, 0.10 mmol), benzoic anhydride (21 mg, 0.09 mmol), and a catalytic amount of DMAP. The mixture was stirred at room temperature for 8 h, and then, water (10 mL) and DCM (10 mL) were added. The organic phase was washed with water (10 mL), and the combined aqueous fractions were washed with DCM (2 × 5 mL). The organic fractions were combined, and solvents were removed under reduced pressure. The residue was purified by chromatography on silica (DCM/MeOH 96:4) to afford derivative **8** (52 mg, 0.06 mmol) as a yellow–orange solid in 85% yield.

RF = 0.13 (DCM/MeOH 96:4).

ESI-MS (*m/z*): 878.5 [M – H]^−^; 880.4 [M + H]^+^, 902.4 [M + Na]^+^.

HRMS (ESI/Q-TOF) *m*/*z*: [M + H]^+^ calcd for C_47_H_62_FeN_5_O_8_ 880.3942; found 880.3922.

^1^H-NMR (CD_2_Cl_2_, 600 MHz): δ 12.40 (bs, 1H, NH), 7.92 (bd, 2H, ortho-Ph), 7.40 (bt, 1H, para-Ph), 7.31 (bt, 2H, meta-Ph), 6.90 (d, 1H, *J* = 3.8 Hz, H1’), 6.63 (dd, 1H, *J* = 5.7 Hz, 3.8 Hz, H2’), 5.51 (t, 1H, *J* = 5.7 Hz, H3’), 4.83–4.79 (m, 3H, H5’, 2Fc), 4.58–4.54 (m, 2H, H4’, H5’), 4.51 (bs, 1H, Fc), 4.48 (bs, 1H, Fc), 4.25 (s, 5H, Fc), 2.41–2.37 (m, 4H, 2 CH_2_–CO), 1.69 (m, 2H, CH_2_–CH_2_–CO), 1.62 (m, 2H, CH_2_–CH_2_–CO), 1.39–1.21 (m, 24H, CH_2_), 0.88 (t, 3H, CH_3_), 0.85 (t, 3H, CH_3_). ^13^C{^1^H} NMR (dmso-d_6_, 151 MHz): δ 172.2 (CO), 172.2 (CO), 165.8 (CO), 158.5 (C), 153.5 (C), 152.4 (C), 146.1 (C), 133.9 (CH), 129.7 (C), 129.6 (CH), 128.1 (CH), 117.2 (C), 92.2 (CH), 86.7 (CH), 79.7 (C), 74.4 (CH), 71.0 (CH), 70.8 (CH), 69.9 (CH), 69.8 (CH), 68.7 (CH), 68.6 (CH), 63.5 (CH_2_), 33.9 (CH_2_), 33.6 (CH_2_), 31.7 (CH_2_), 31.7 (CH_2_), 29.3 (CH_2_), 29.2 (CH_2_), 29.2 (CH_2_), 29.1 (CH_2_), 29.1 (CH_2_), 28.9 (CH_2_), 28.8 (CH_2_), 24.8 (CH_2_), 24.7 (CH_2_), 22.5 (CH_2_), 14.3 (CH_3_).

## Supporting Information Available

Synthetic schemes, VT ^1^H-NMR spectra, 1D-NOESY spectra, figures and tables, NMR spectra of compounds **1**–**14** (PDF)
