# Supplementary material for: Self-Assembly of Functionalized Lipophilic Guanosines into Cation-Free Stacked Guanine-Quartets
Source: J Org Chem. 2021 Jul 19;86(15):9970–8. doi: 10.1021/acs.joc.1c00502 (PMC8389894; doi:10.1021/acs.joc.1c00502)
Supplement: Supplementary file 1 — jo1c00502_si_001.pdf [file jo1c00502_si_001.pdf]

Self-assembly of functionalized lipophilic guanosines into cation-free stacked G-quartets

Marilena Campitiello,[a] Alessio Cremonini,[a] Marco A. Squillaci,[b] Silvia Pieraccini,[a] Artur Ciesielski,[b] Paolo Samorì,*[b] Stefano Masiero *[a]

[a] Alma Mater Studiorum - Università di Bologna, Dipartimento di Chimica "Giacomo Ciamician", Via S. Giacomo 11, 40126 Bologna, Italy. E-mail: stefano.masiero@unibo.it

[b] Université de Strasbourg, CNRS, ISIS, 8 allée Gaspard Monge, 67000 Strasbourg, France.

Synthetic schemes 1-4	S2
Selected CD spectra (Figure S1)	S4
VT ^{1}H-NMR of compounds **1-8** (Figure S2-S9)	S5
Table S1	S13
Selected 1D-NOESY spectra of compounds **1-8** (Figure S10-S16)	S14
Figure S17	S21
Figure S18	S22
Spectra of compounds **1-14**	**S**23

Synthetic schemes

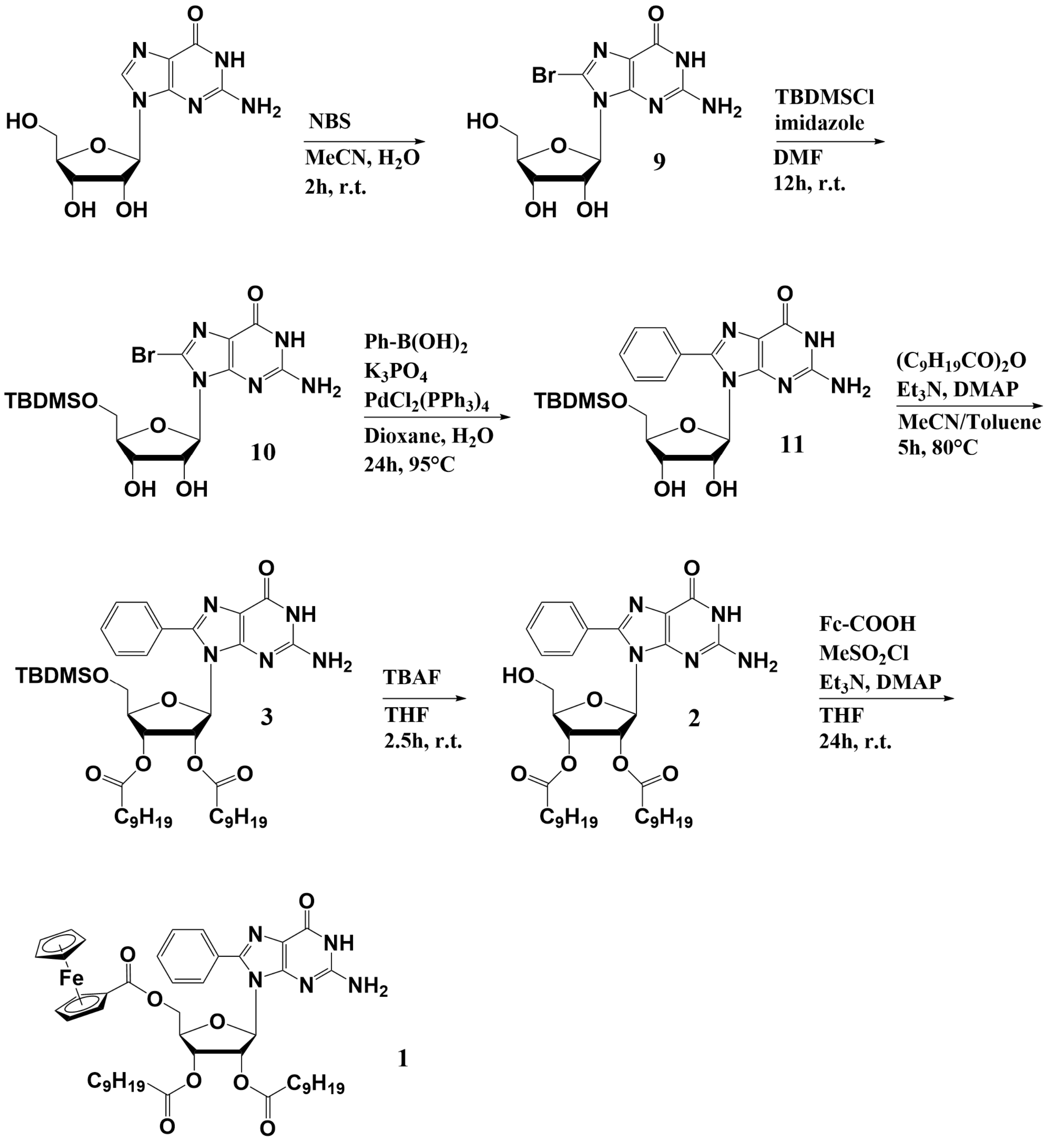

Scheme S1. Synthesis of guanosines **1-3** from commercial guanosine.

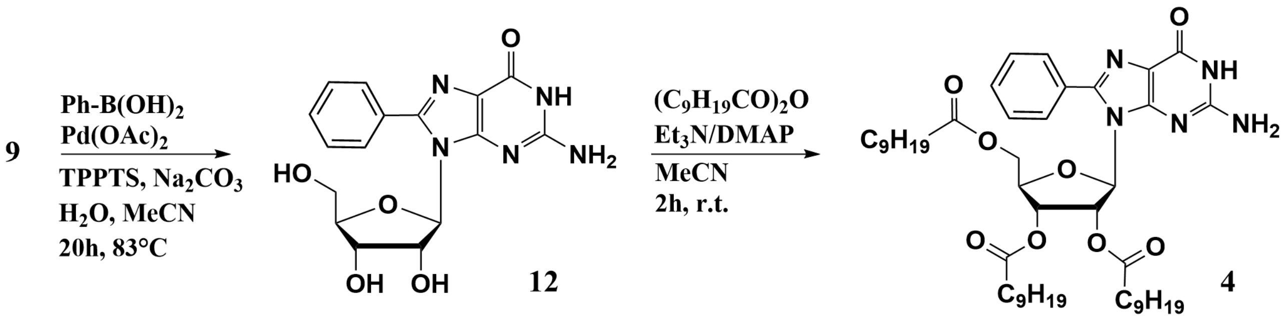

Scheme S2. Synthesis of guanosine **4** (**8Ph5C10**).

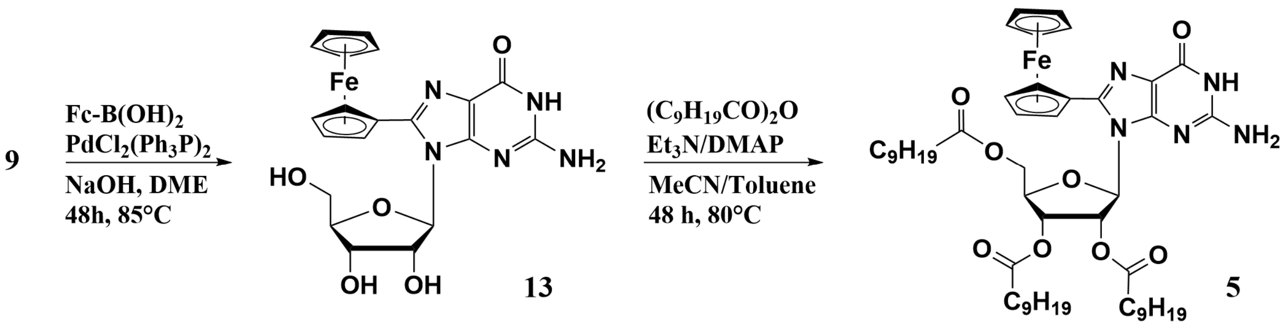

Scheme S3. Synthesis of guanosine **5** (**8Fc5C10**).

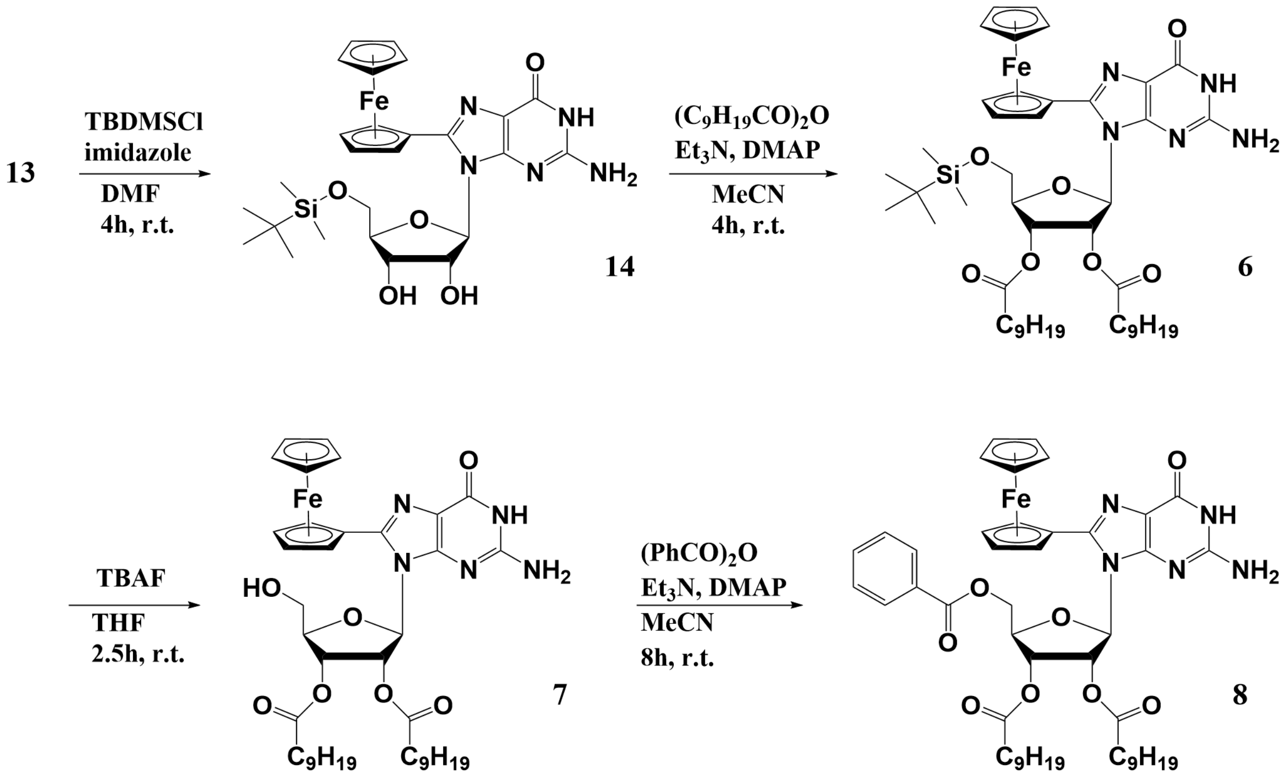

Scheme S4. Synthesis of guanosines **6-8**

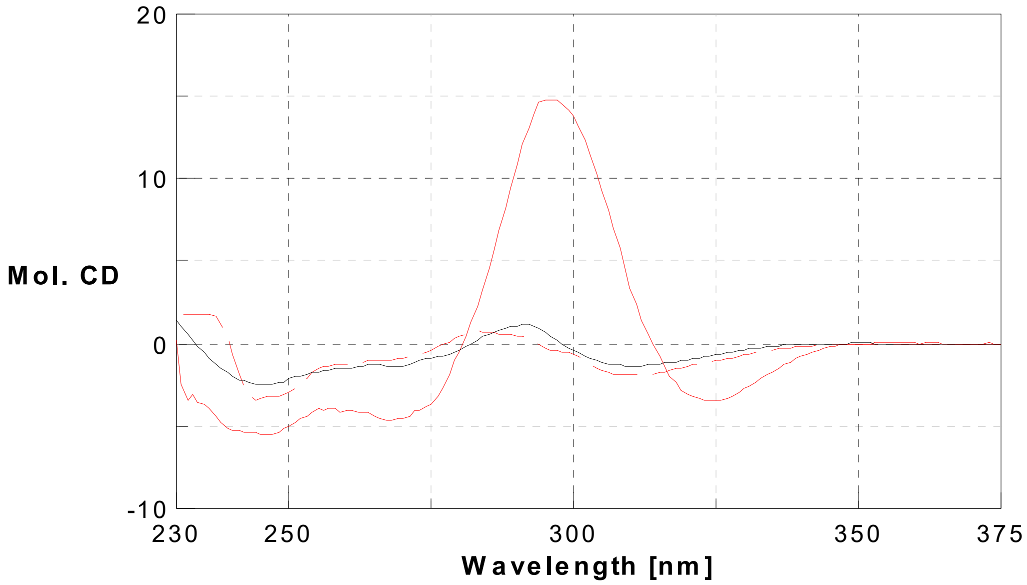

CD spectra recorded on 10 mM CH_2Cl_2 solutions of **8Ph5Fc** before (black trace) and after addition of [2.2.2] cryptand (red dotted trace) or excess of KI (red continuous trace). Path length 0.01 cm.

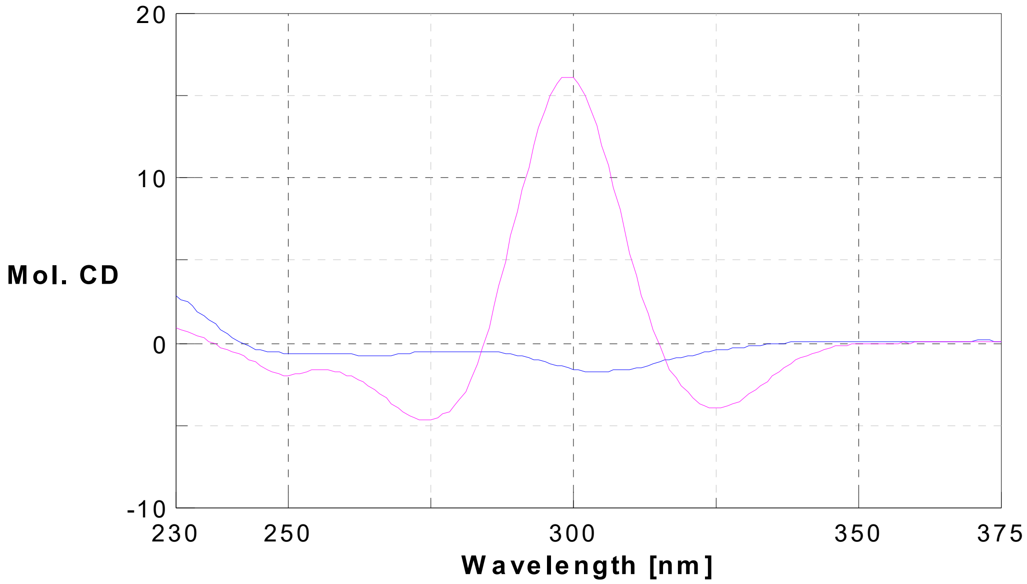

CD spectra recorded on 10 mM CH_2Cl_2 solutions of **8Ph5Si** before (blue trace) and after addition of KI (pink trace). Path length 0.01 cm.

Figure S1. Selected CD spectra showing the behaviour of guanosines **1**and **3** upon addition of [2.2.2] cryptand or excess KI.

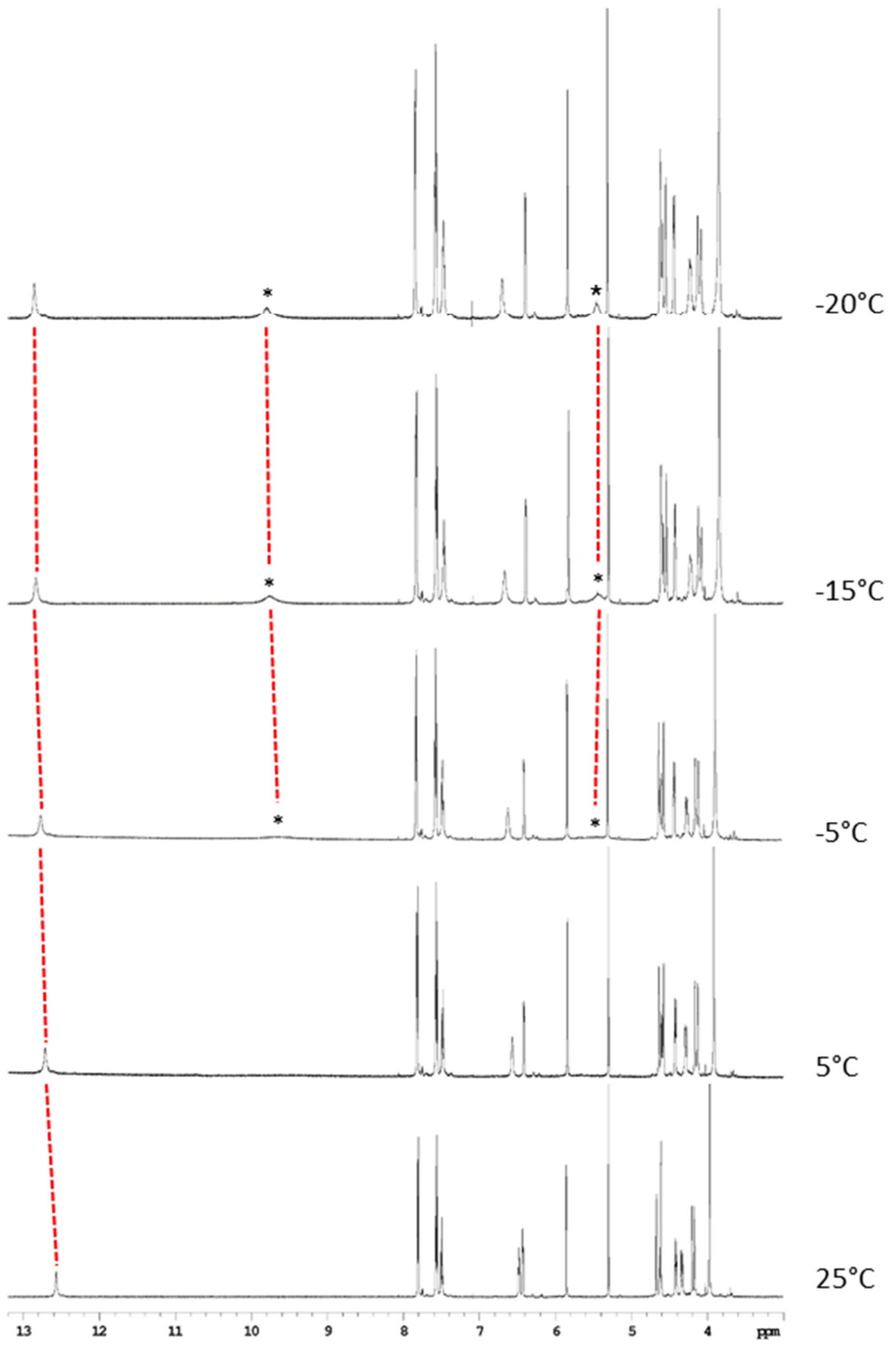

Figure S2. Downfield portion of the 600 MHz ^{1}H-NMR spectrum of **8Ph5Fc** (4.3 mM) at different temperatures in CD_2Cl_2. Amino protons are marked with asterisks.

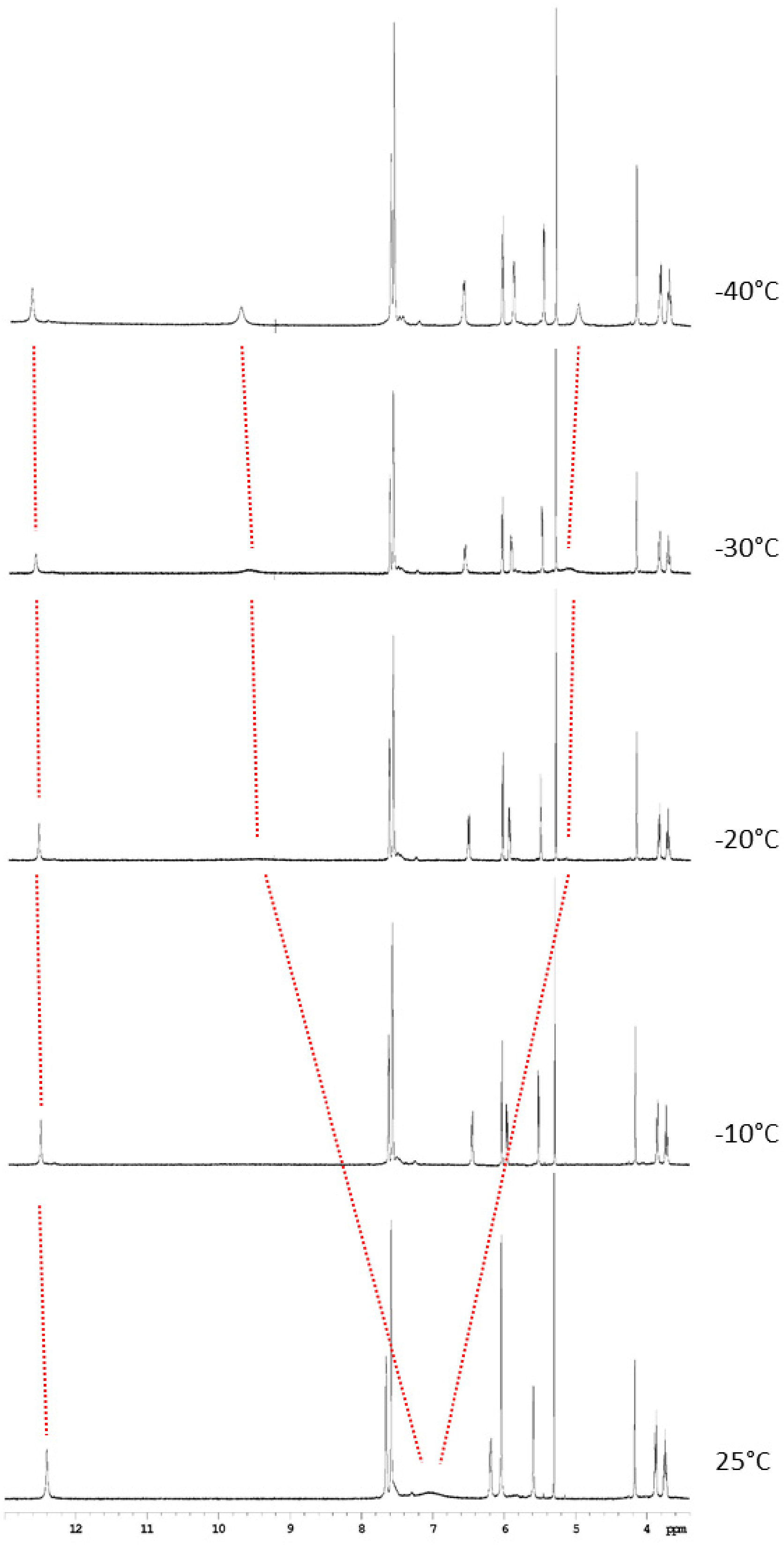

Figure S3. Downfield portion of the 600 MHz ^{1}H-NMR spectrum of **8Ph5OH** (5 mM) at different temperatures in CD_2Cl_2.

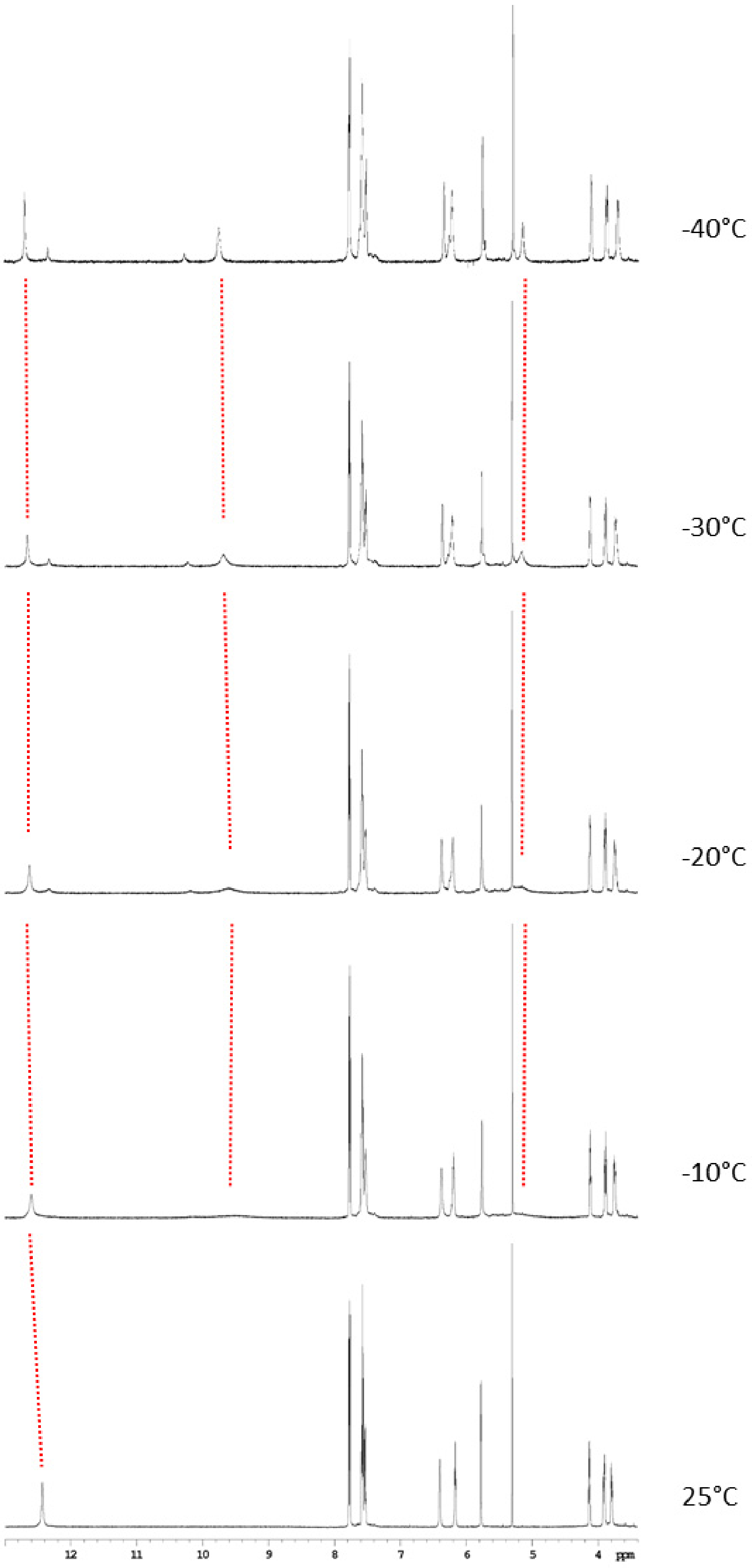

Figure S4. Downfield portion of the 600 MHz ^{1}H-NMR spectrum of **8Ph5Si** (6 mM) at different temperatures in CD_2Cl_2.

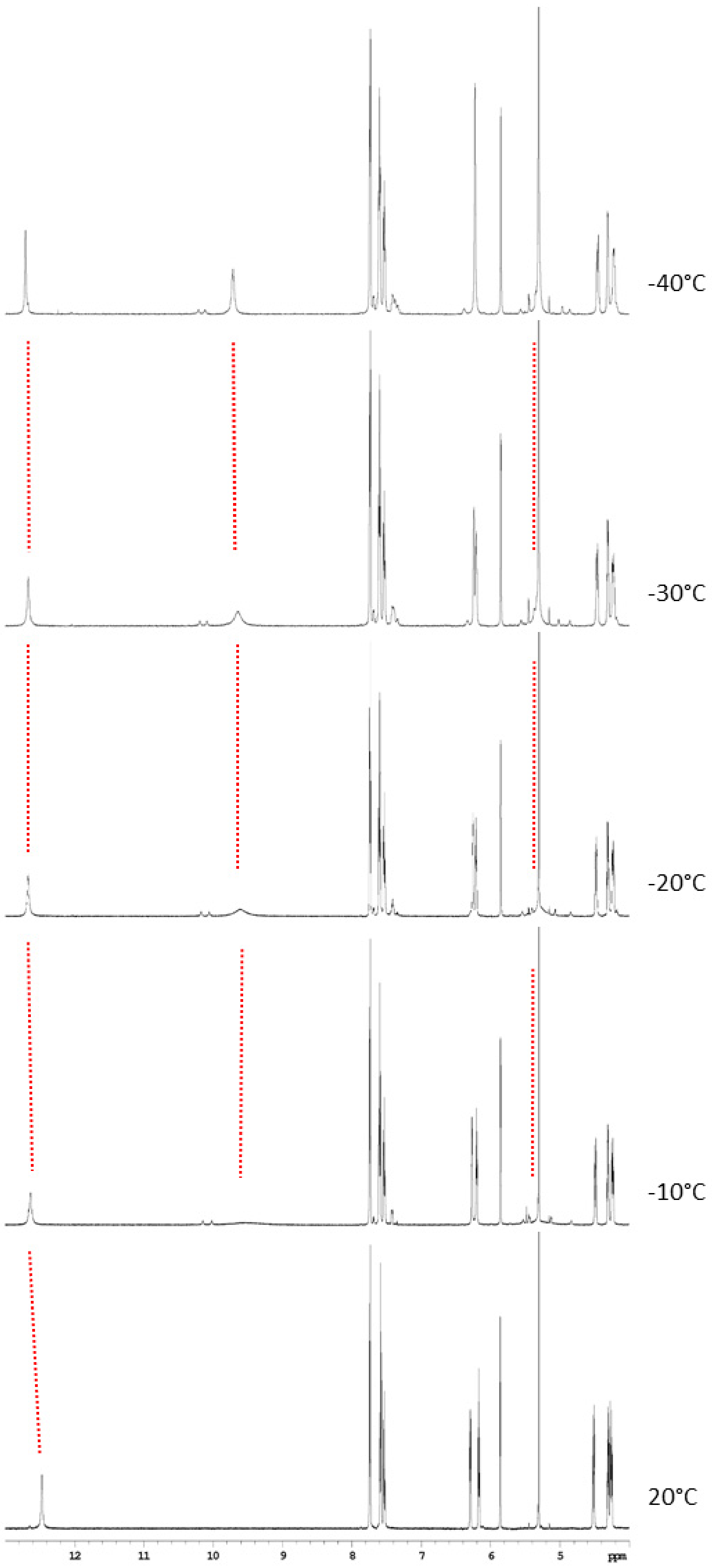

Figure S5. Downfield portion of the 600 MHz ^{1}H-NMR spectrum of **8Ph5C10** (7 mM) at different temperatures in CD_2Cl_2.

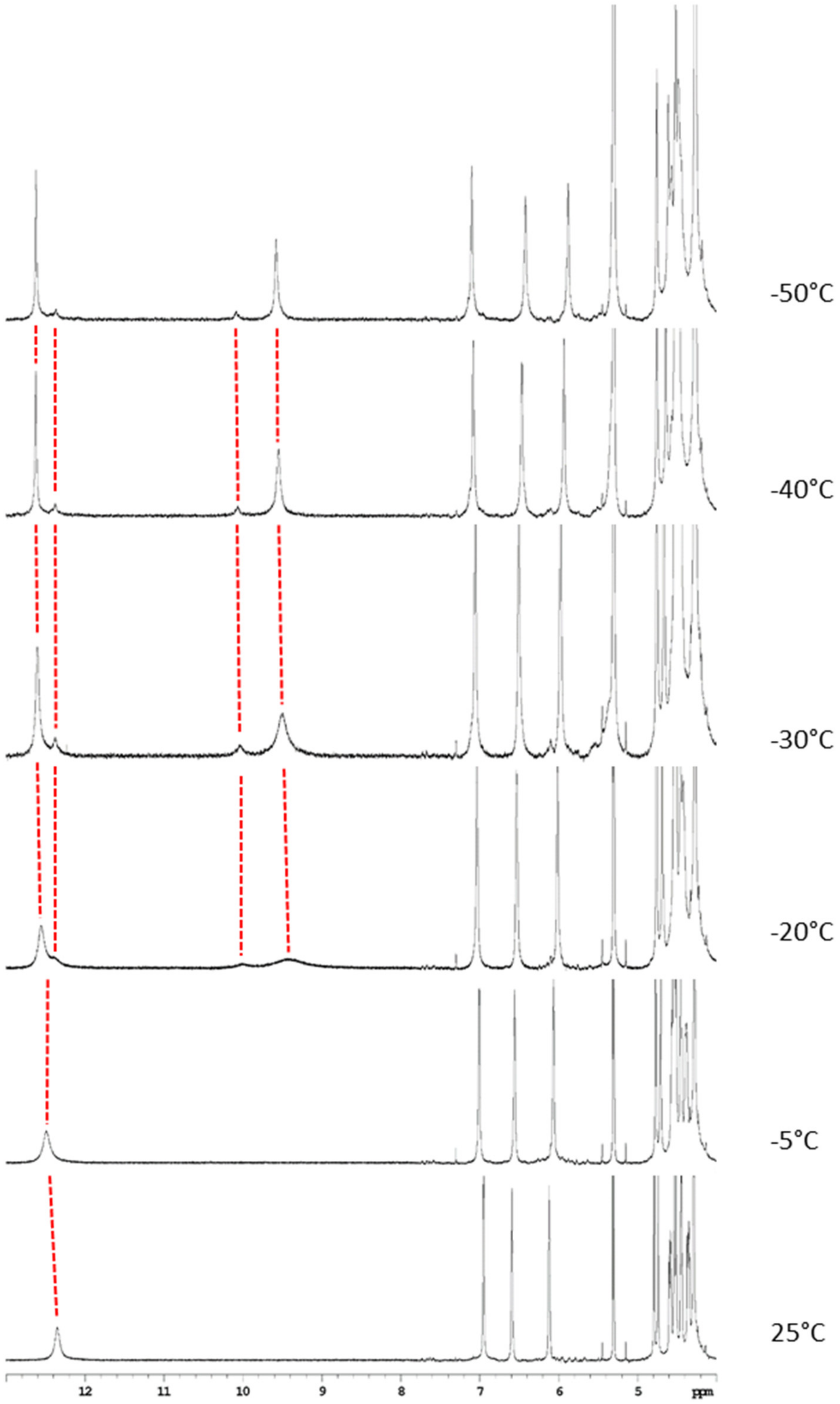

Figure S6. Downfield portion of the 600 MHz ^{1}H-NMR spectrum of **8Fc5C10** (4.5 mM) at different temperatures in CD_2Cl_2.

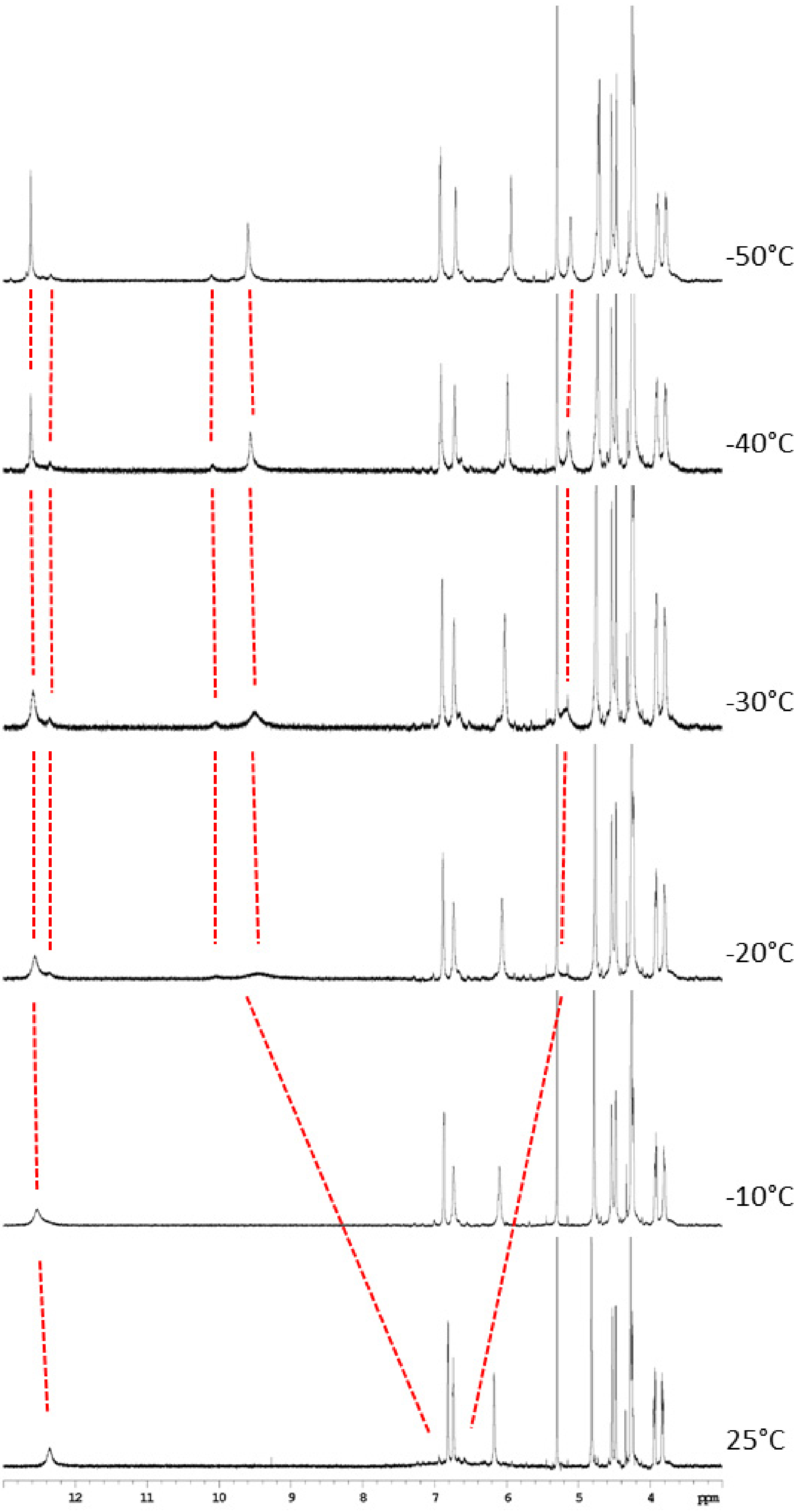

Figure S7. Downfield portion of the 600 MHz ^{1}H-NMR spectrum of **8Fc5Si** (6 mM) at different temperatures in CD_2Cl_2.

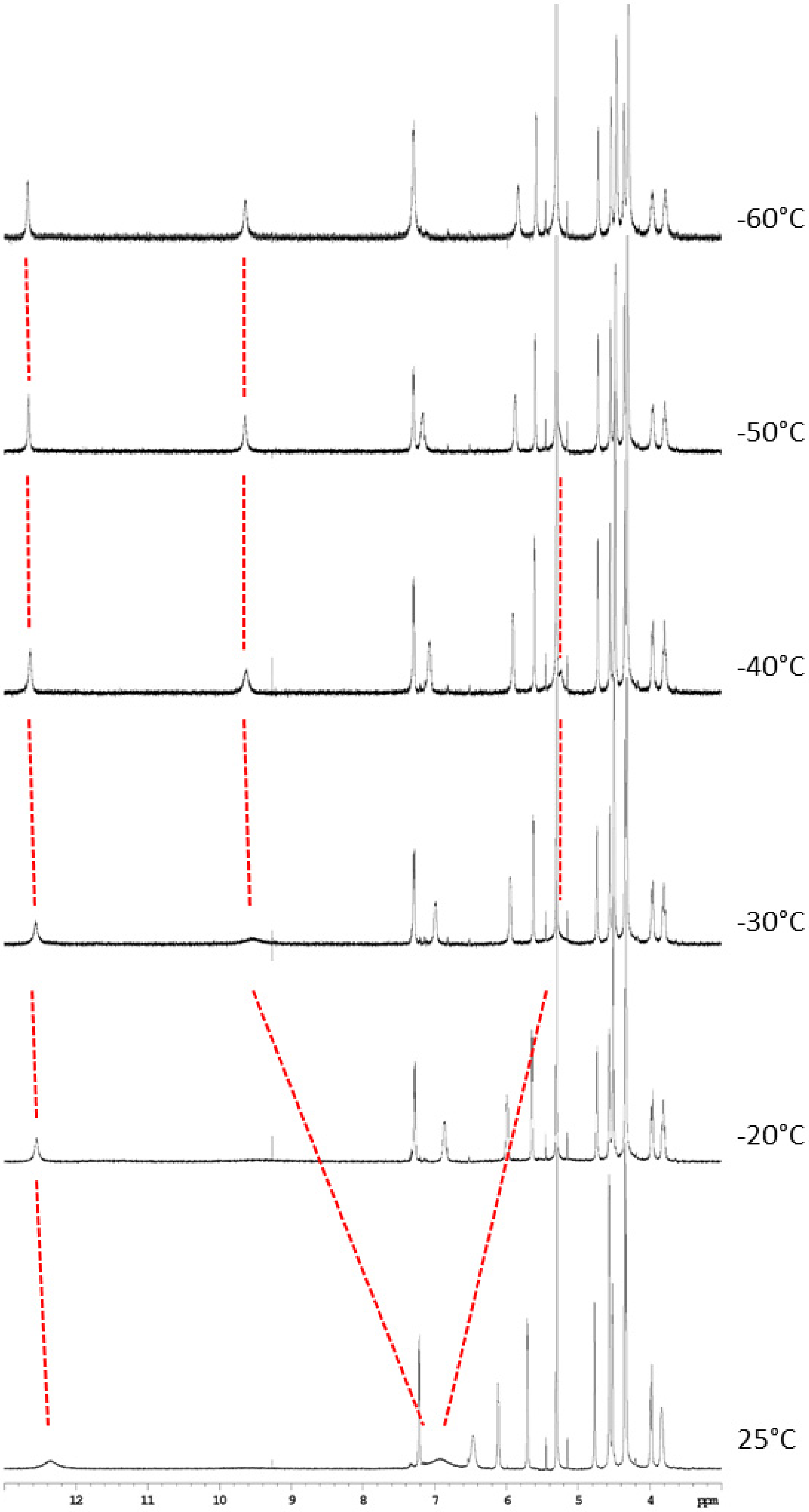

Figure S8. Downfield portion of the 600 MHz ^{1}H-NMR spectrum of **8Fc5OH** (7 mM) at different temperatures in CD_2Cl_2.

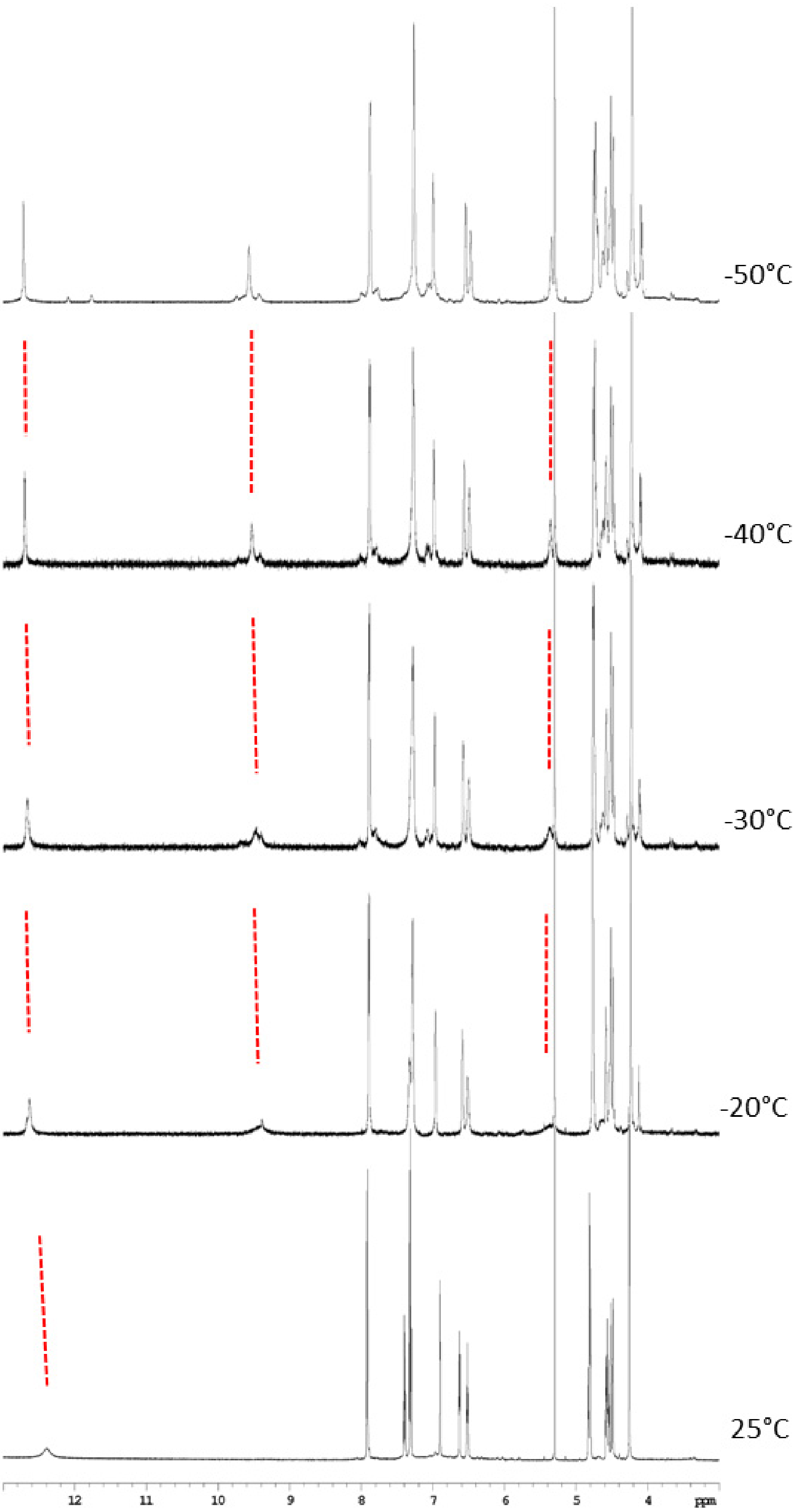

Figure S9. Downfield portion of the 600 MHz ^{1}H-NMR spectrum of **8Fc5Ph** (4 mM) at different temperatures in CD_2Cl_2.

Table S1. Room temp. chemical shifts* for sugar protons.

	Compound	solvent	δ(1')	δ(2')	δ(3')	δ sequence
**a**	**8Ph5Fc**	dmso	5.808	6.31	5.83	2'>3'>1'
		dcm	5.86	6.43	6.48	3'>2'>1'
**b**	**8Ph5OH**	dmso	5.78	5.496	5.186	1'>2'>3'
		dcm	6.2	6.04	5.588	1'>2'>3'
**c**	**8Ph5Si**	dmso	5.75	6.36	6.17	2'>3'>1'
		dcm	5.75	6.26	5.63	2'>1'>3'
**d**	**8Ph5C10**	dmso	-	-	-	-
		dcm	5.86	6.29	6.16	2'>3'>1'
**i**	**8Fc5C10**	dmso	6.75	6.58	5.74	1'>2'>3'
		dcm	6.95	6.59	6.12	1'>2'>3'
**e**	**8Fc5Si**	dmso	6.747	6.69	5.71	1'>2'>3'
		dcm	6.82	6.74	6.18	1'>2'>3'
**f**	**8Fc5OH**	dmso	6.86	5.61	5.3	1'>2'>3'
		dcm	7.22	6.12	5.71	1'>2'>3'
**g**	**8Fc5Ph**	dmso	6.77	6.63	5.99	1'>2'>3'
		dcm	6.9	6.63	6.51	1'>2'>3'
**h**	8-Bromo-2,3,5-tri-O-decanoylguanosine [1]	dmso	5.84	6.02	5.68	2'>1'>3'
**i**	2,3,5-tri-O-decanoylguanosine [2]	dmso	5.96	5.81	5.52	1'>2'>3'
**l**	2,3,5-tri-O-acetylguanosine	dmso [3]	6.007	5.809	5.515	1'>2'>3'
**m**	8-Bromoguanosine **9**	dmso [4]		4.89		
		dmso	5.68	5.01	4.14	1'>2'>3'
**n**	Guanosine	dmso [4]		4.36		
		dmso [3]	5.723	4.429	4.113	1'>2'>3'

* signals are referenced to residual solvent peak.

1. Prepared as described in Giorgi, T.; Lena, S.; Mariani, P.; Cremonini, M. A.; Masiero, S.; Pieraccini, S.; Rabe, J. P.; Samori, P.; Spada, G. P.; Gottarelli, G. *J. Am. Chem. Soc.* 2003, **125**, 14741

2. Prepared as described in Devetak, M.; Masiero, S.; Pieraccini, S.; Spada, G. P.; Copic, M.; Olenik, I. D. *Appl. Surf. Sci.* 2010, **256**, 2038

3. Spectral Database for Organic Compounds (https://sdbs.db.aist.go.jp/sdbs/cgi-bin/cre_disclaimer.cgi?REQURL=/sdbs/cgibin/cre_index.cgi&REFURL=http://www.bing.com/search%3fq=spectra+database+japan&form=PRASU1&src=IE11TR&pc=ASTE)

4. L. E. Buerkle, H. A. von Recumab, S. J. Rowan, *Chem. Sci.*, 2012, **3**, 564 and references cited therein.

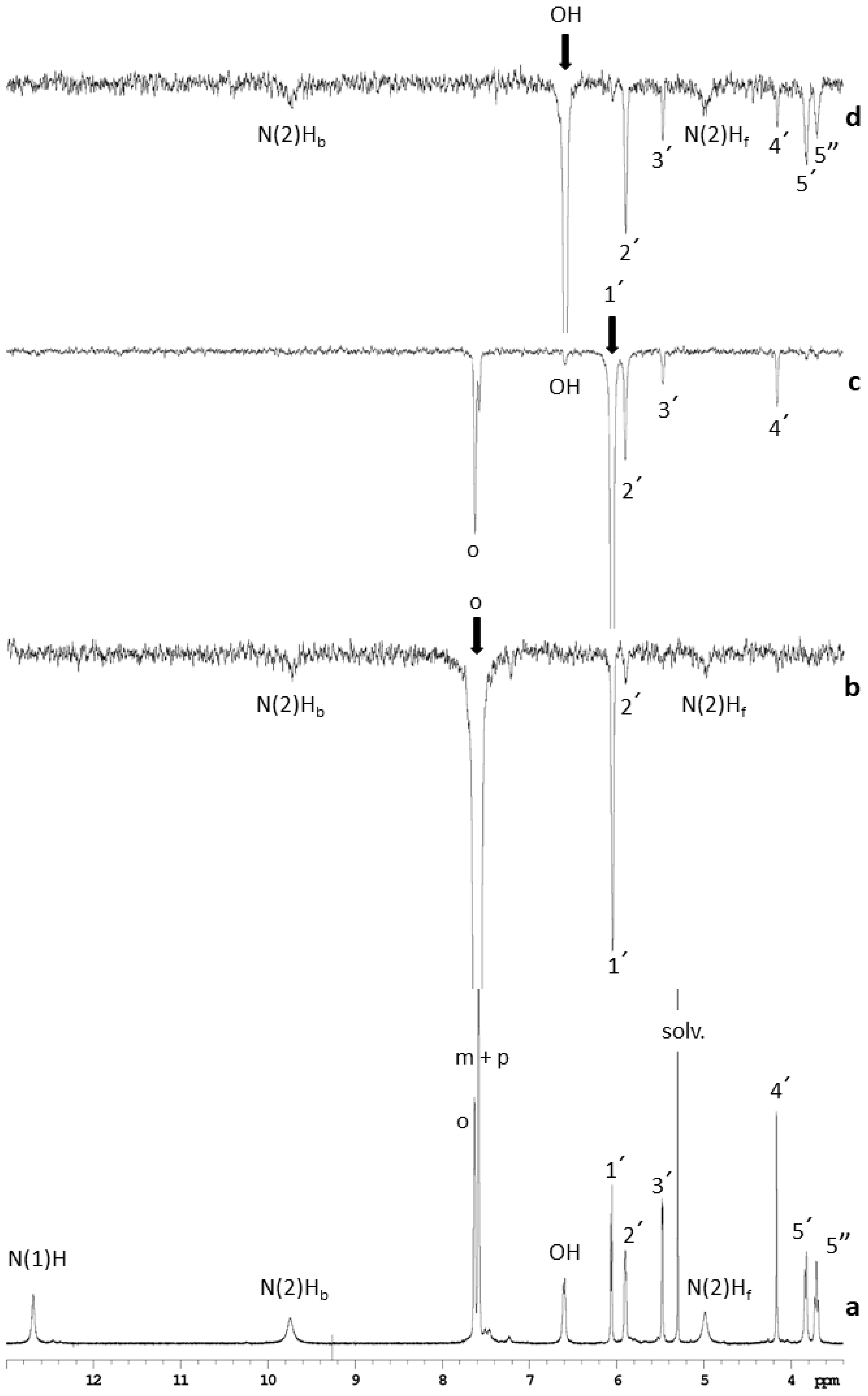

Figure S10. a) Downfield portion of the 600 MHz ^{1}H-NMR spectrum of **8Ph5OH** in CD_2Cl_2 (5 mM) and signals assignment (diastereotopic protons were not assigned); b), c), d): selected NOESY-1D spectra of the same sample. Irradiated signals are indicated by an arrow. In each NOE spectrum were used at least 1024 coadded transients, a recycle delay of 1 sec, a mixing time of 0.25 sec and a 20-50Hz shaped pulse. All spectra were recorded at -40°C.

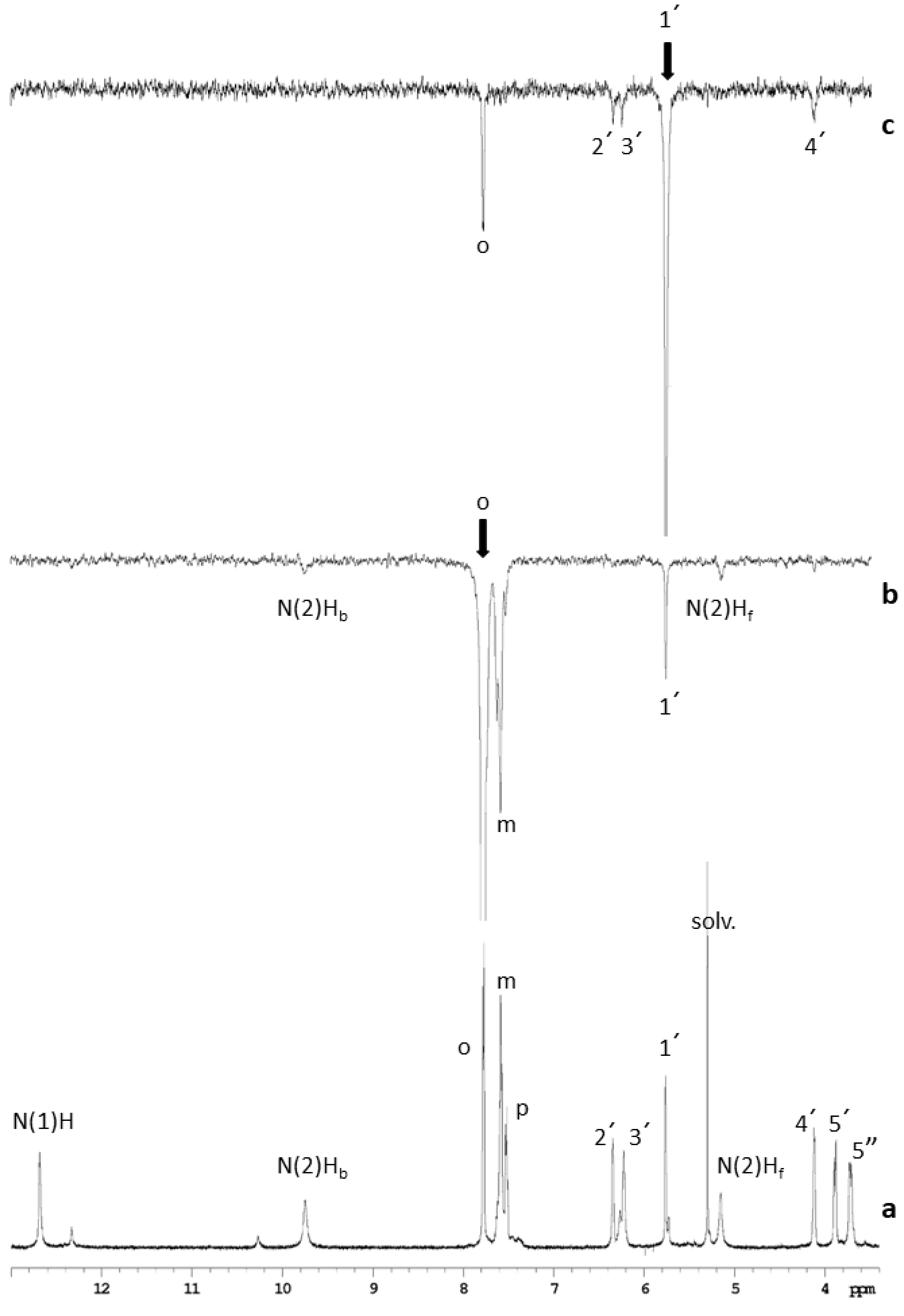

Figure S11. a) Downfield portion of the 600 MHz ^{1}H-NMR spectrum of **8Ph5Si** in CD_2Cl_2 (6 mM) and signals assignment (diastereotopic protons were not assigned); b), c): selected NOESY-1D spectra of the same sample. Irradiated frequencies are indicated by an arrow. In each NOE spectrum were used at least 512 coadded transients, a recycle delay of 1 sec, a mixing time of 0.2 sec and a 20-50Hz shaped pulse. All spectra were recorded at -40°C.

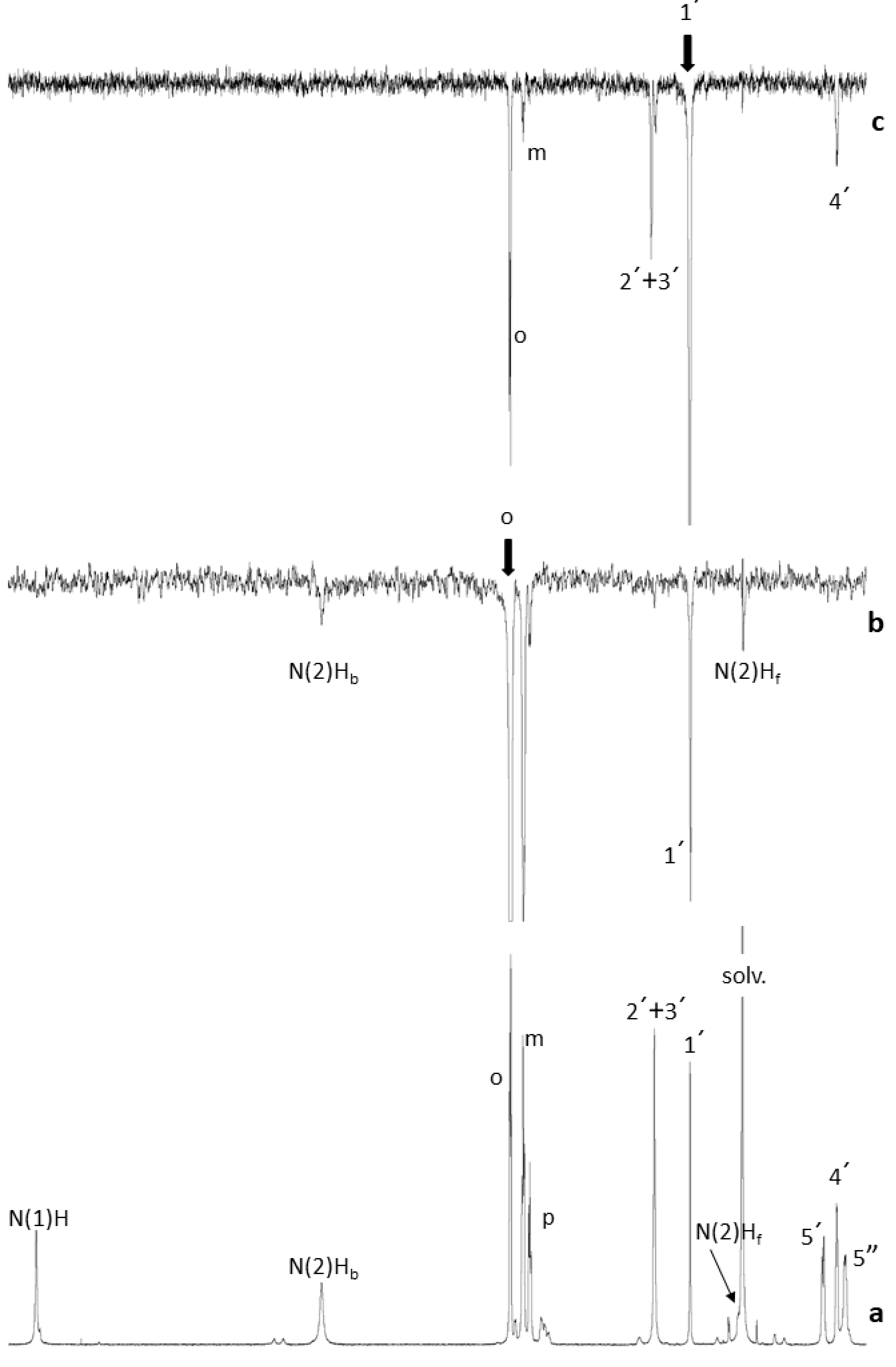

Figure S12. a) Downfield portion of the 600 MHz ^{1}H-NMR spectrum of **8Ph5C10** in CD_2Cl_2 (7 mM) and signals assignment (diastereotopic protons were not assigned); b), c): selected NOESY-1D spectra of the same sample. Irradiated signals are indicated by an arrow. In each NOE spectrum were used at least 256 coadded transients, a recycle delay of 1 sec, a mixing time of 0.4 sec and a 20-50Hz shaped pulse. All spectra were recorded at -40°C.

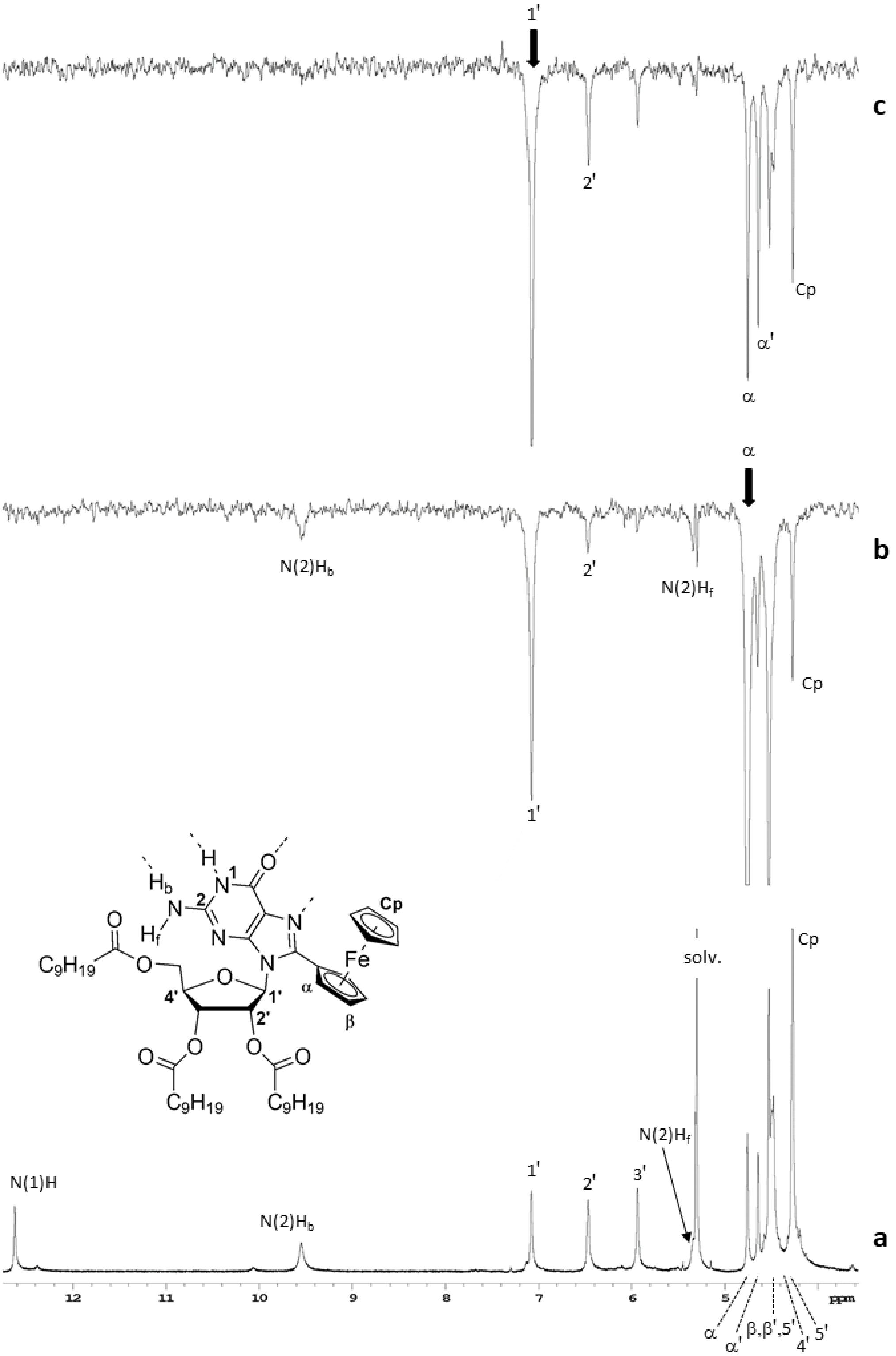

Figure S13. a) Downfield portion of the 600 MHz ^{1}H-NMR spectrum of **8Fc5C10** in CD_2Cl_2 (4.5mM) and signals assignment (diastereotopic protons were not assigned); b), c): selected NOESY-1D spectra of the same sample. Irradiated frequencies are indicated by an arrow. In each NOE spectrum were used 512 coadded transients, a recycle delay of 0.6 sec, a mixing time of 0.6 sec and a 50Hz shaped pulse. All spectra were recorded at -40°C.

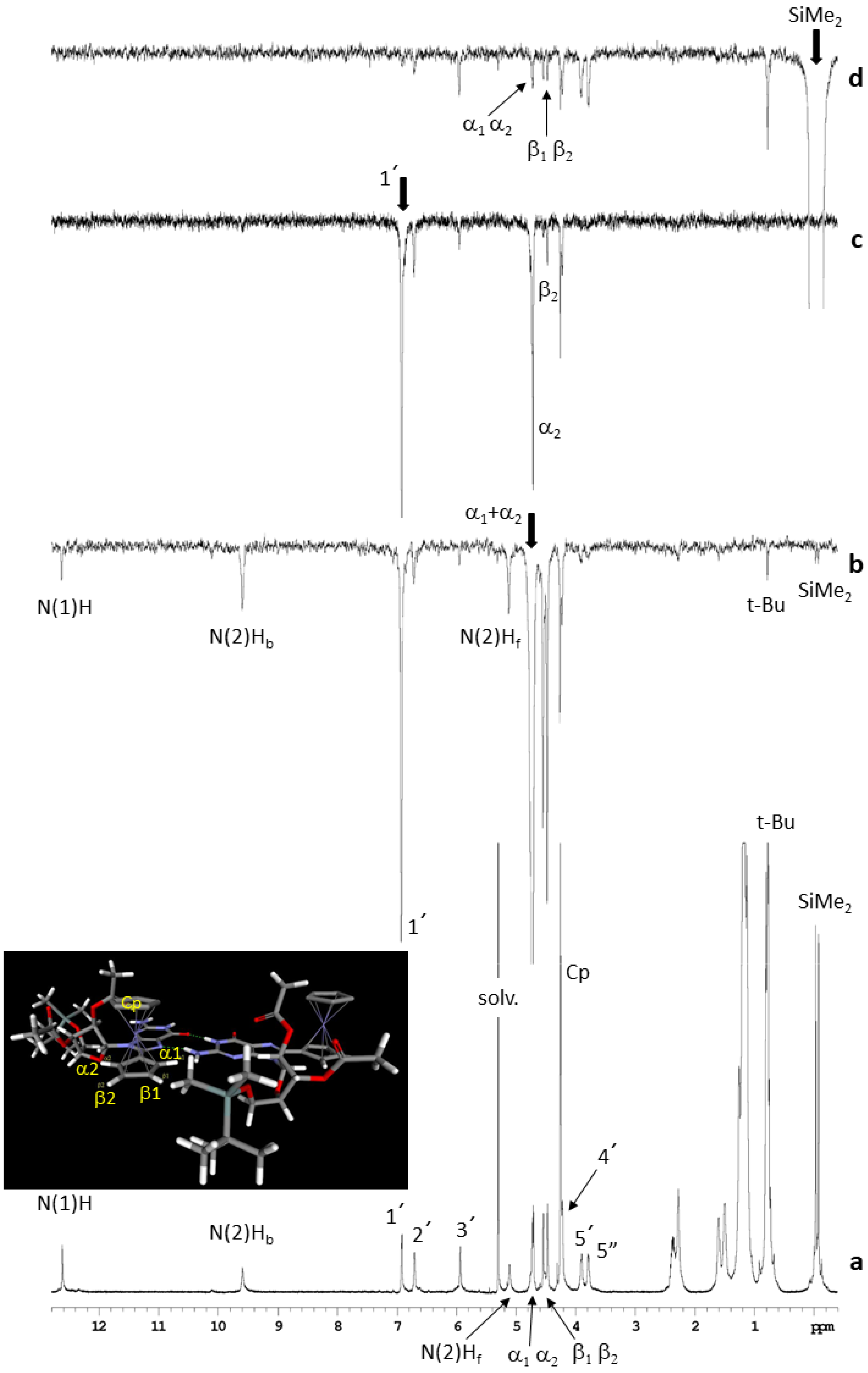

Figure S14. a) 600 MHz ^{1}H-NMR spectrum of **8Fc5Si** in CD_2Cl_2 (6 mM) and signals assignment (sugar diastereotopic protons were not assigned); b), c), d): selected NOESY-1D spectra of the same sample. Irradiated signals are indicated by an arrow. See inset for proton labeling. In each NOE spectrum were used at least 256 coadded transients, a recycle delay of 1 sec, a mixing time of 0.35 sec and a 20-80Hz shaped pulse. All spectra were recorded at -50°C.

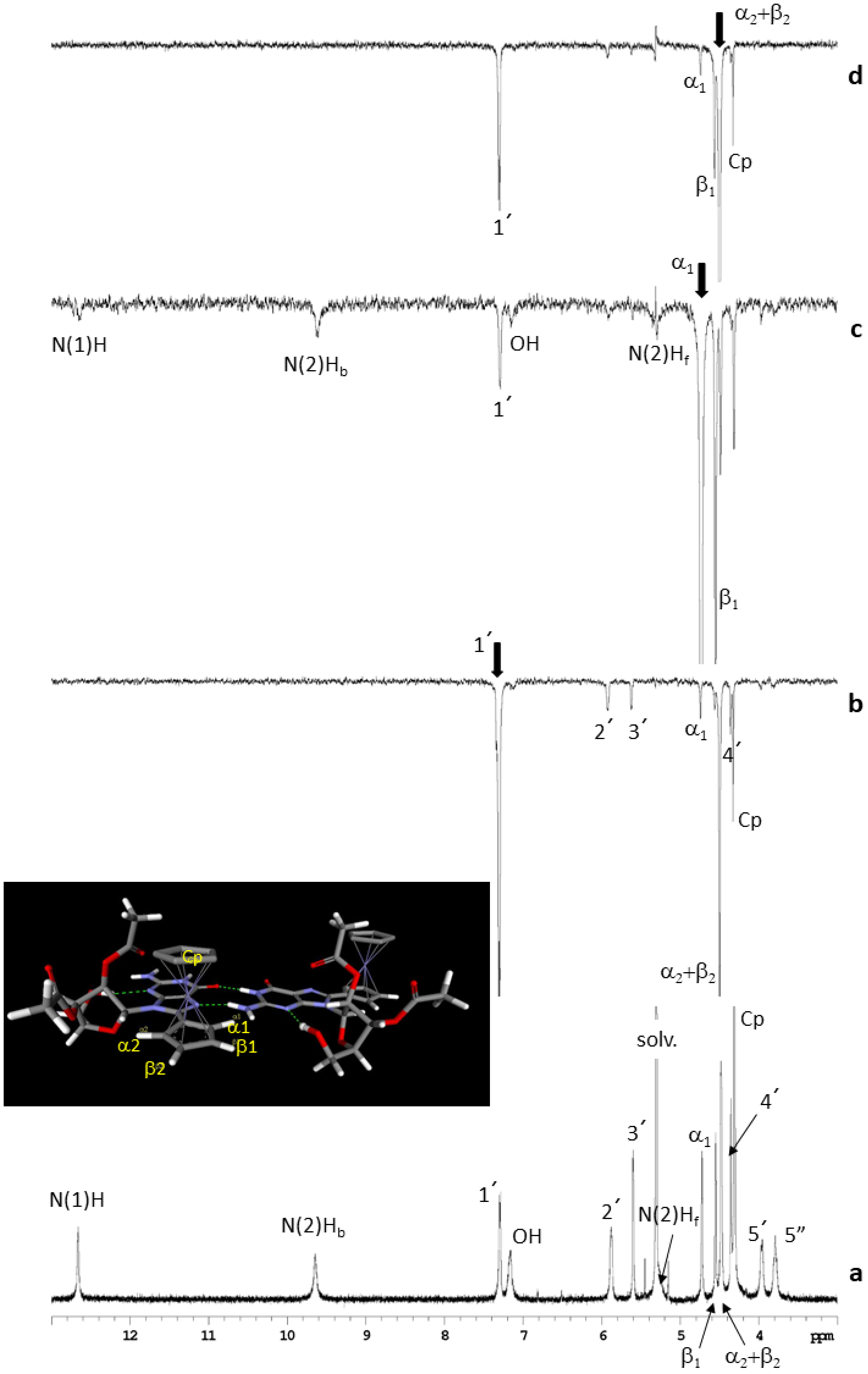

Figure S15. a) Downfield portion of the 600 MHz ^{1}H-NMR spectrum of **8Fc5OH** in CD_2Cl_2 (7 mM) and signals assignment (ribose diastereotopic protons were not assigned); b), c), d): selected NOESY-1D spectra of the same sample. Irradiated frequencies are indicated by an arrow. See inset for proton labeling. In each NOE spectrum were used at least 128 coadded transients, a recycle delay of 1 sec, a mixing time of 0.4 sec and a 20-50Hz shaped pulse. All spectra were recorded at -50°C.

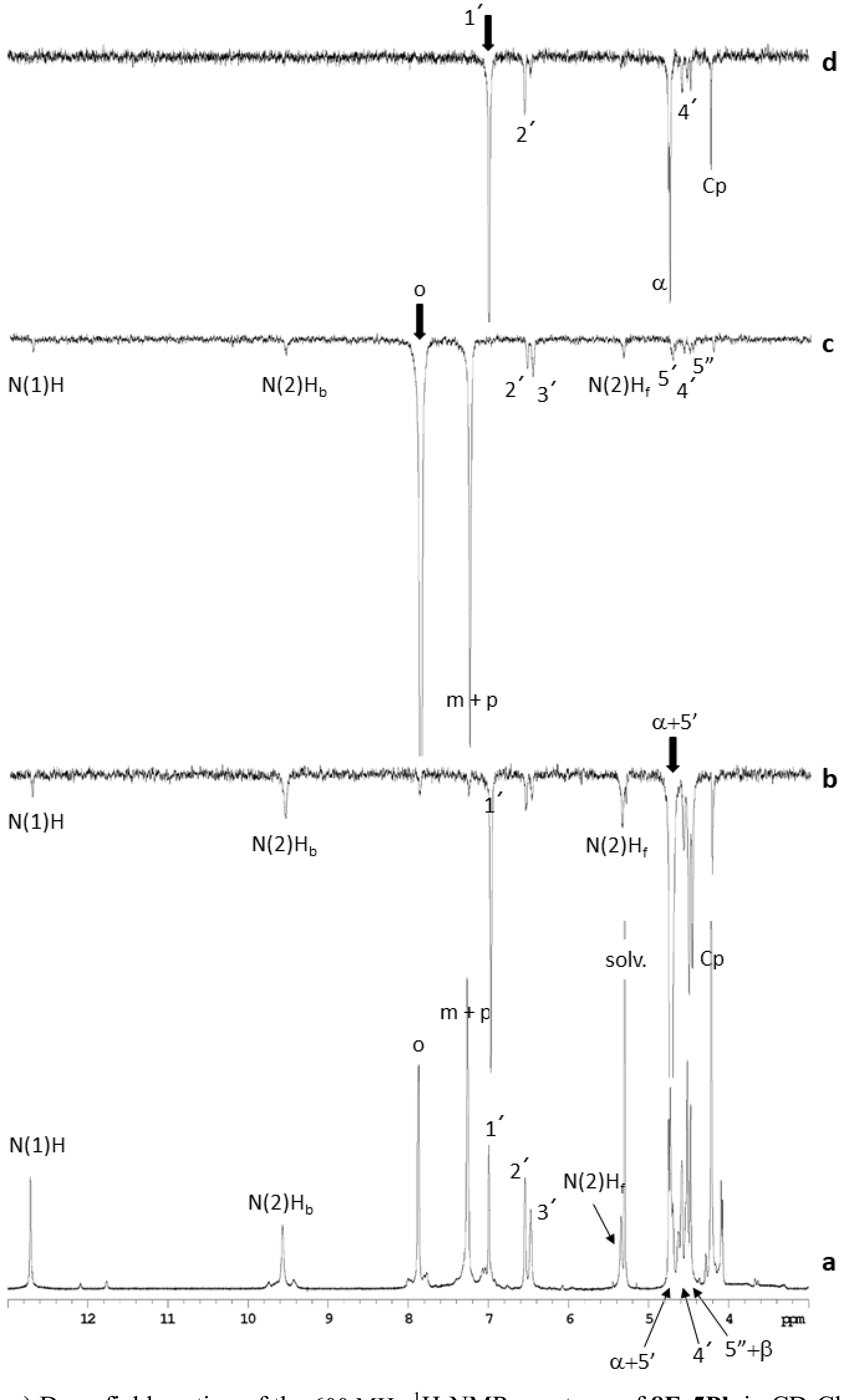

Figure S16. a) Downfield portion of the 600 MHz ^{1}H-NMR spectrum of **8Fc5Ph** in CD_2Cl_2 (4 mM) and signals assignment (diastereotopic protons were not assigned); b), c), d): selected NOESY-1D spectra of the same sample. Irradiated protons are indicated by an arrow. In each NOE spectrum were used at least 256 coadded transients, a recycle delay of 1 sec, a mixing time of 0.4 sec and a 20-50Hz shaped pulse. All spectra were recorded at -50°C.

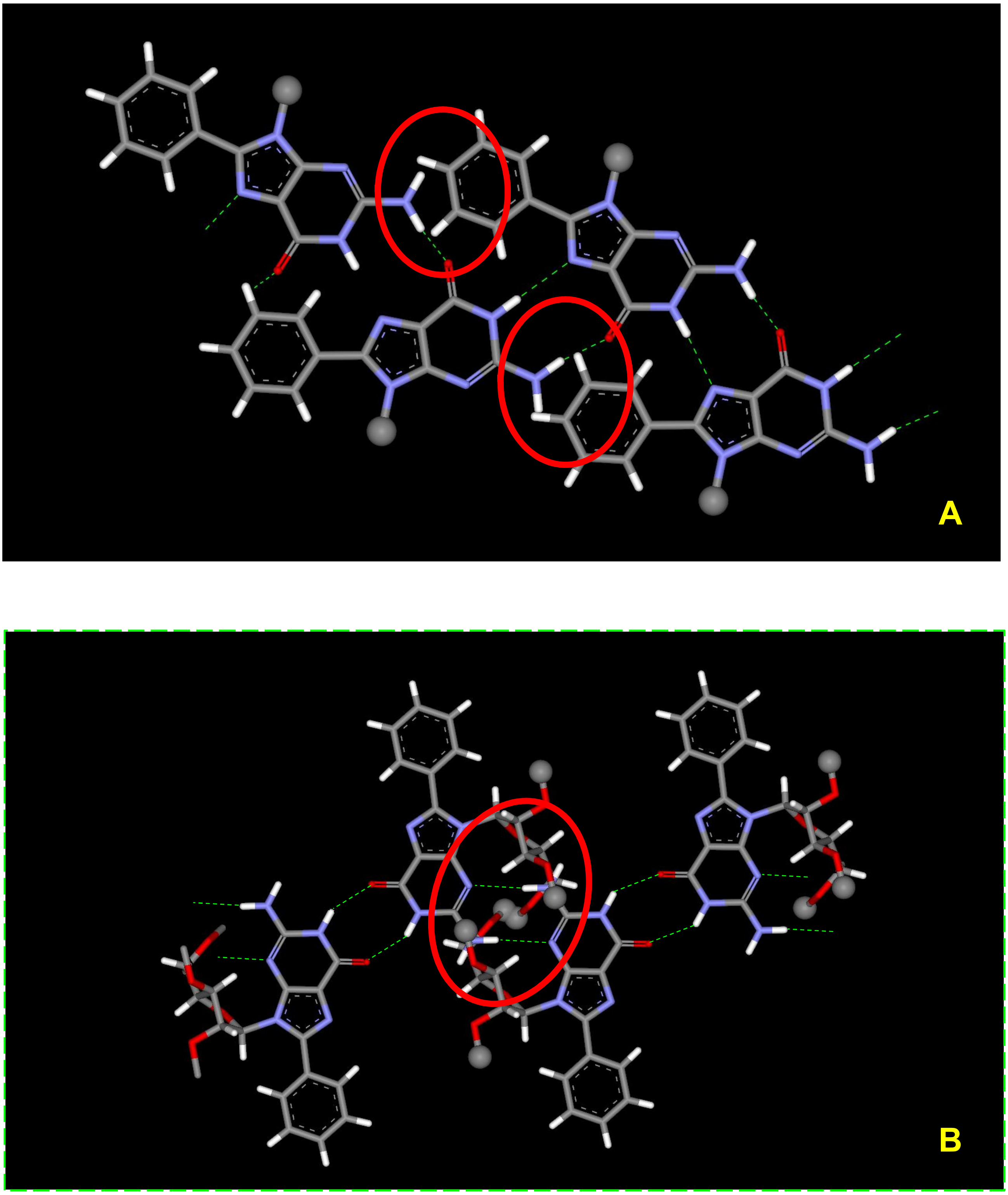

Figure S17. Sketches of ribbon-like structures A and B hypothetically formed by 8-substituted guanosine in syn conformation. Sterically overcrowded areas are circled. Furthermore, both structures are incompatible with observed NOEs.

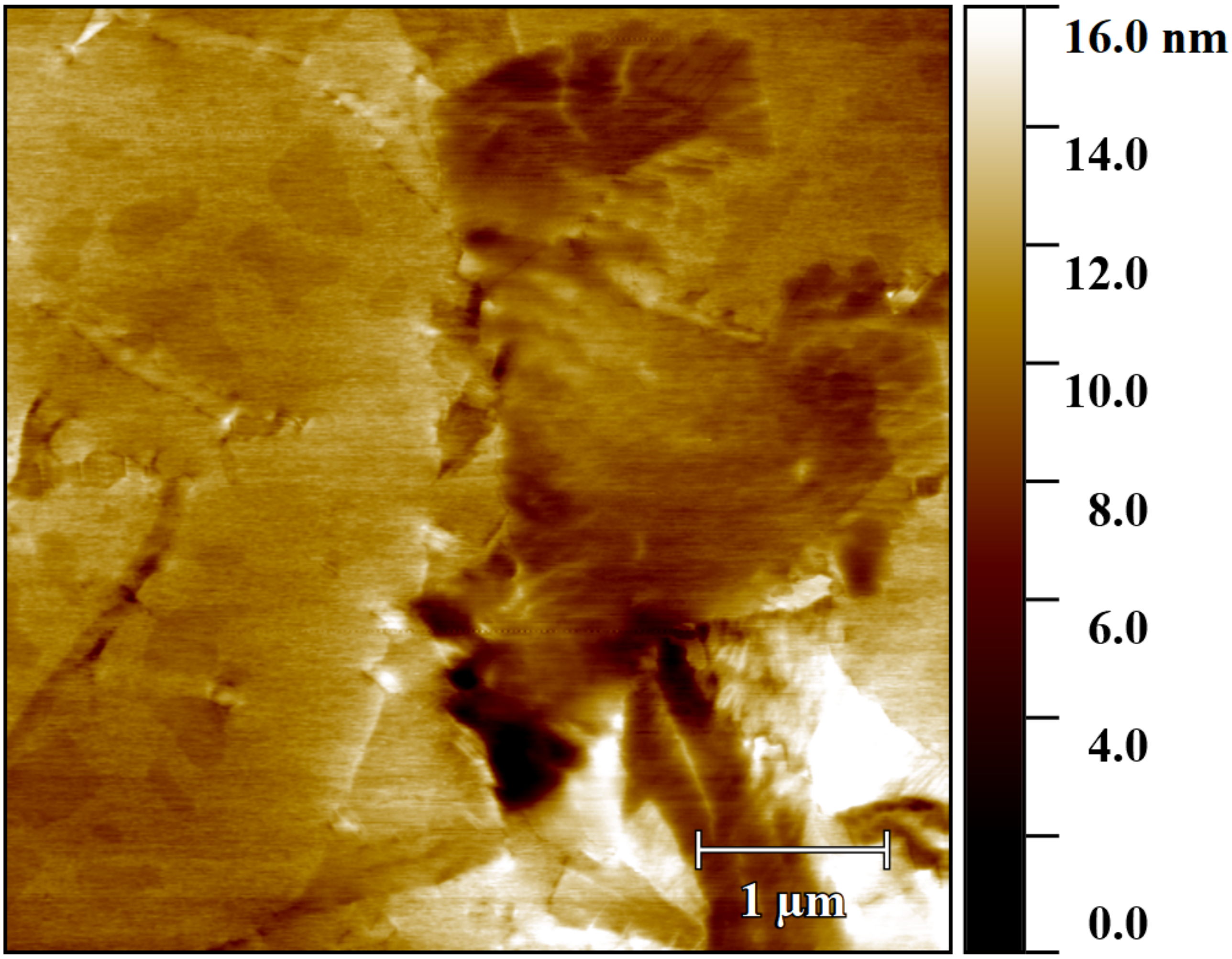

Figure S18. AFM image of the amorphous film obtained from **8Ph5C10** by drop-casting from CH_2Cl_2.

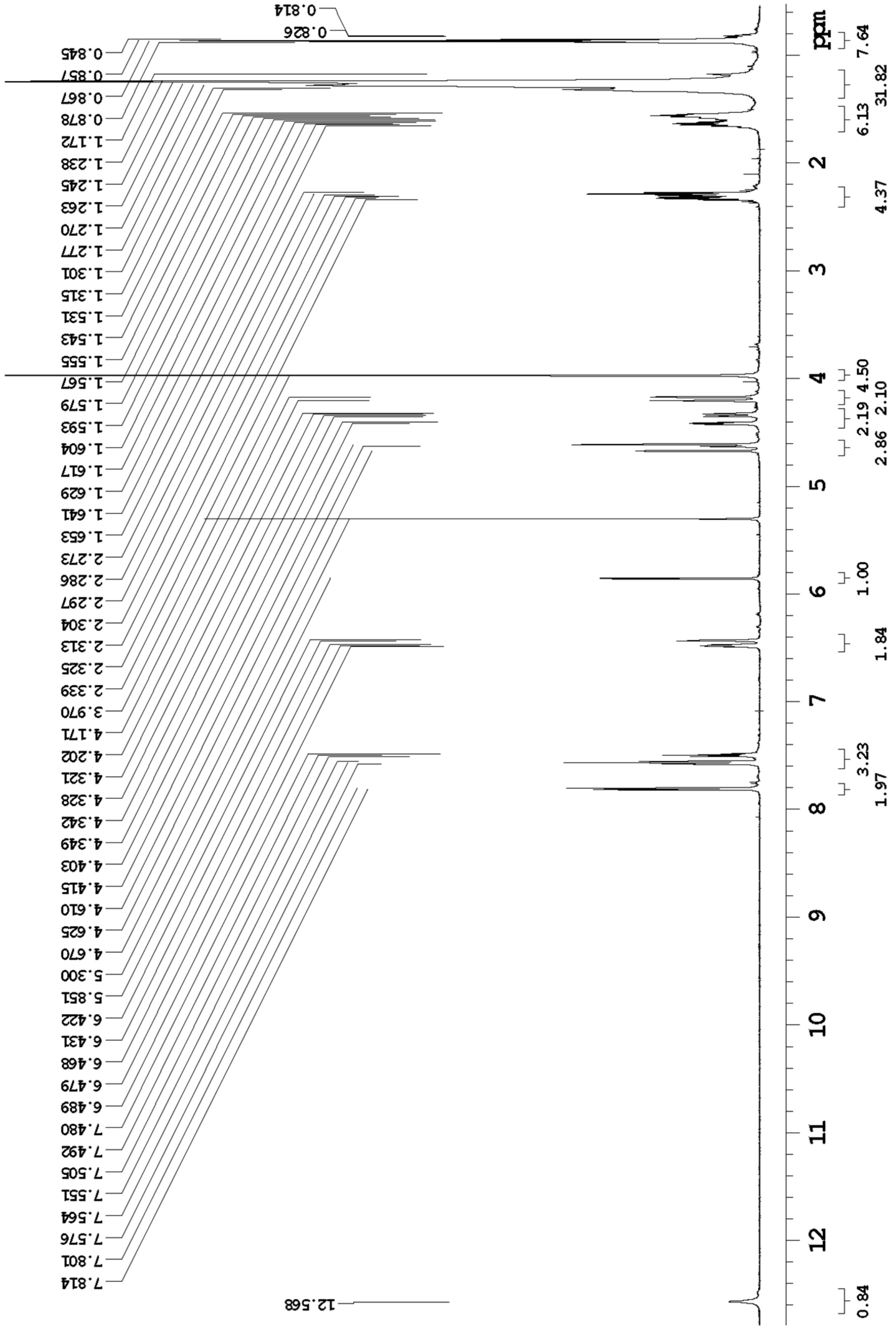

^{1}H-NMR (CD_2Cl_2, 600 MHz) of **8Ph5Fc**

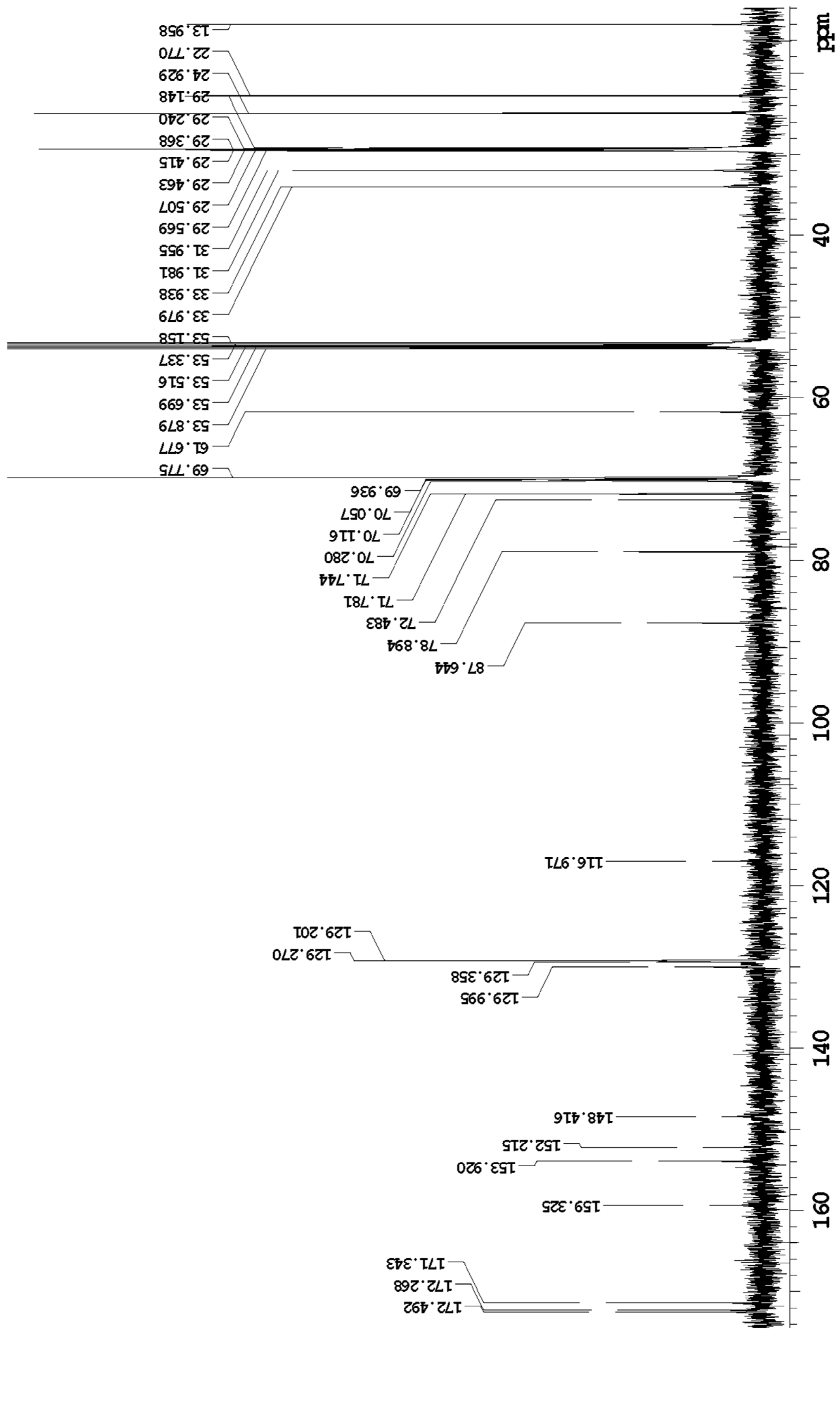

$^{13}C\{1H\}$NMR (CD_2Cl_2, 600 MHz) of **8Ph5Fc**

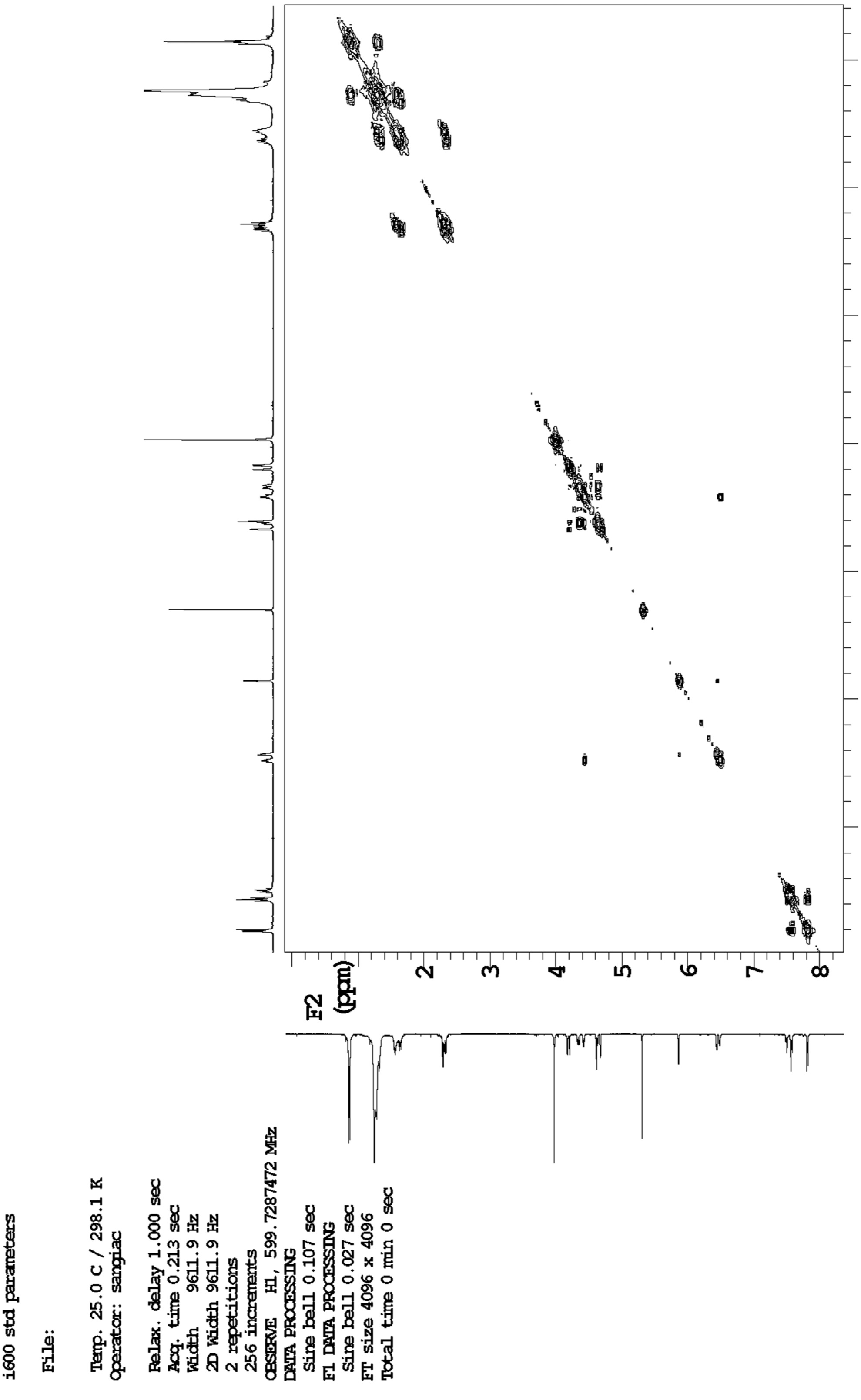

gCOSY spectrum (CD_2Cl_2, 600 MHz) of **8Ph5Fc**

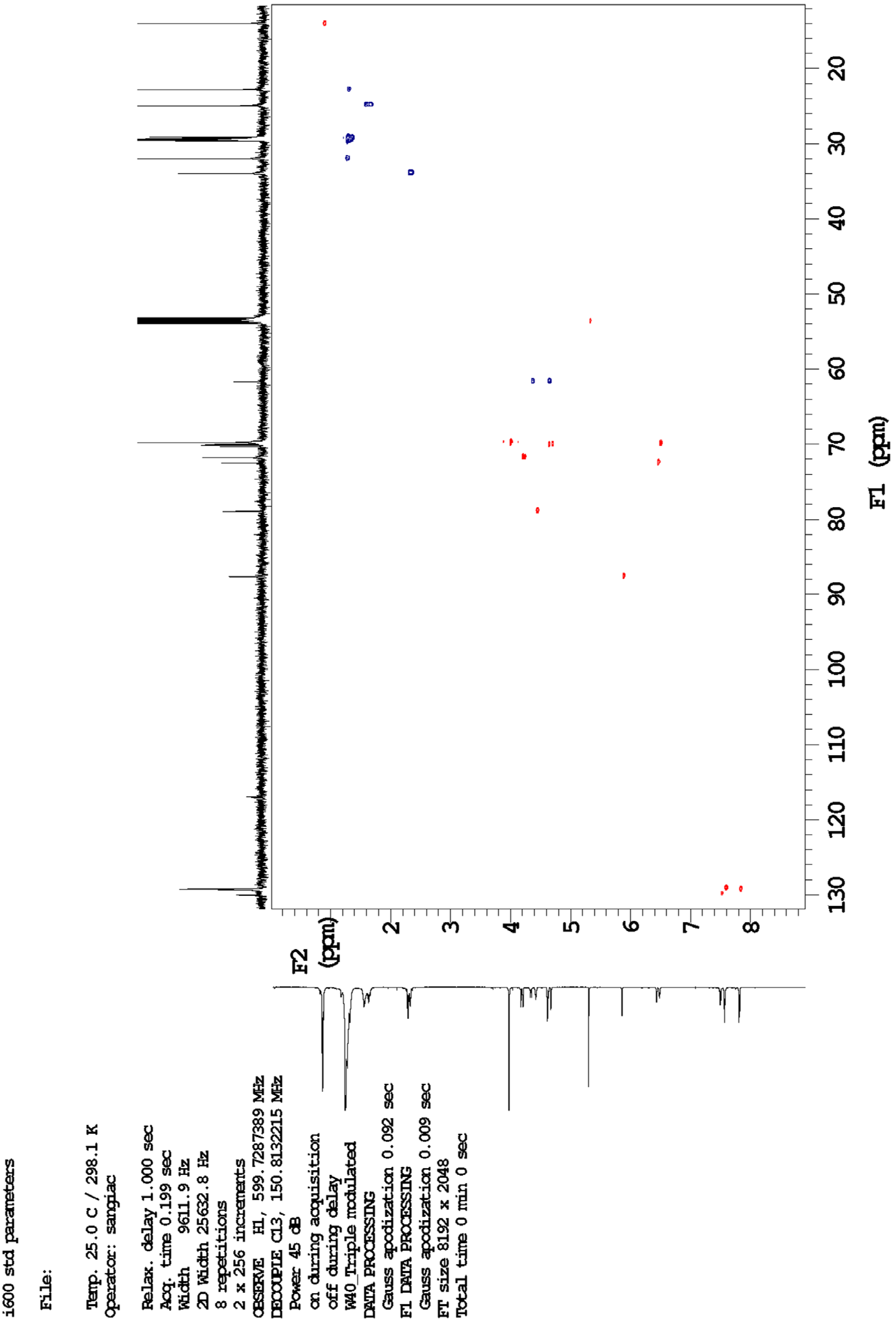

gHSQC spectrum (CD_2Cl_2, 600 MHz) of **8Ph5Fc**

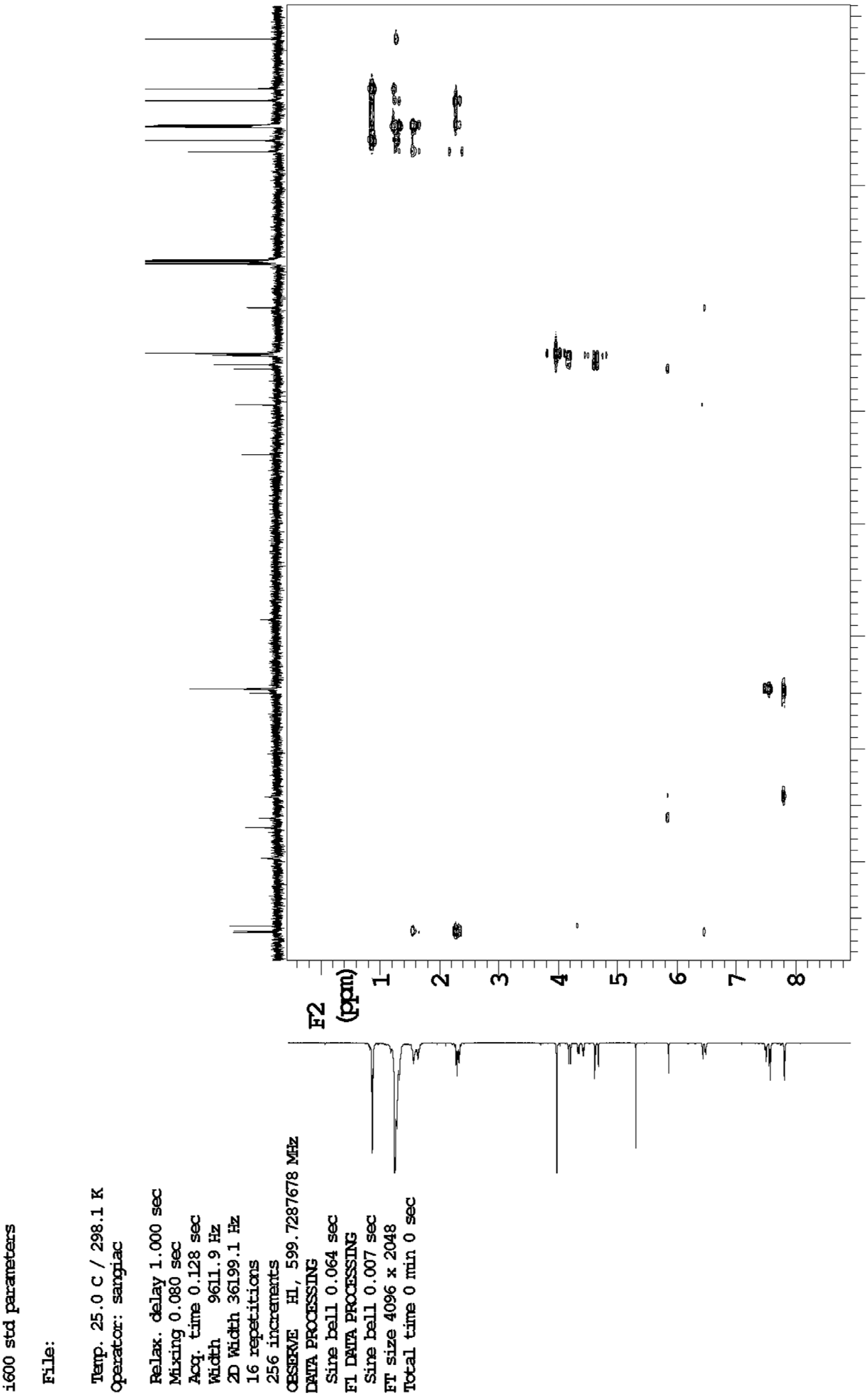

gHMBC spectrum (CD_2Cl_2, 600 MHz) of **8Ph5Fc**

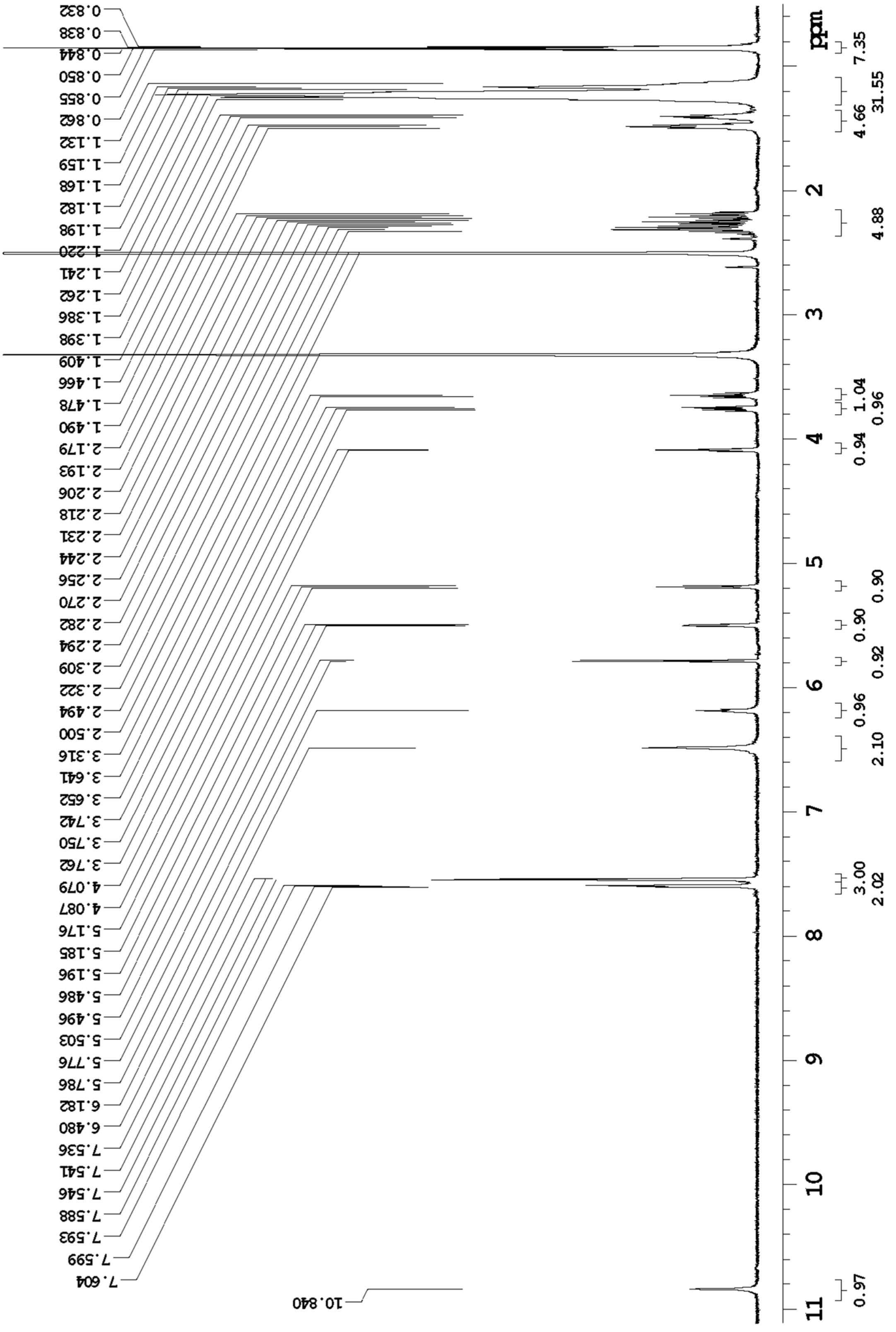

^{1}H-NMR (dmso-d6, 600 MHz) of **8Ph5OH**

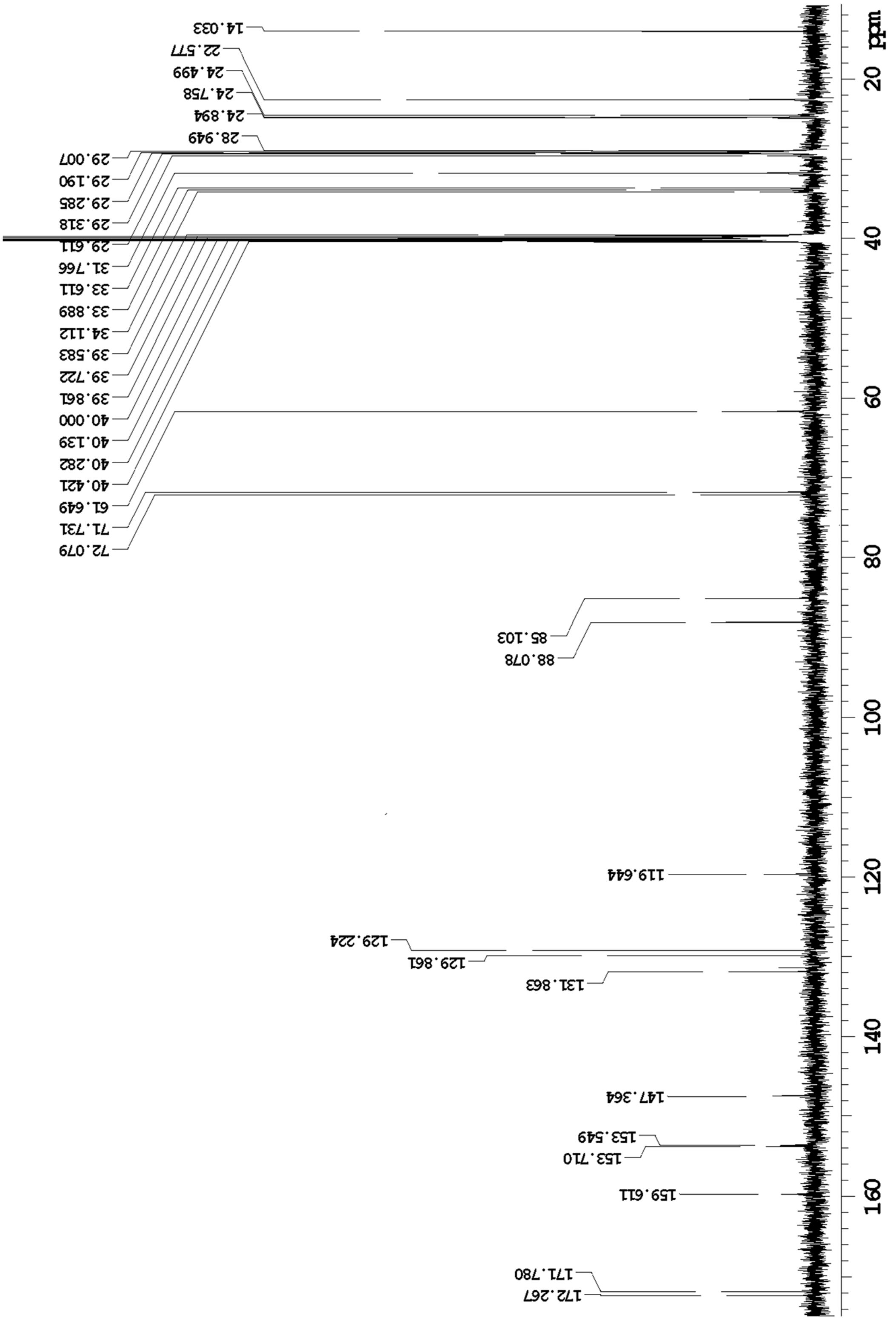

^{13}C{1H}NMR (dmso-d$_6$, 600 MHz) of **8Ph5OH**

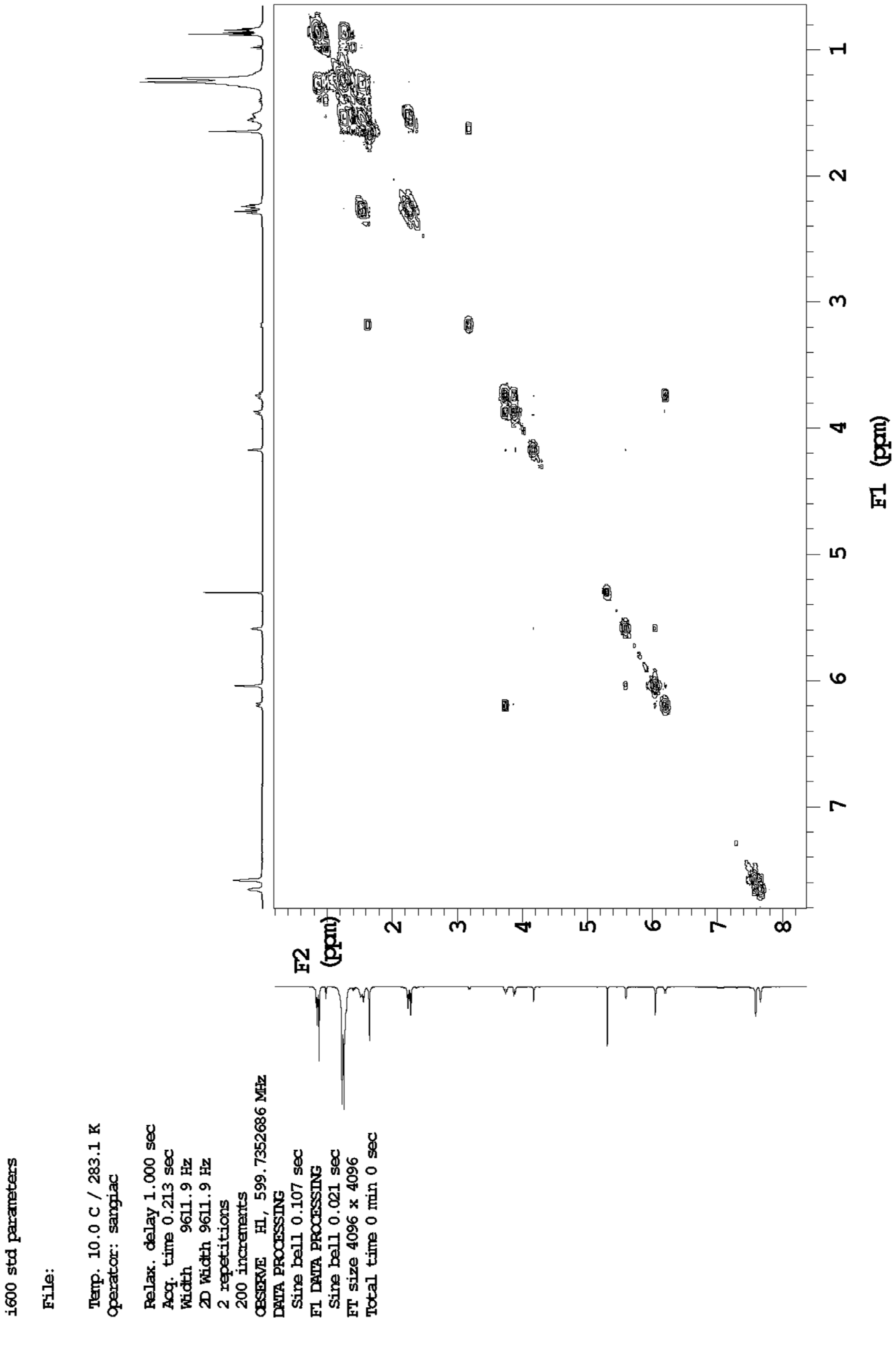

gCOSY spectrum (CD_2Cl_2, 600 MHz) of **8Ph5OH**

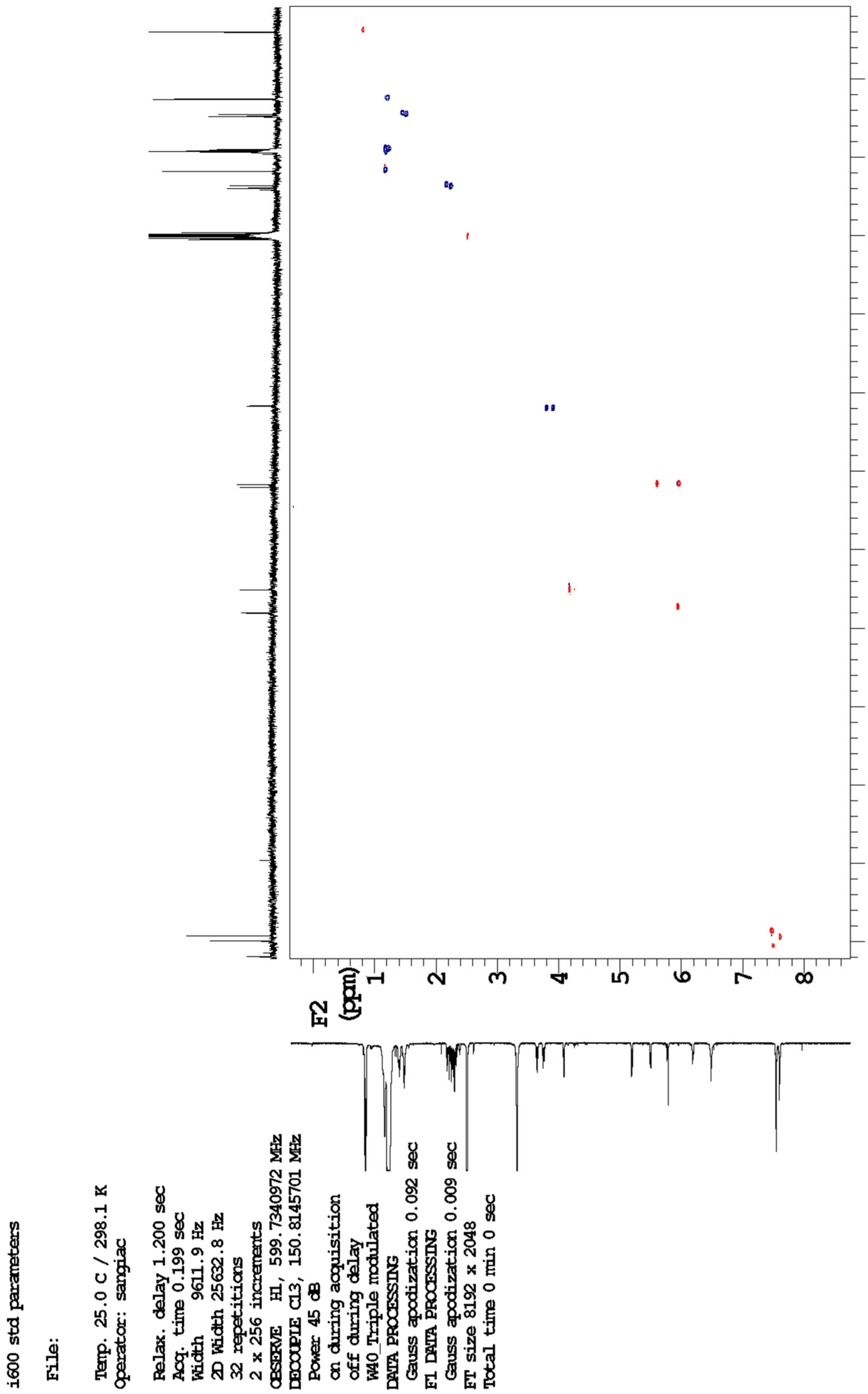

gHSQC spectrum (dmso-d_6, 600 MHz) of **8Ph5OH**

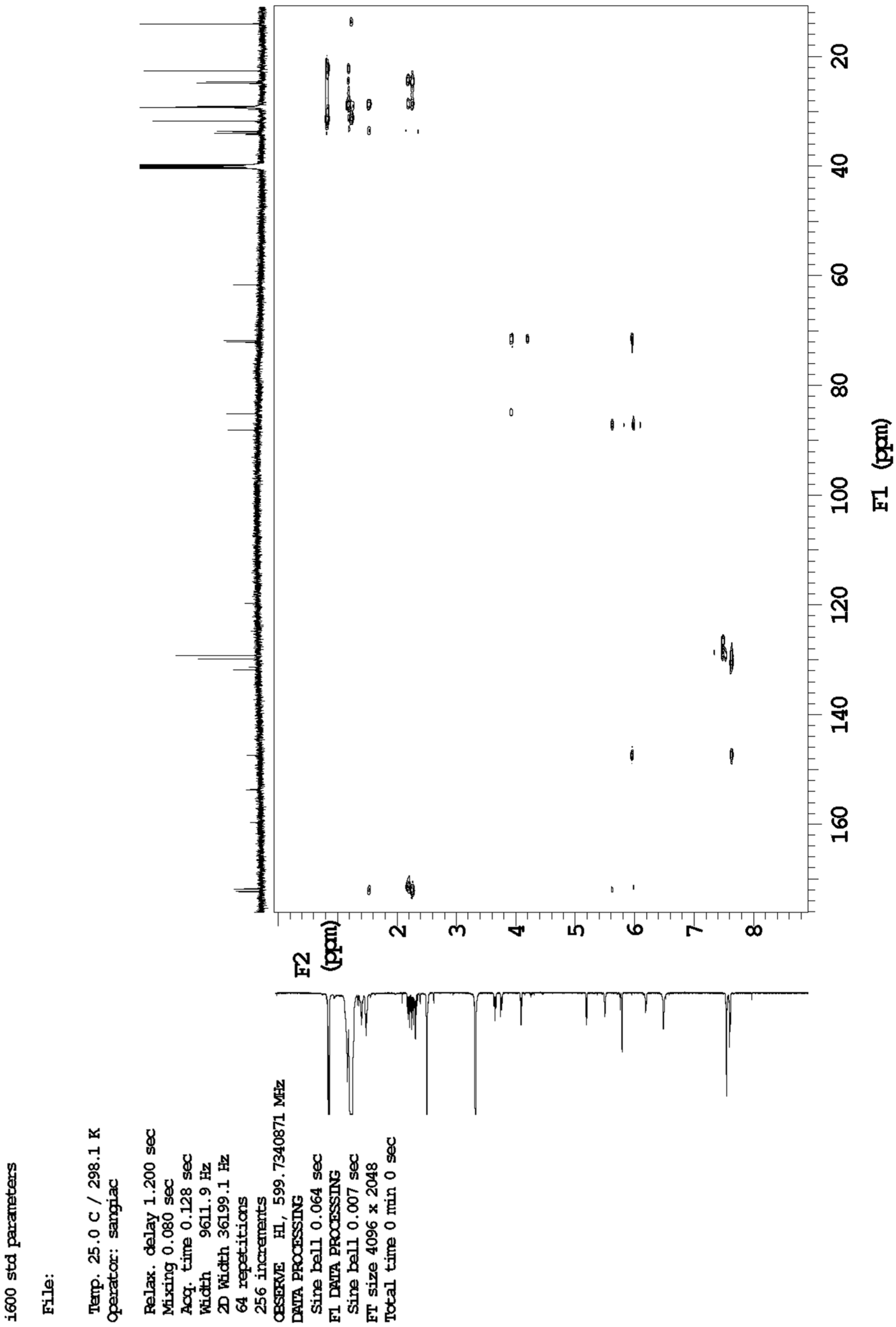

gHMBC spectrum (dmso-d_6, 600 MHz) of **8Ph5OH**

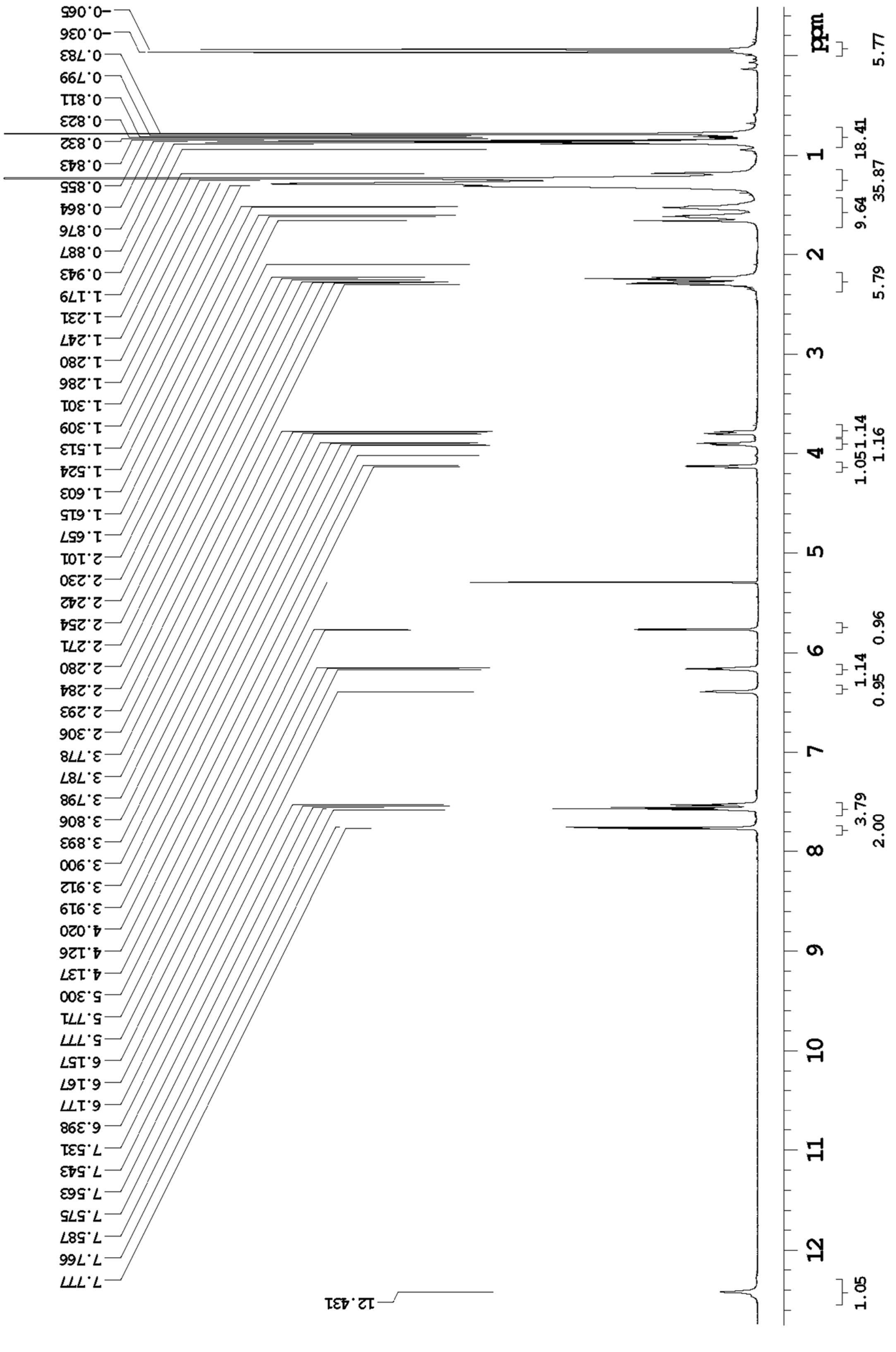

^{1}H-NMR (CD_2Cl_2, 600 MHz) of **8Ph5Si**

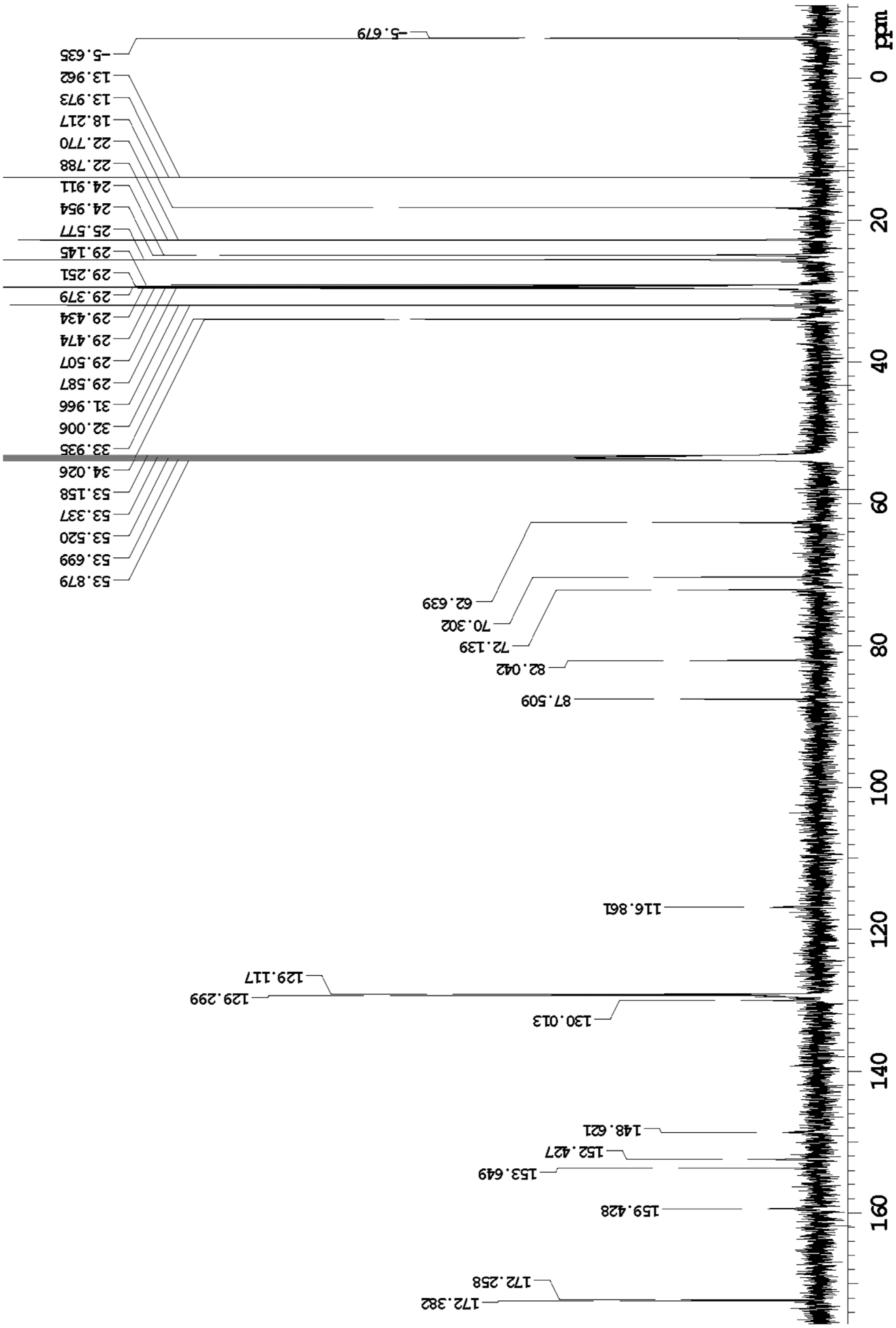

$^{13}C\{1H\}$NMR (CD_2Cl_2, 600 MHz) of **8Ph5Si**

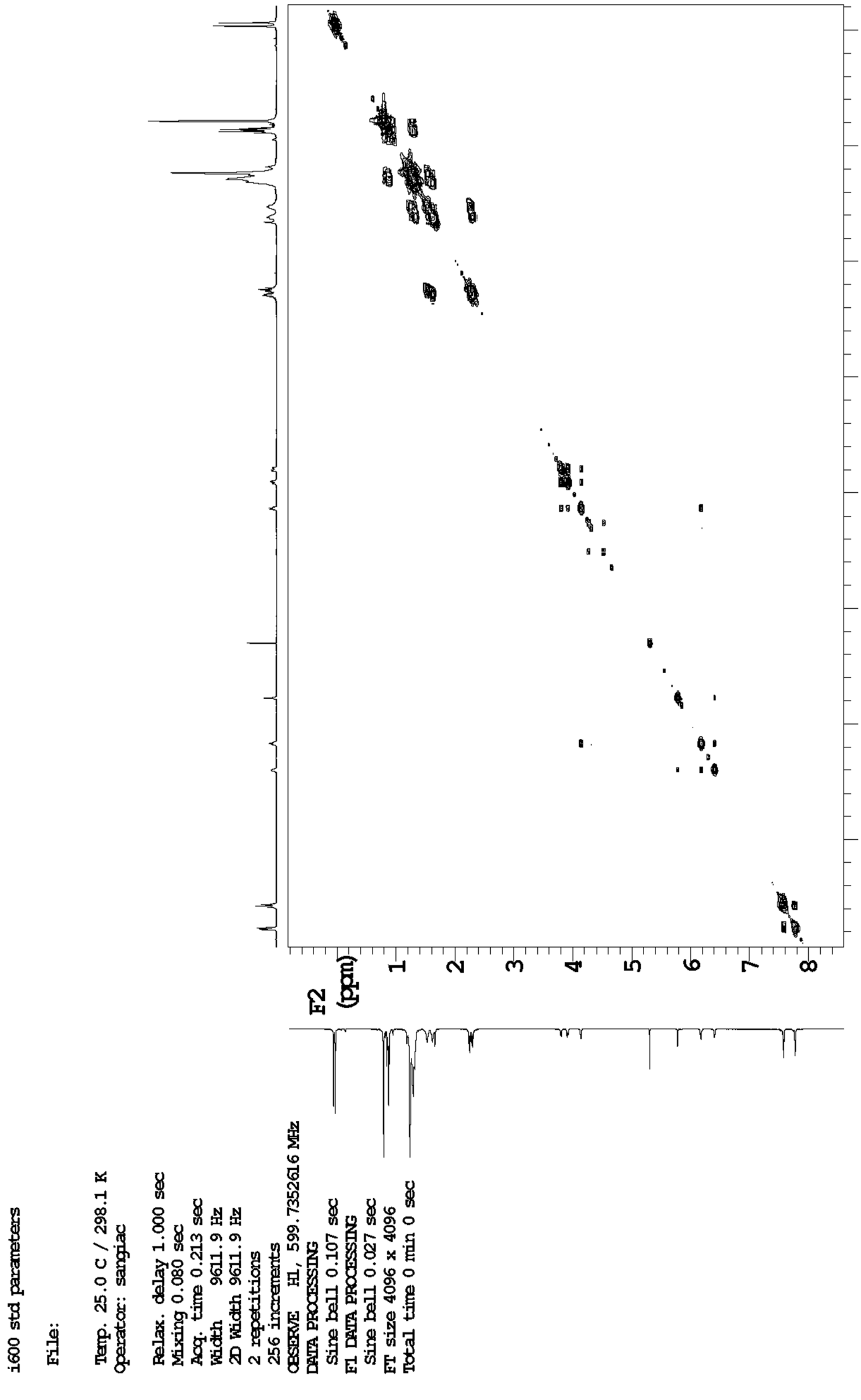

gCOSY spectrum (CD_2Cl_2, 600 MHz) of **8Ph5Si**

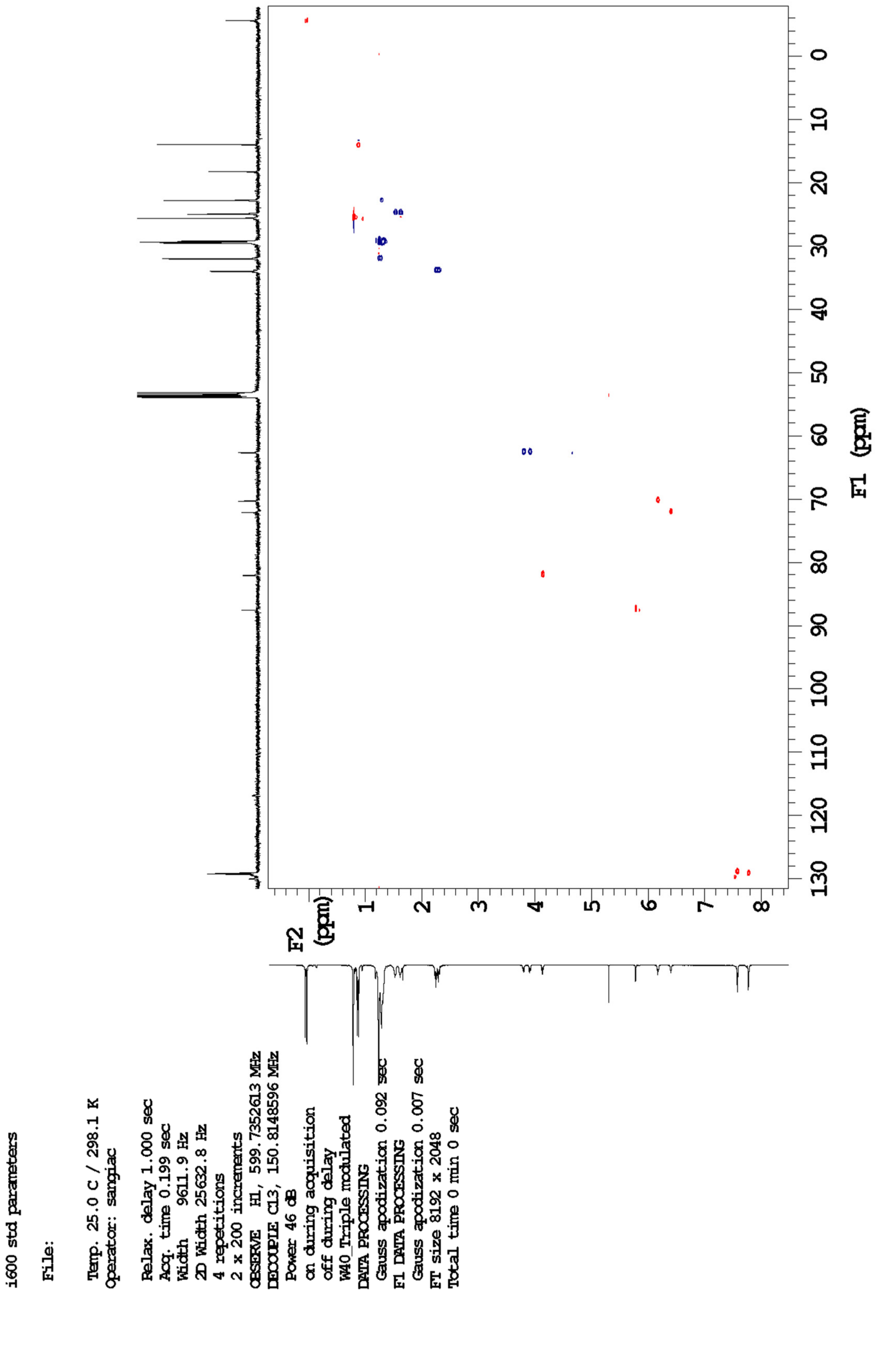

gHSQC spectrum (CD_2Cl_2, 600 MHz) of **8Ph5Si**

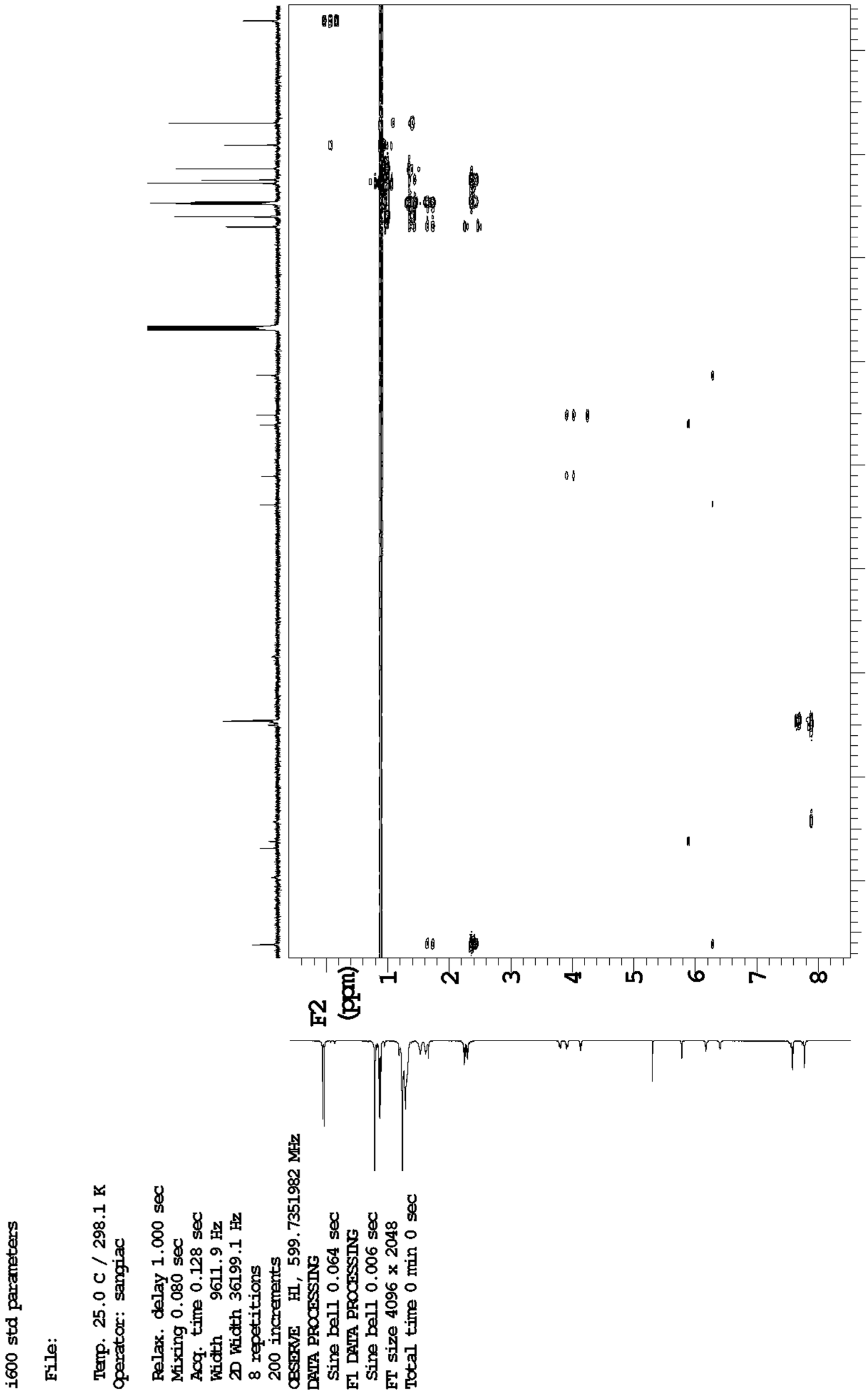

gHMBC spectrum (CD_2Cl_2, 600 MHz) of **8Ph5Si**

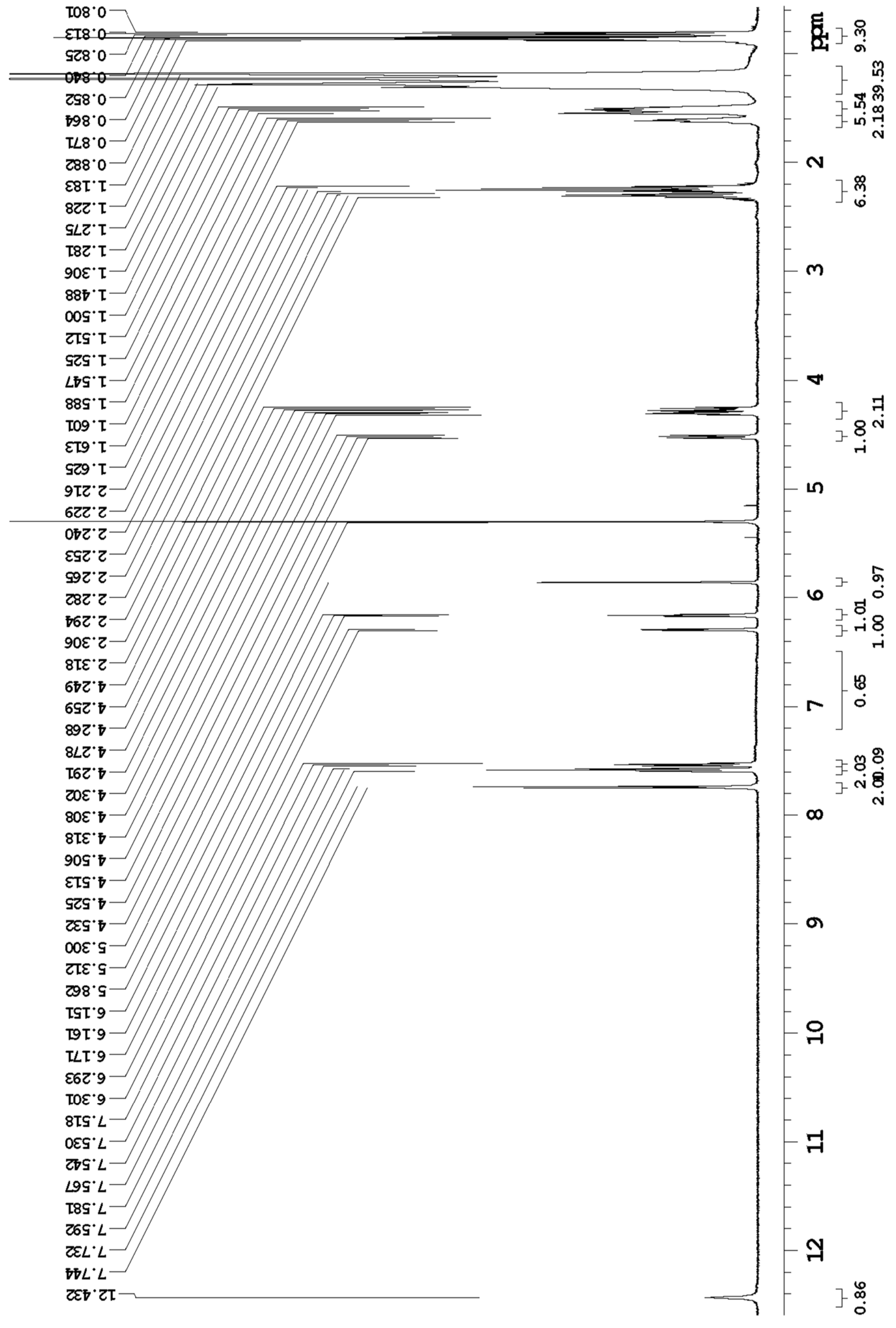

^{1}H-NMR (CD_2Cl_2, 600 MHz) of **8Ph5C10**

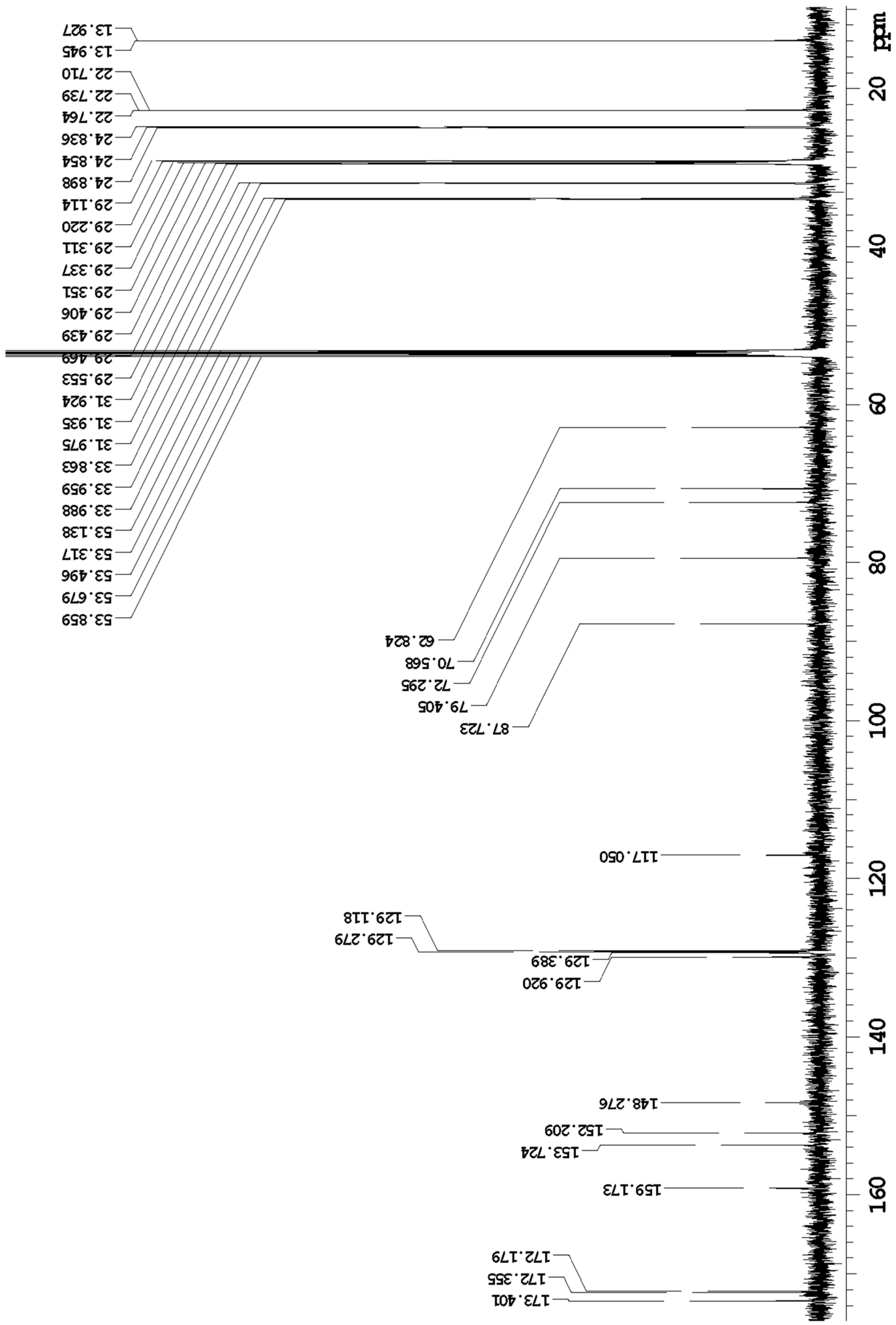

$^{13}C\{^{1}H\}$NMR (CD_2Cl_2, 600 MHz) of **8Ph5C10**

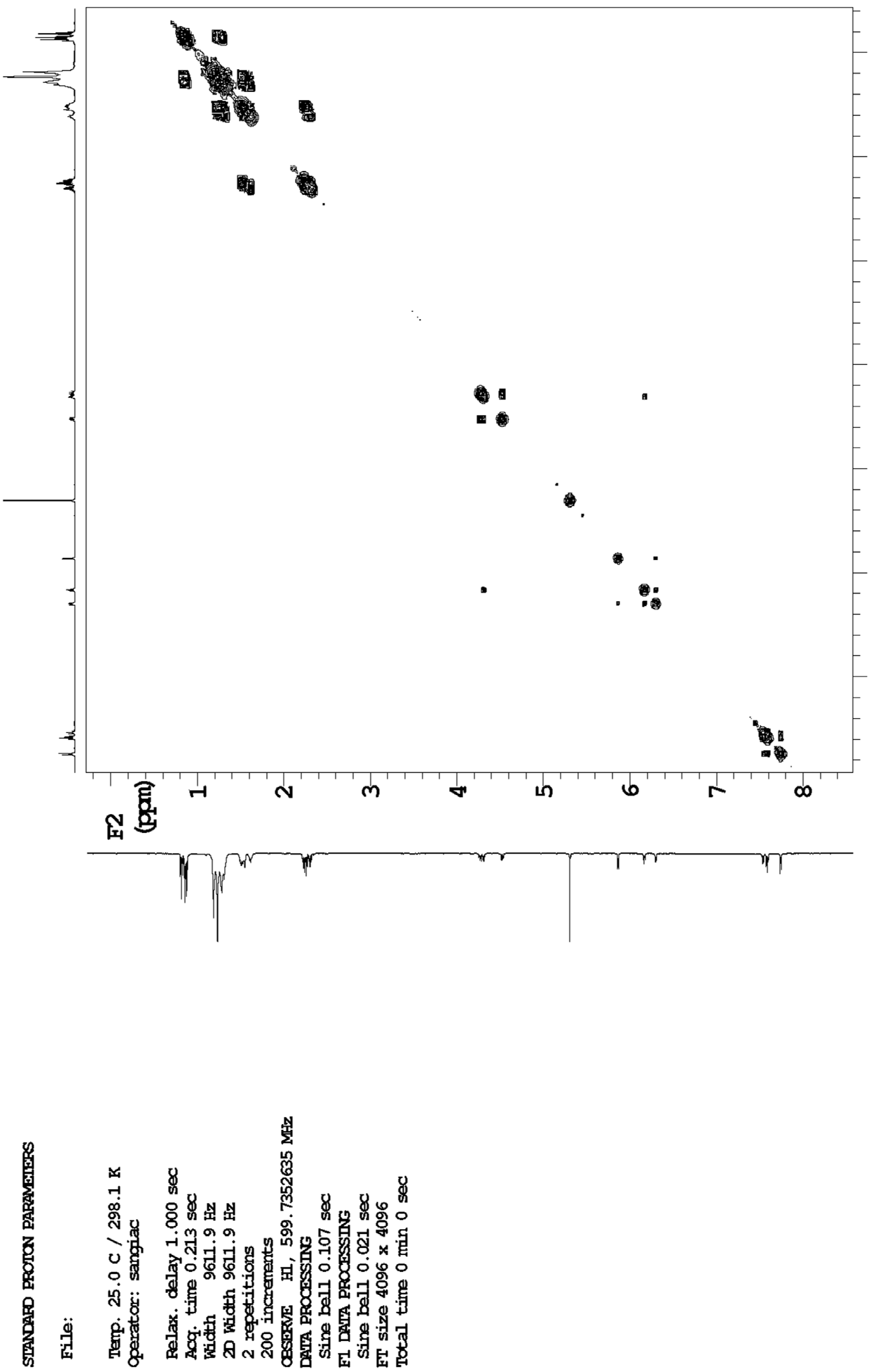

gCOSY spectrum (CD_2Cl_2, 600 MHz) of **8Ph5C10**

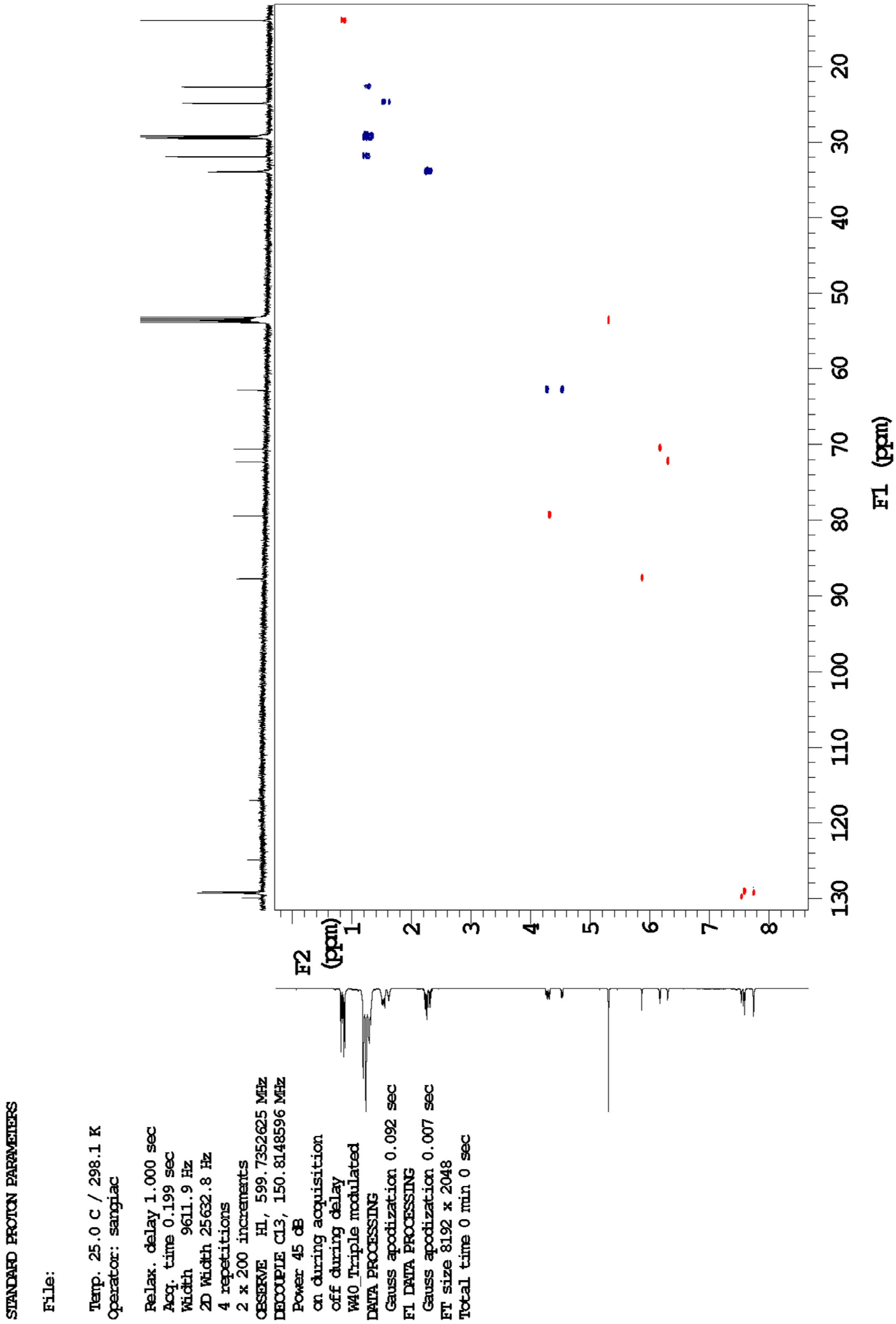

gHSQC spectrum (CD_2Cl_2, 600 MHz) of **8Ph5C10**

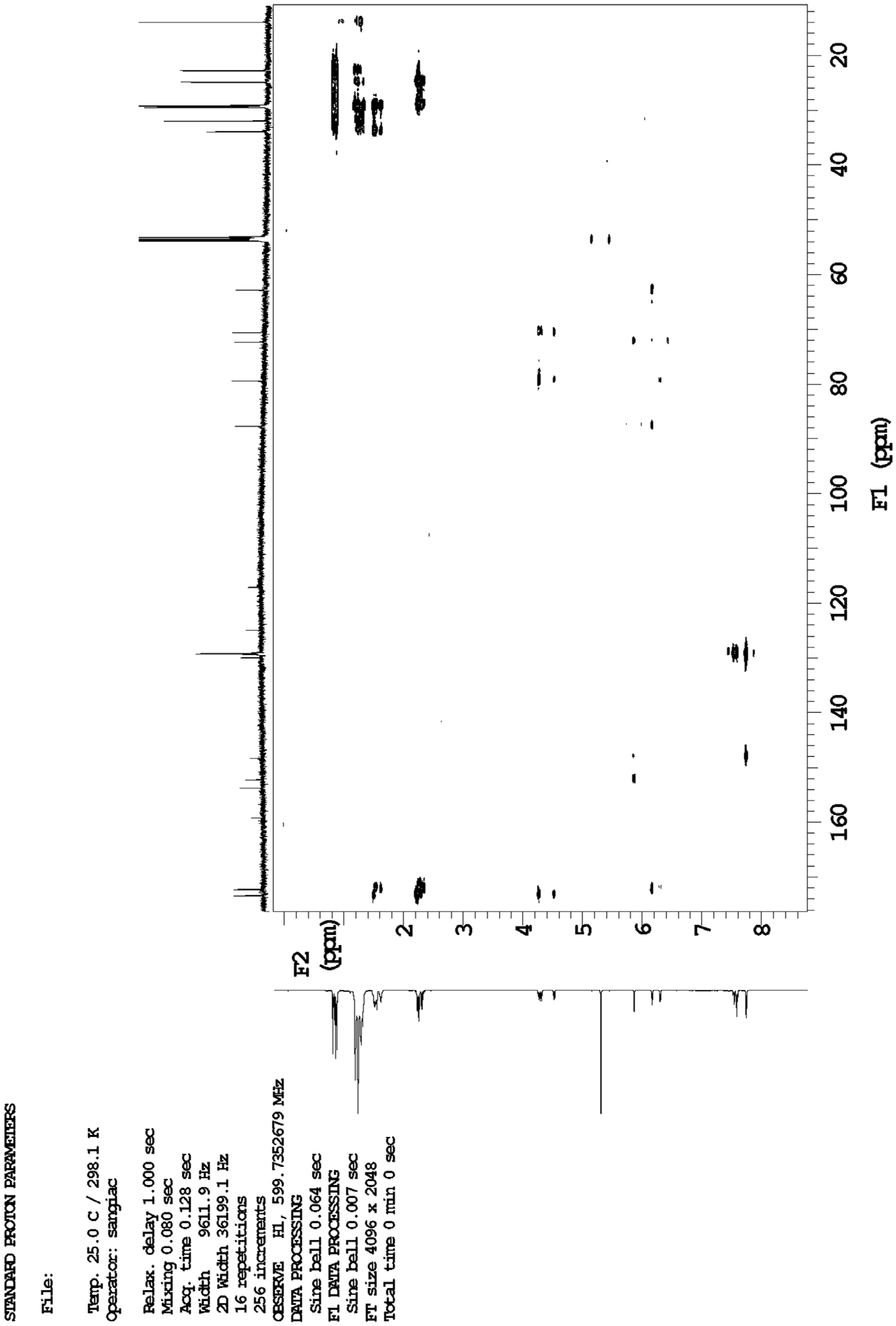

gHMBC spectrum (CD_2Cl_2, 600 MHz) of **8Ph5C10**

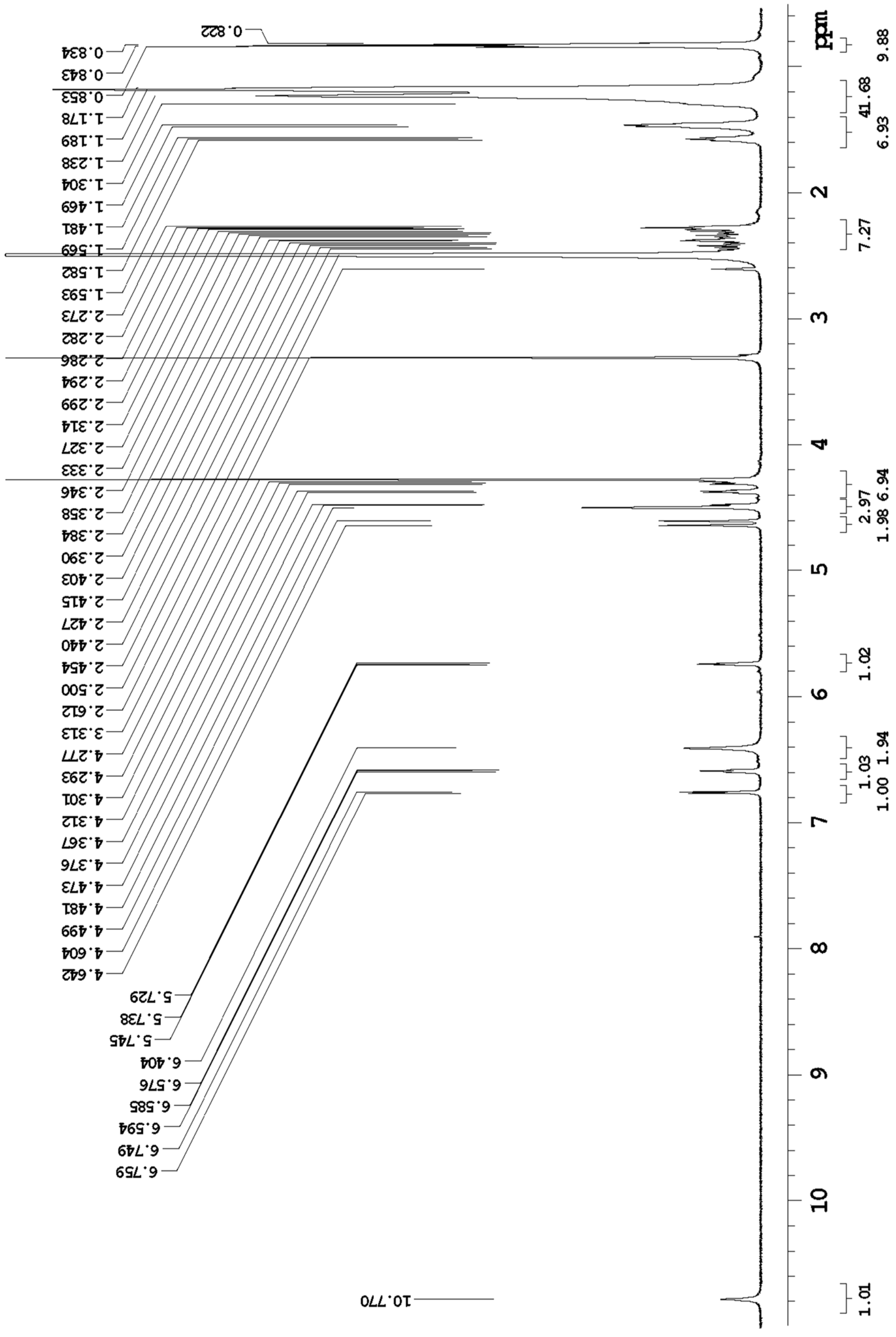

^{1}H-NMR (dmso-d$_6$, 600 MHz) of **8Fc5C10**

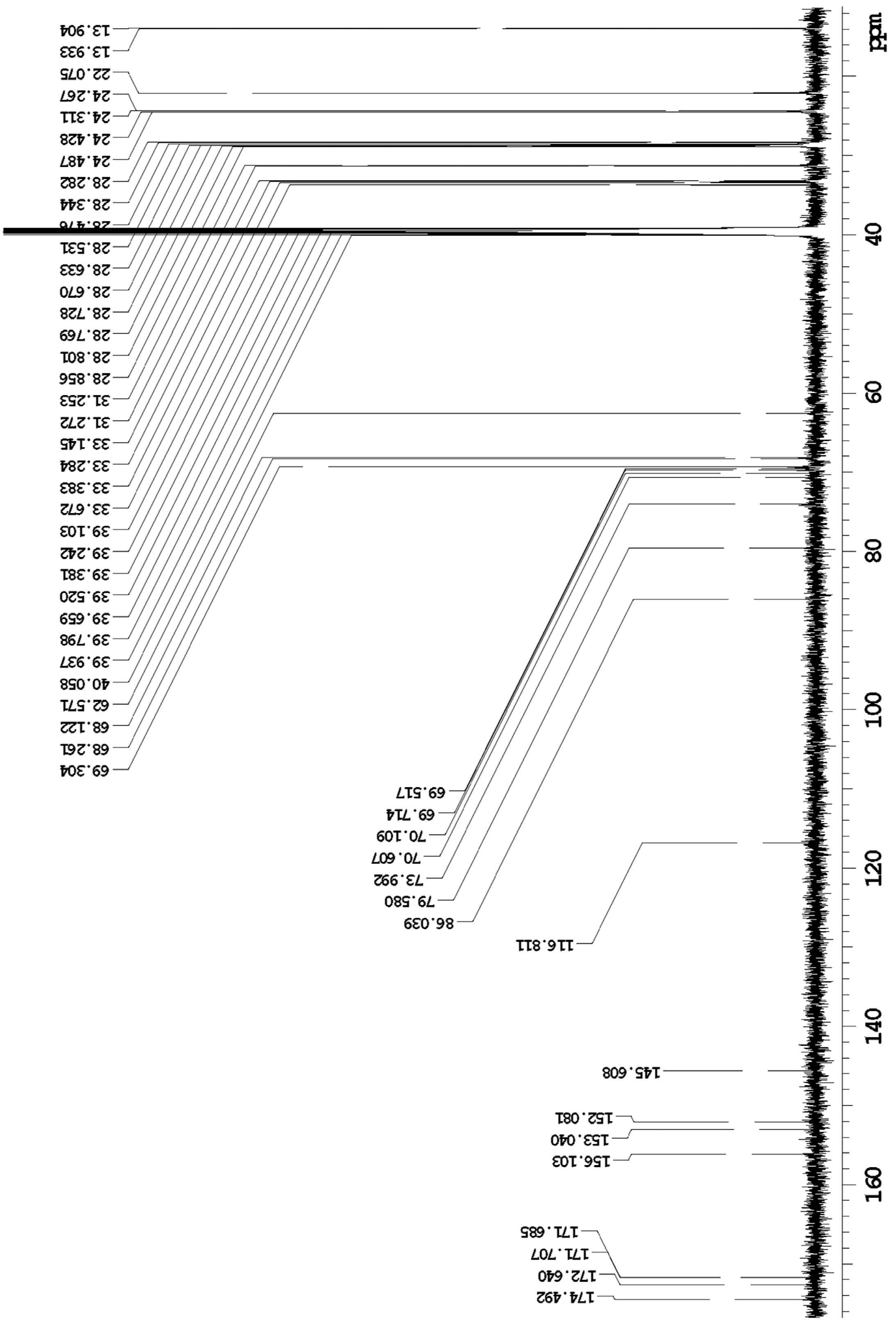

$^{13}C\{1H\}$NMR (dmso-d_6, 600 MHz) of **8Fc5C10**

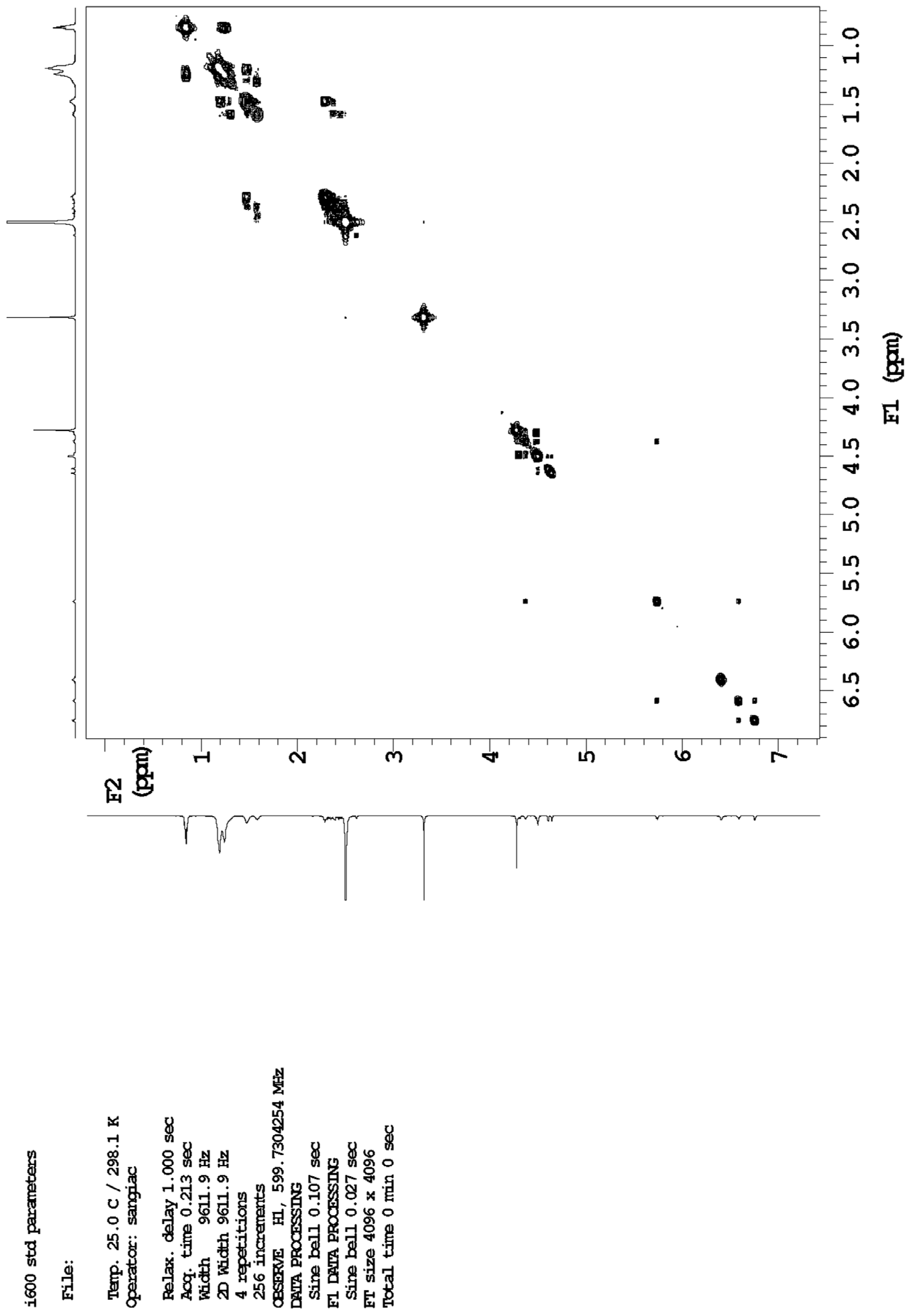

gCOSY spectrum (dmso-d_6, 600 MHz) of **8Fc5C10**

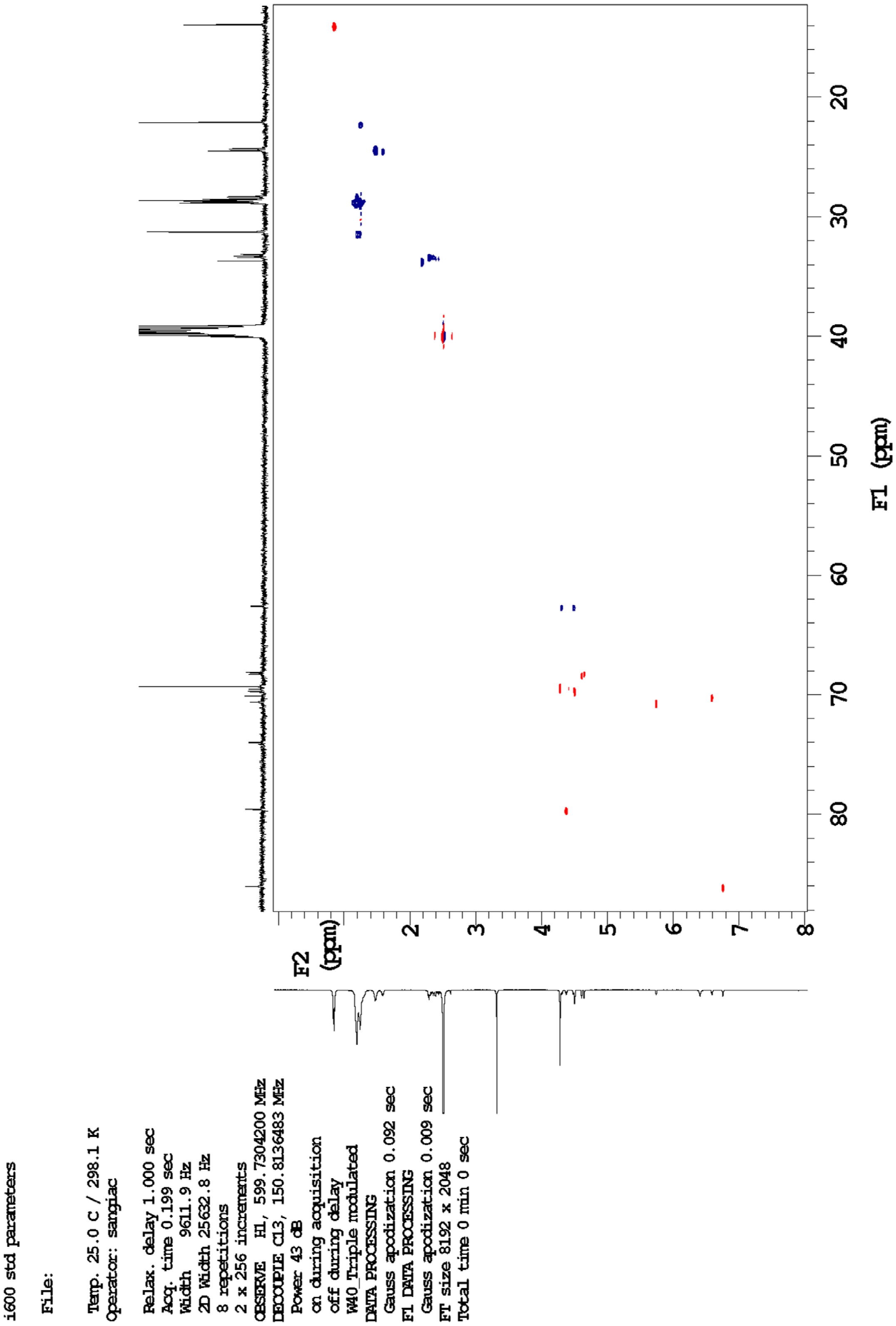

gHSQC spectrum (dmso-d6, 600 MHz) of **8Fc5C10**

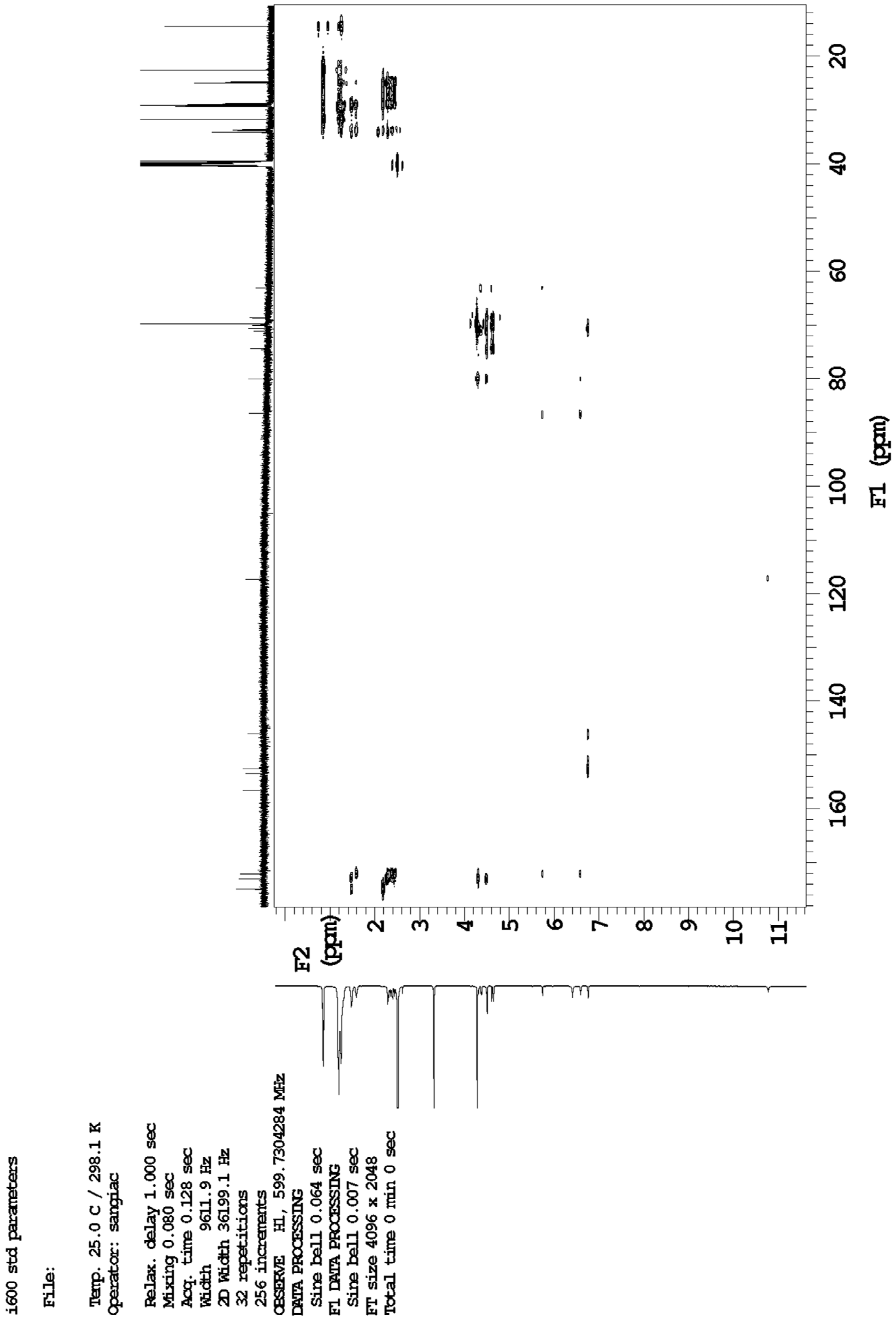

gHMBC spectrum (dmso-d$_6$, 600 MHz) of **8Fc5C10**

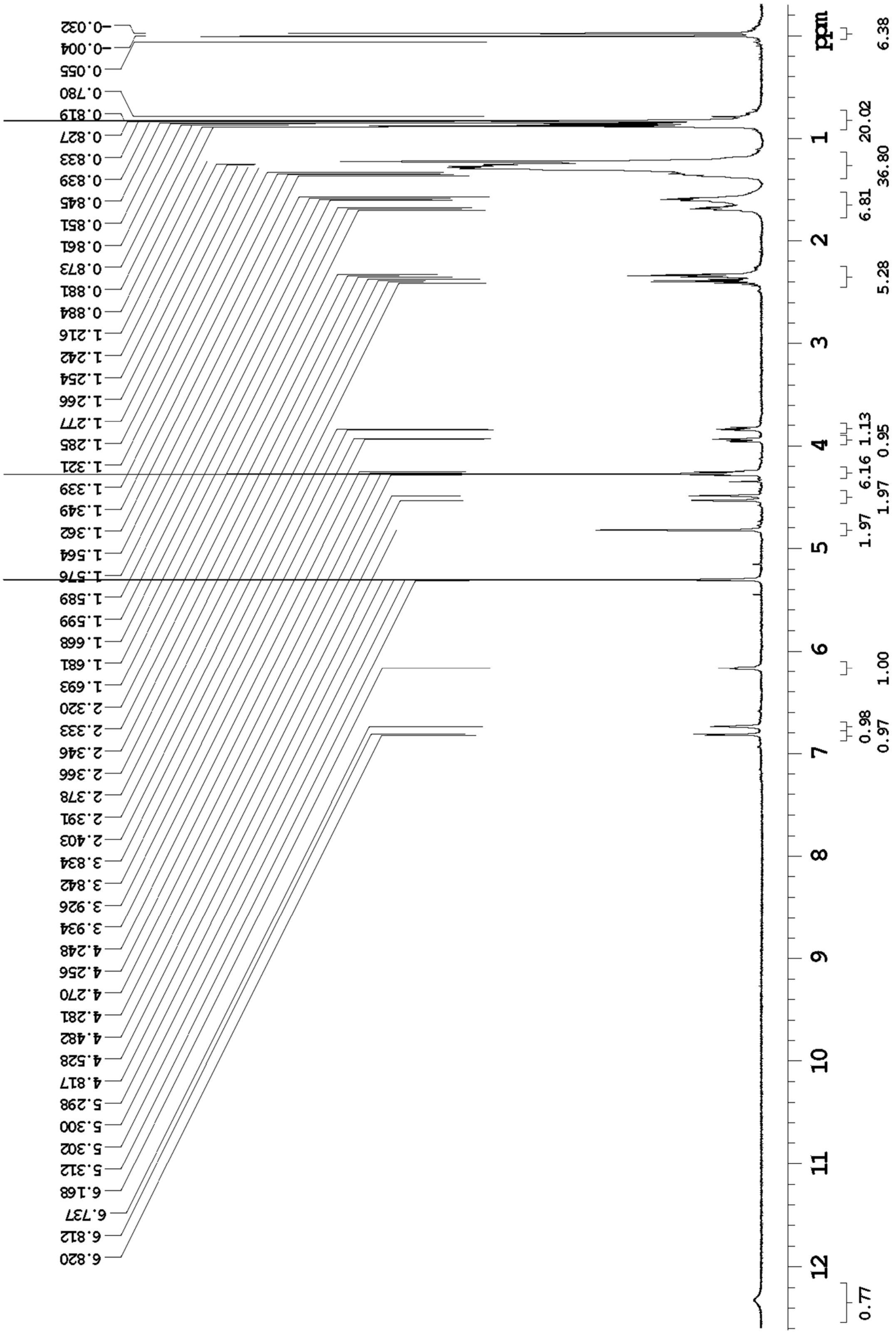

^{1}H-NMR (CD_2Cl_2, 600 MHz) of **8Fc5Si**

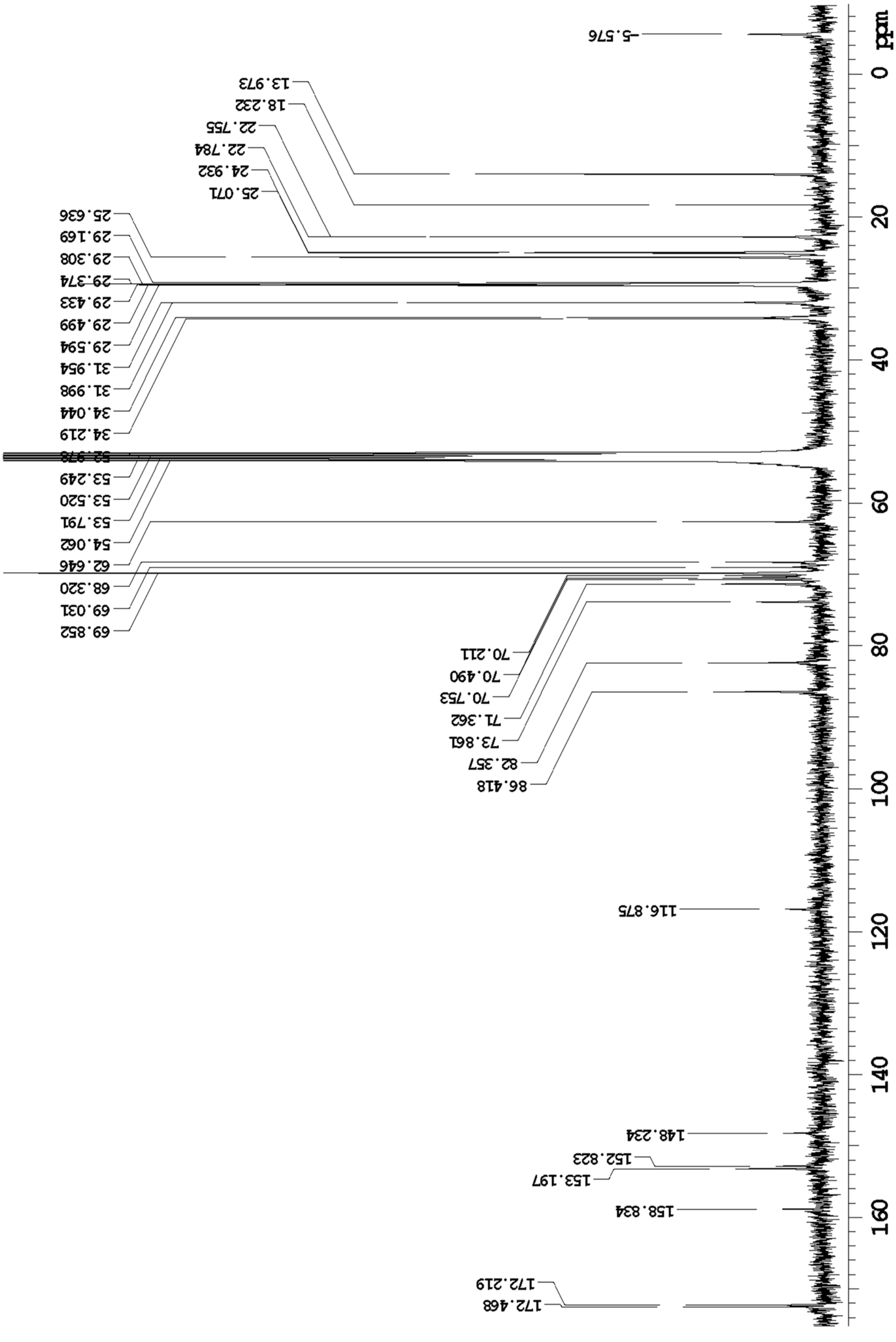

$^{13}C\{1H\}$NMR (CD_2Cl_2, 600 MHz) of **8Fc5Si**

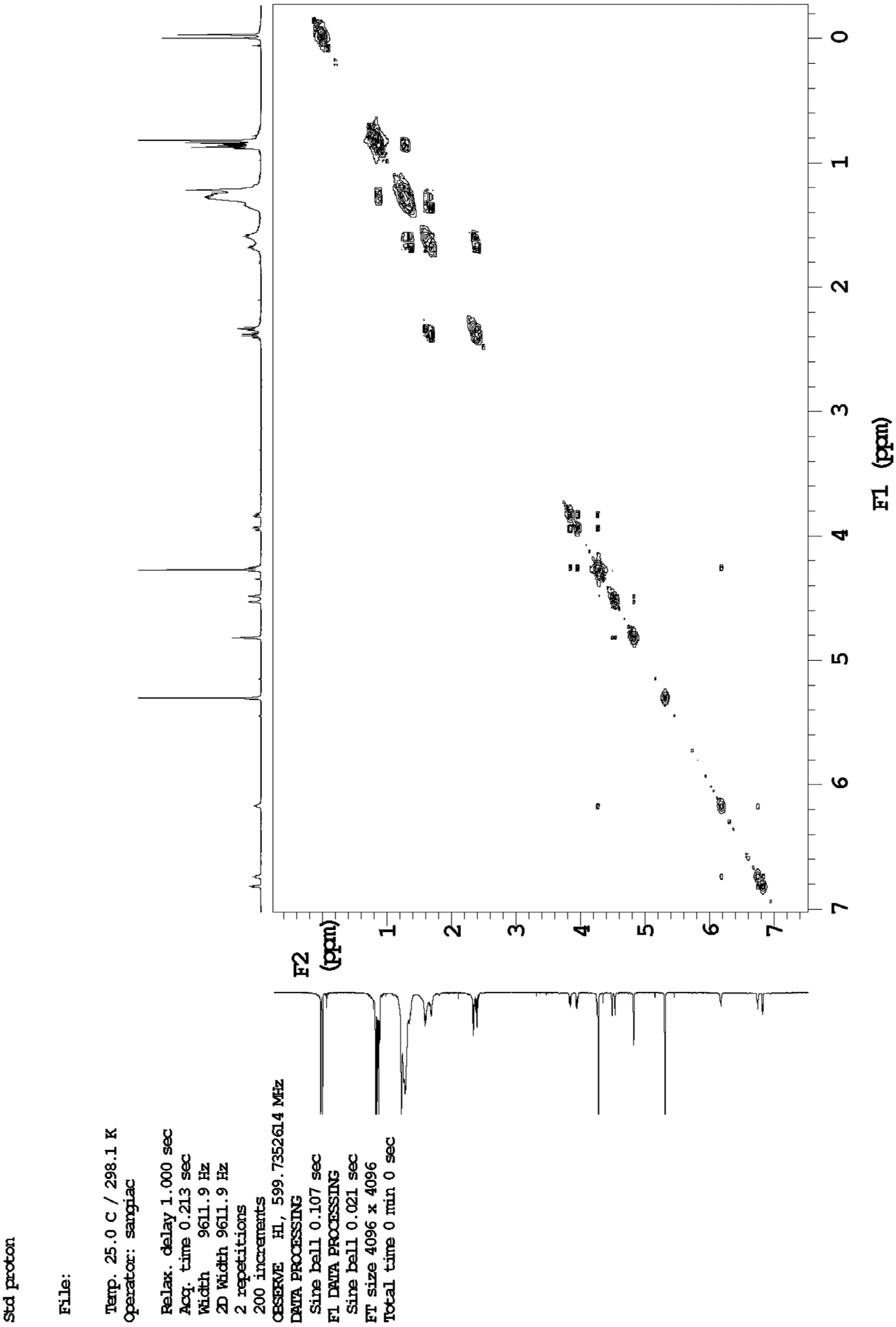

gCOSY spectrum (CD_2Cl_2, 600 MHz) of **8Fc5Si**

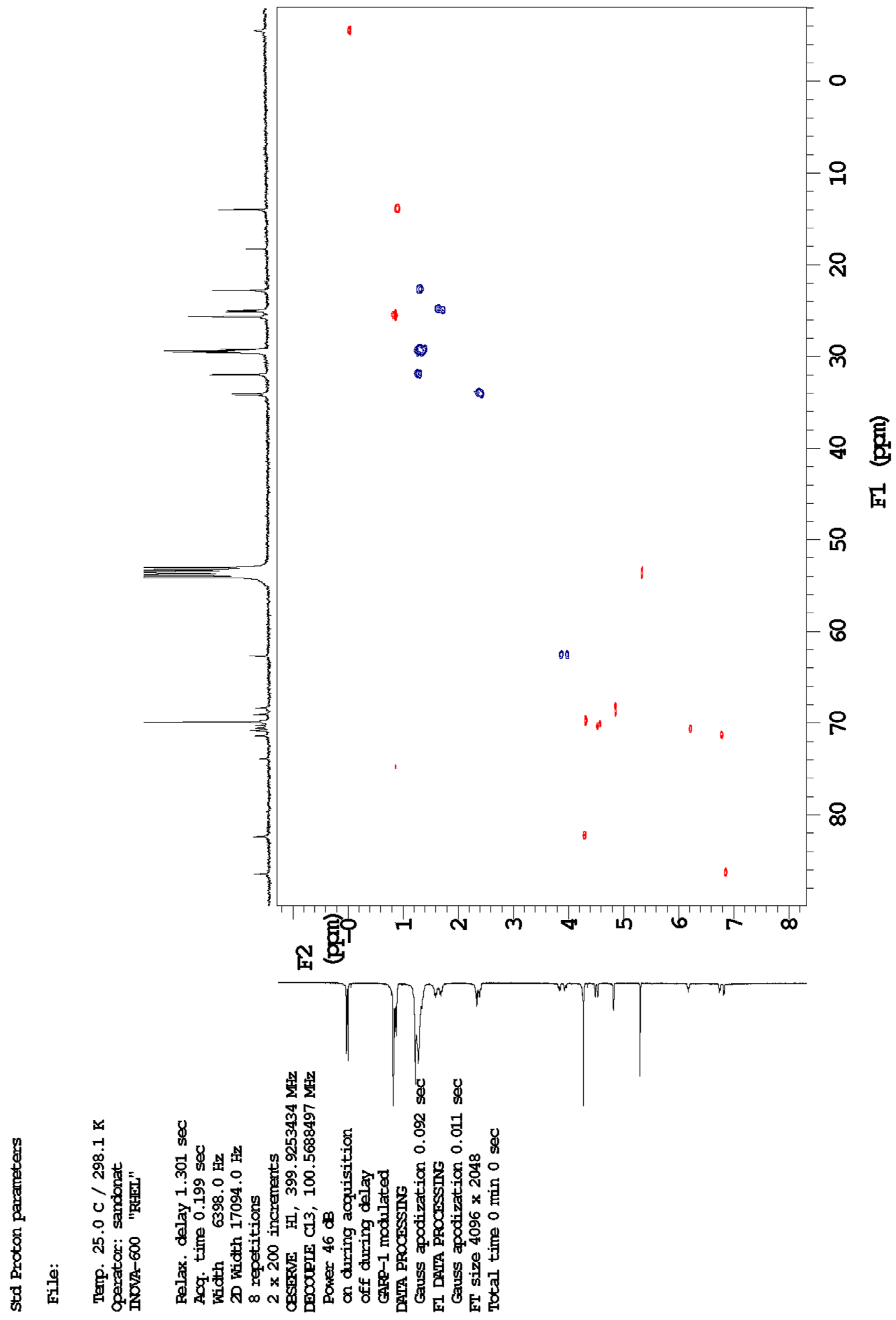

gHSQC spectrum (CD_2Cl_2, 600 MHz) of **8Fc5Si**

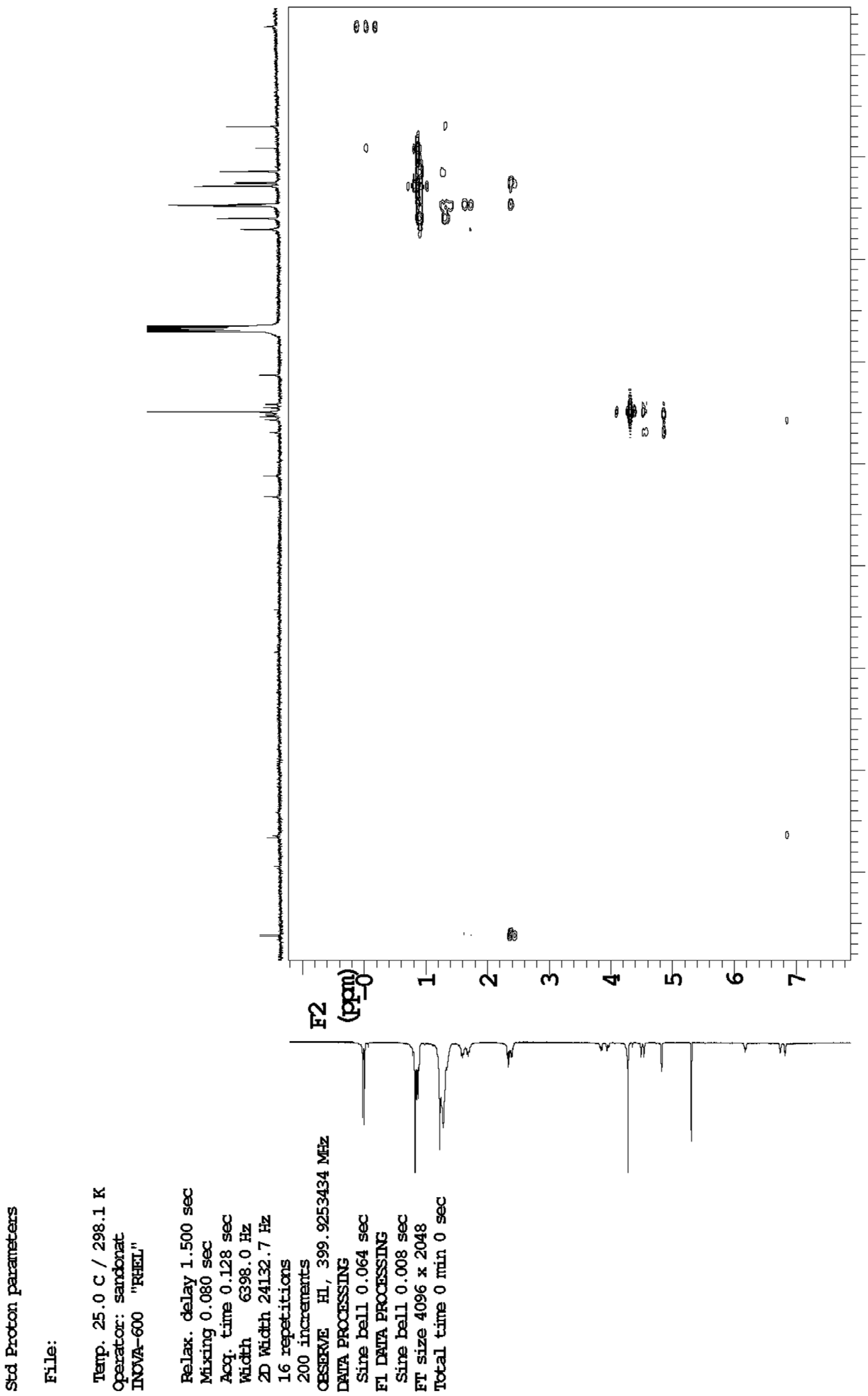

gHMBC spectrum (CD_2Cl_2, 600 MHz) of **8Fc5Si**

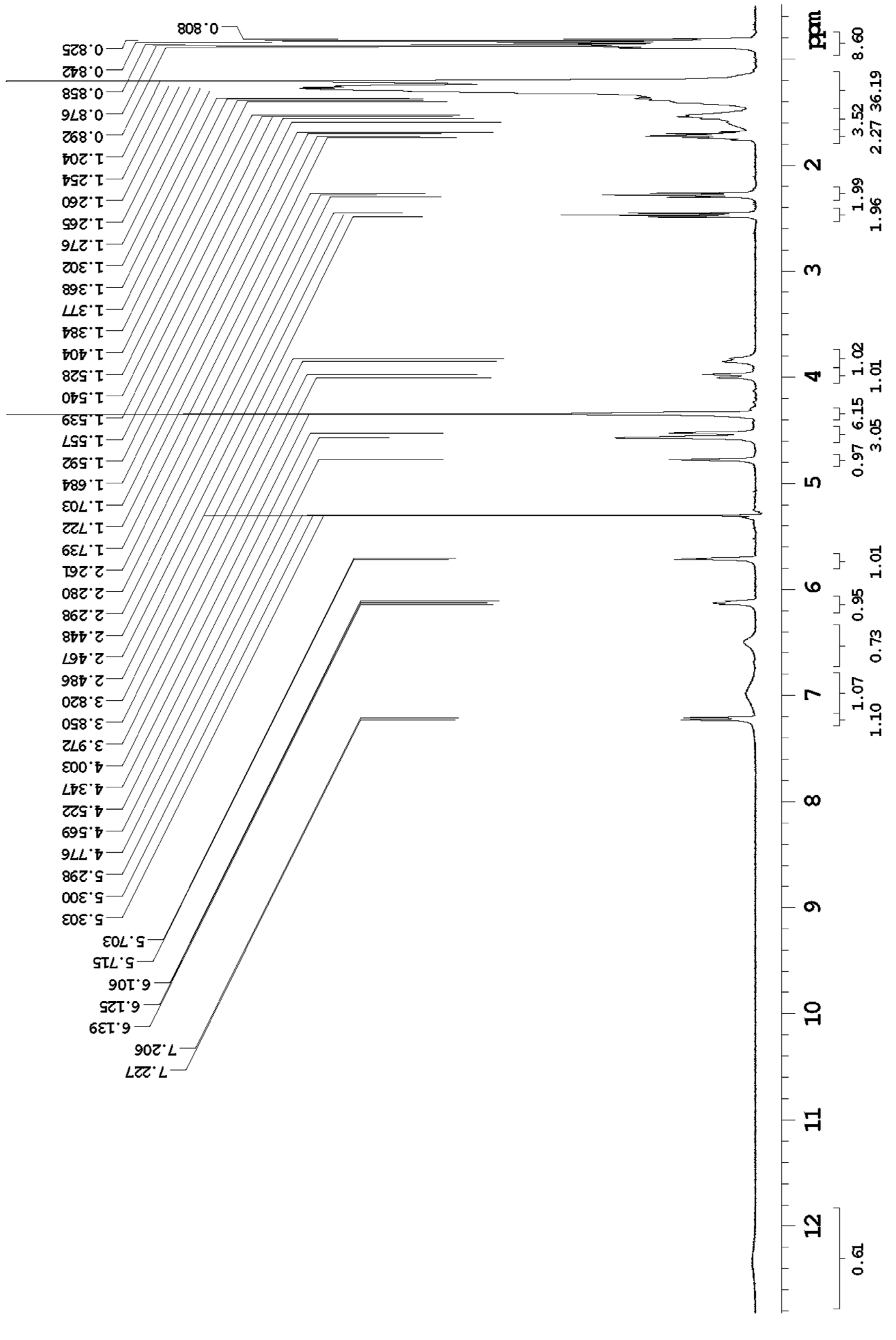

^{1}H-NMR (CD_2Cl_2, 600 MHz) of **8Fc5OH**

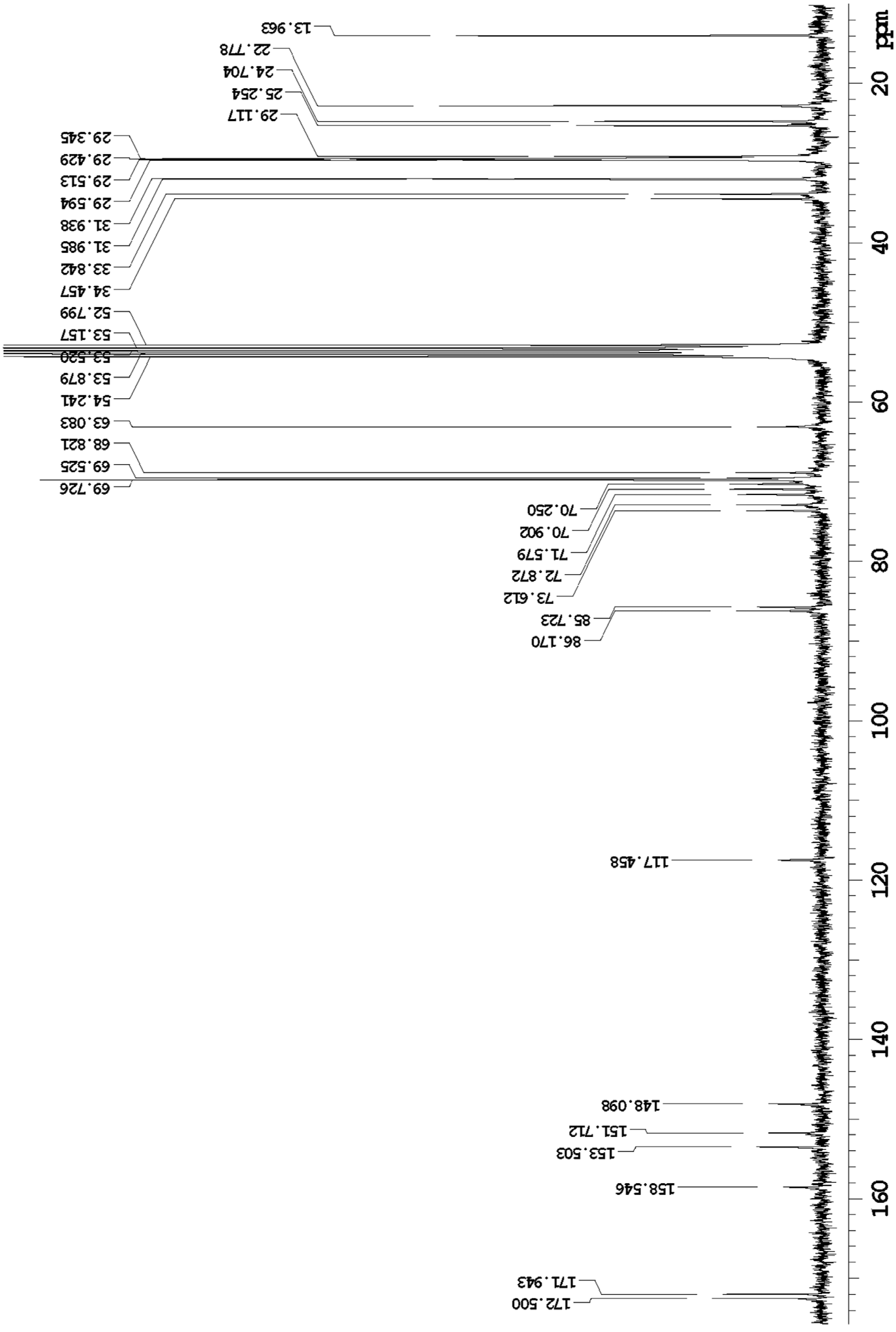

$^{13}C\{^1H\}$NMR (CD_2Cl_2, 600 MHz) of **8Fc5OH**.

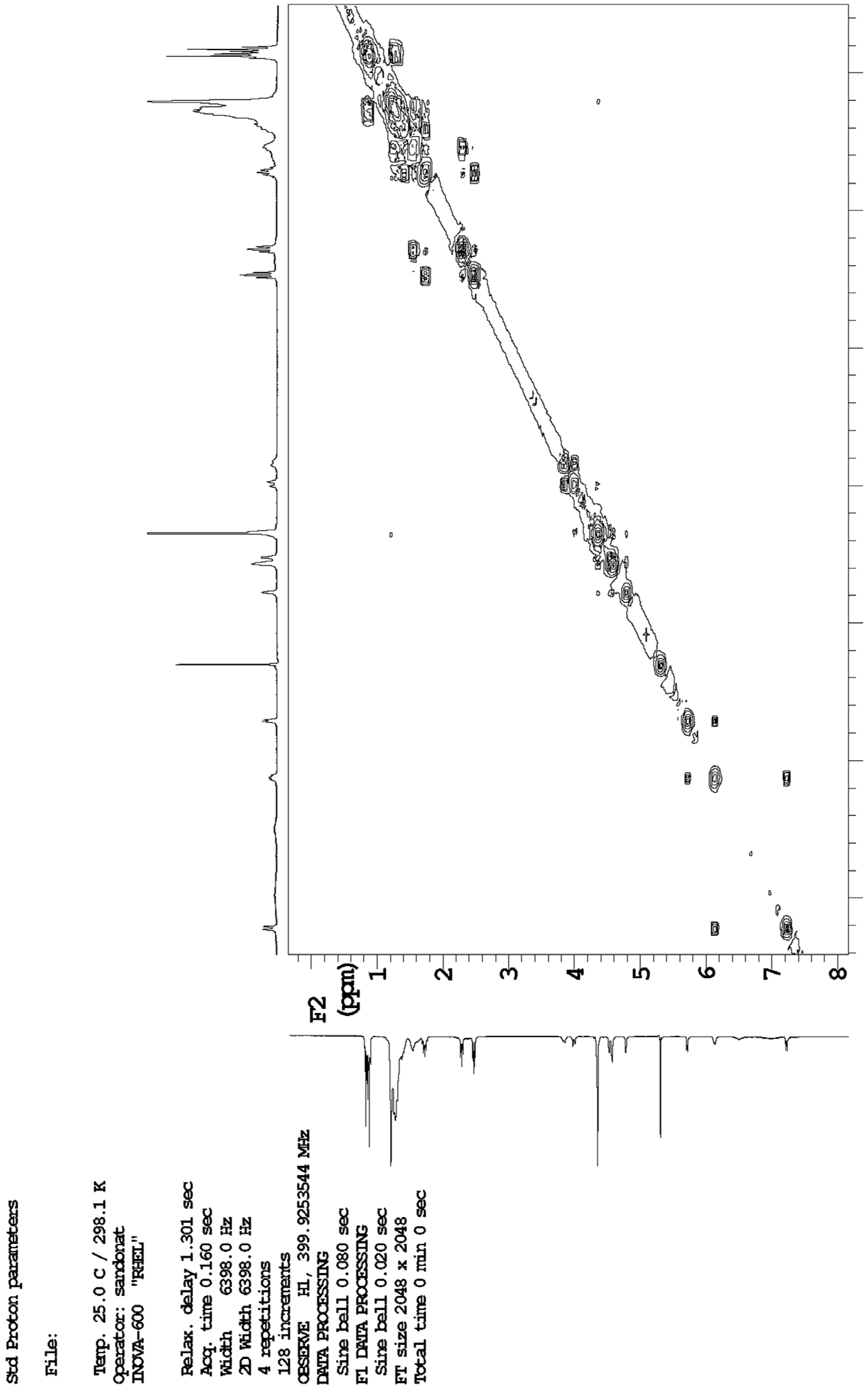

gCOSY spectrum (CD_2Cl_2, 600 MHz) of **8Fc5OH**

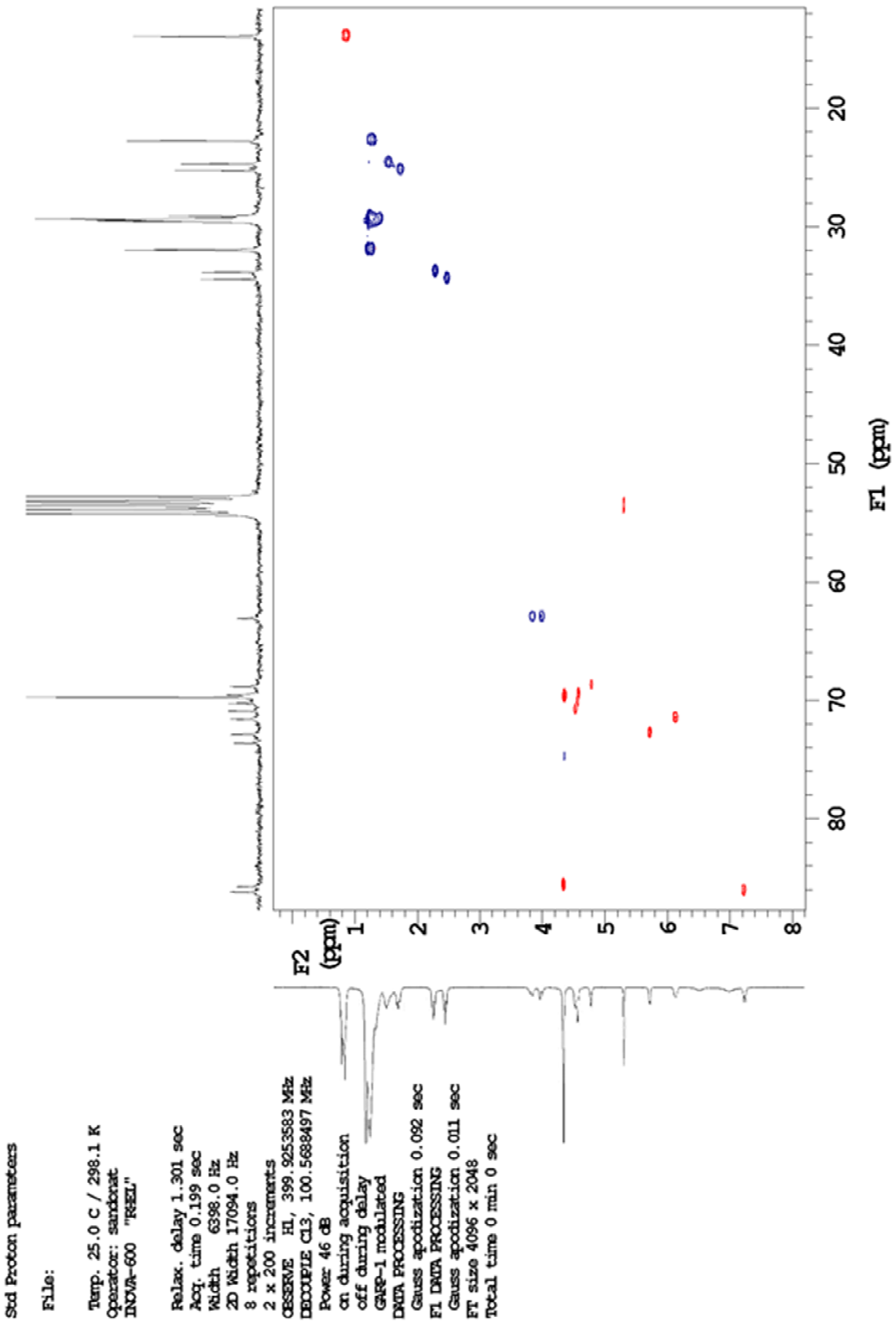

gHSQC spectrum (CD_2Cl_2, 600 MHz) of **8Fc5OH**

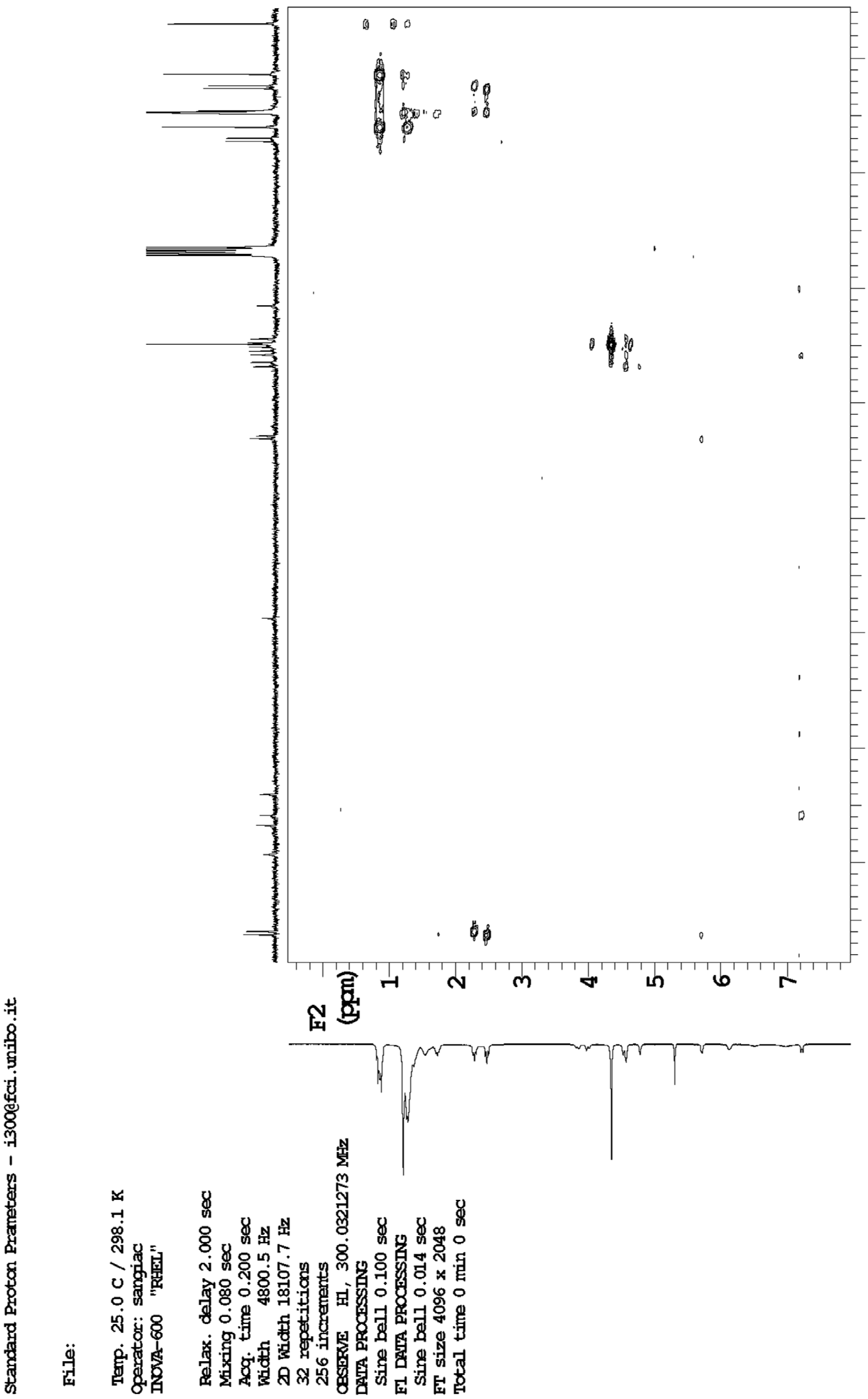

gHMBC spectrum (CD_2Cl_2, 600 MHz) of **8Fc5OH**

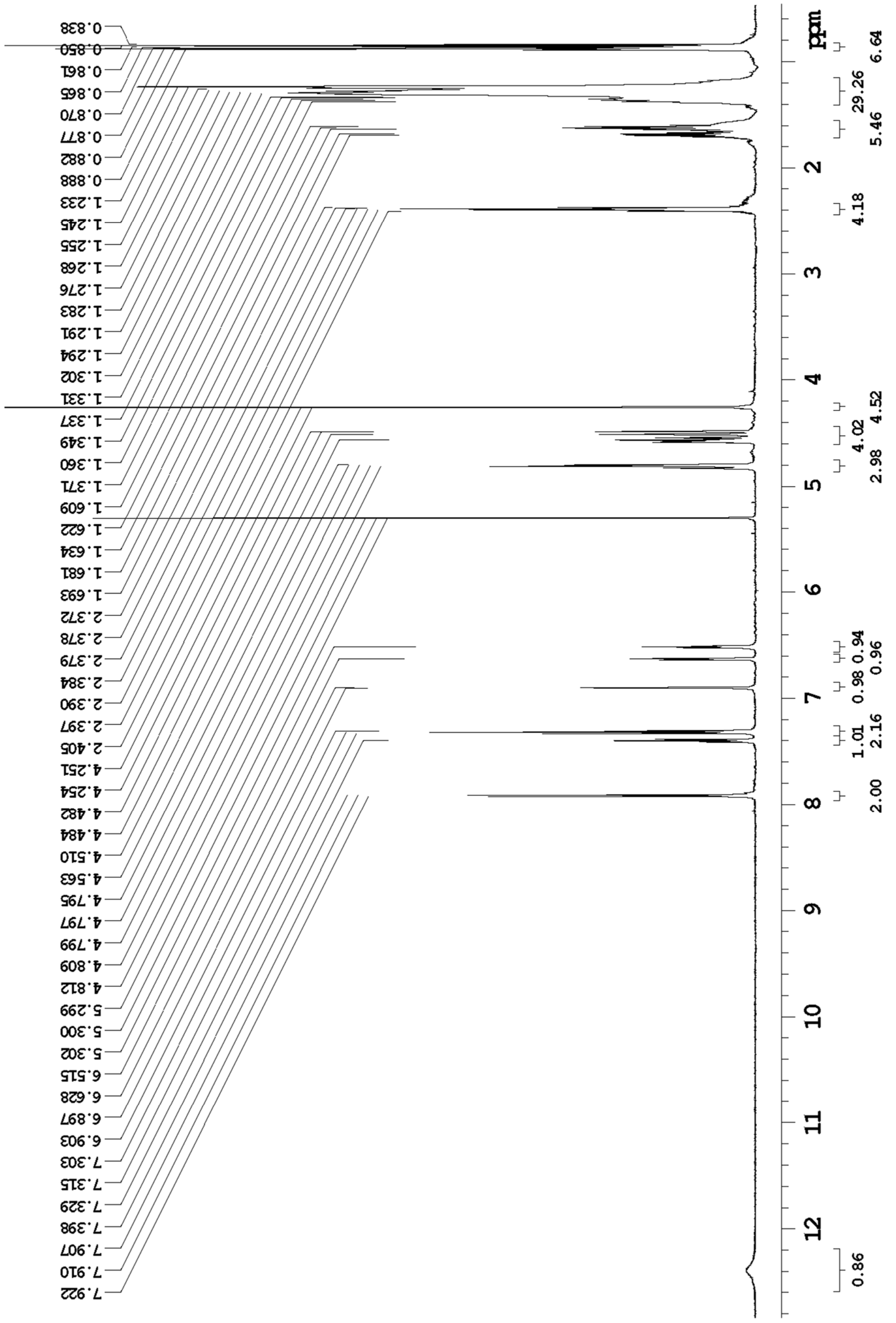

^{1}H-NMR (CD_2Cl_2, 600 MHz) of **8Fc5Ph**

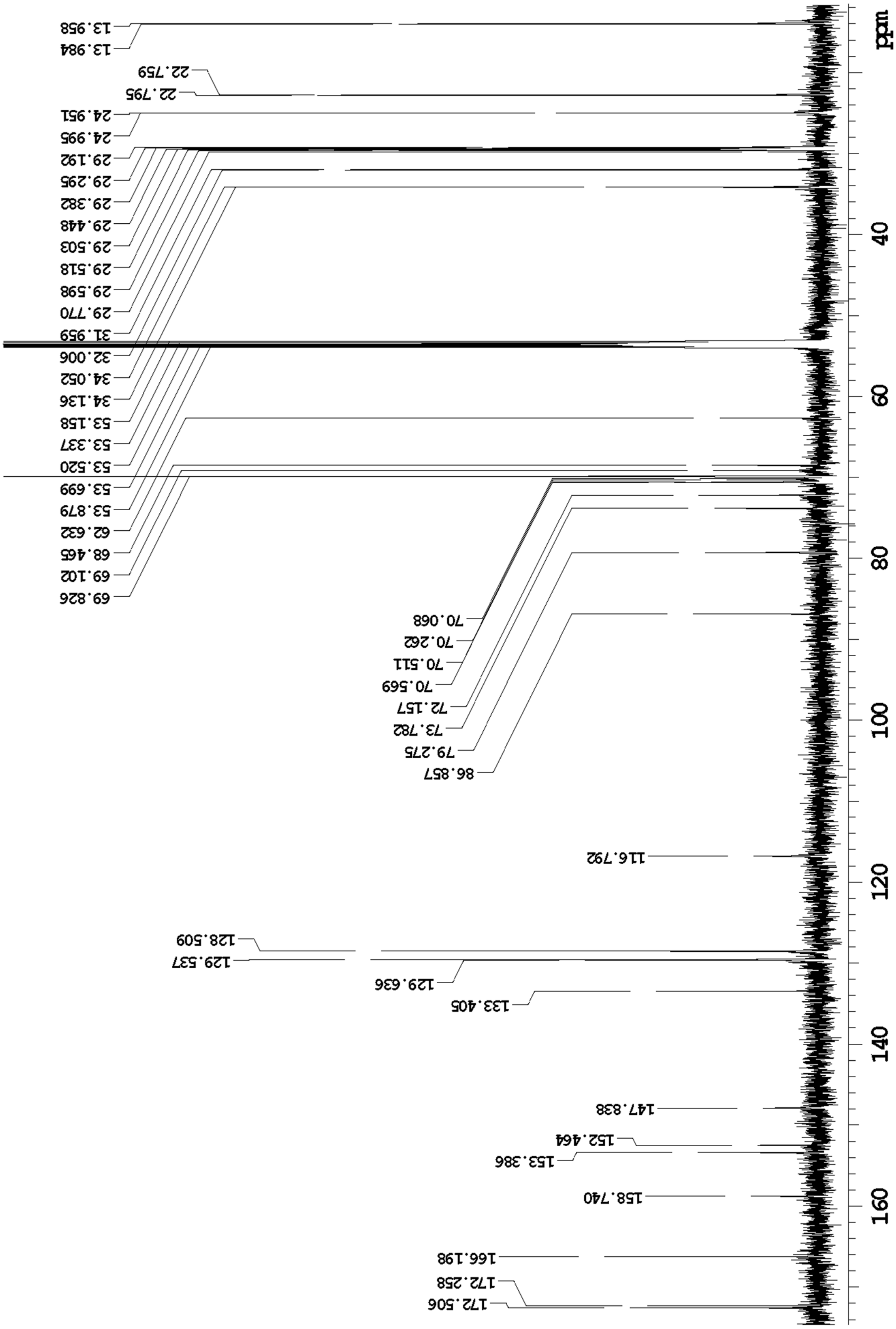

$^{13}C\{1H\}$NMR (CD_2Cl_2, 600 MHz) of **8Fc5Ph**.

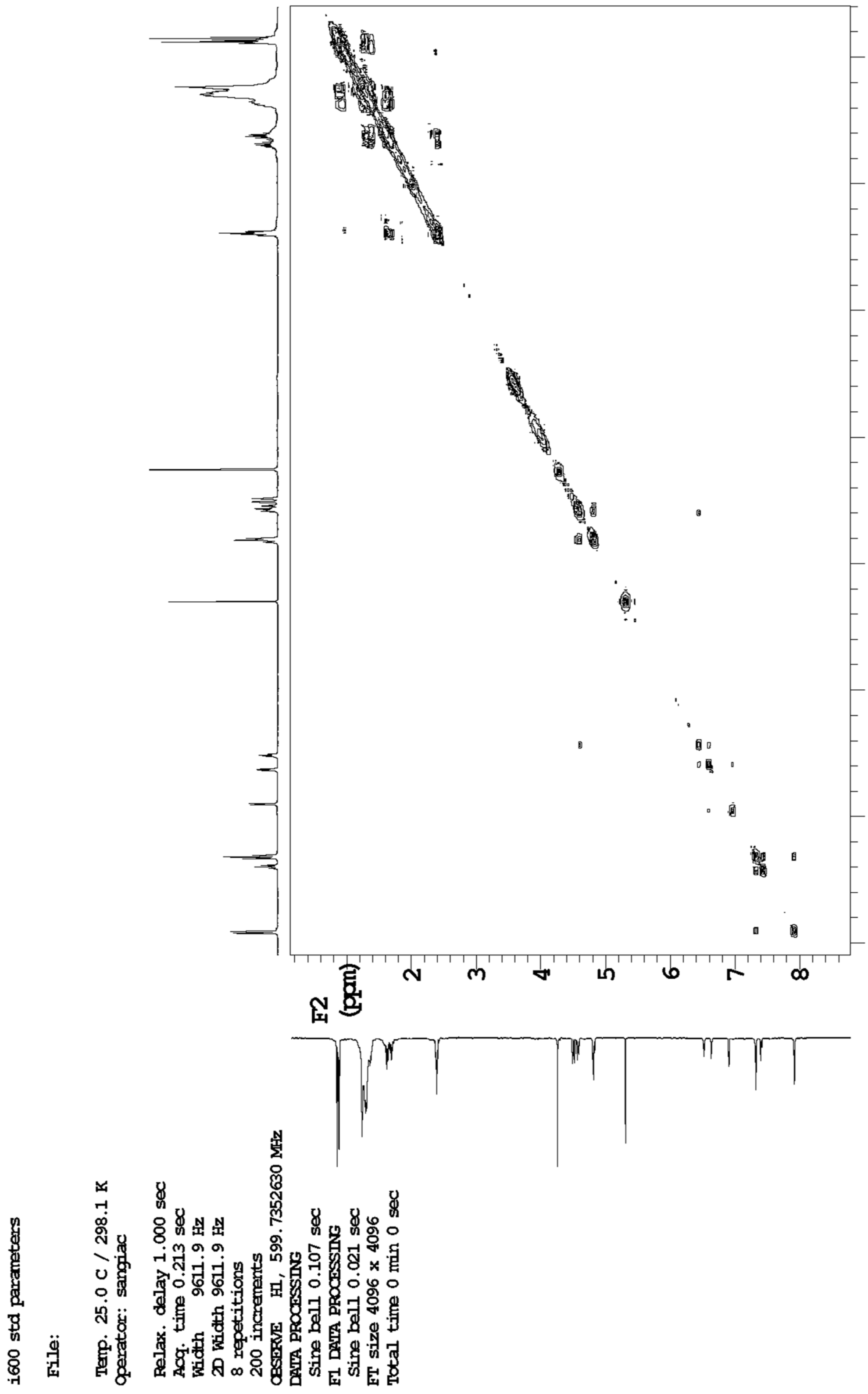

gCOSY spectrum (CD_2Cl_2, 600 MHz) of **8Fc5Ph**

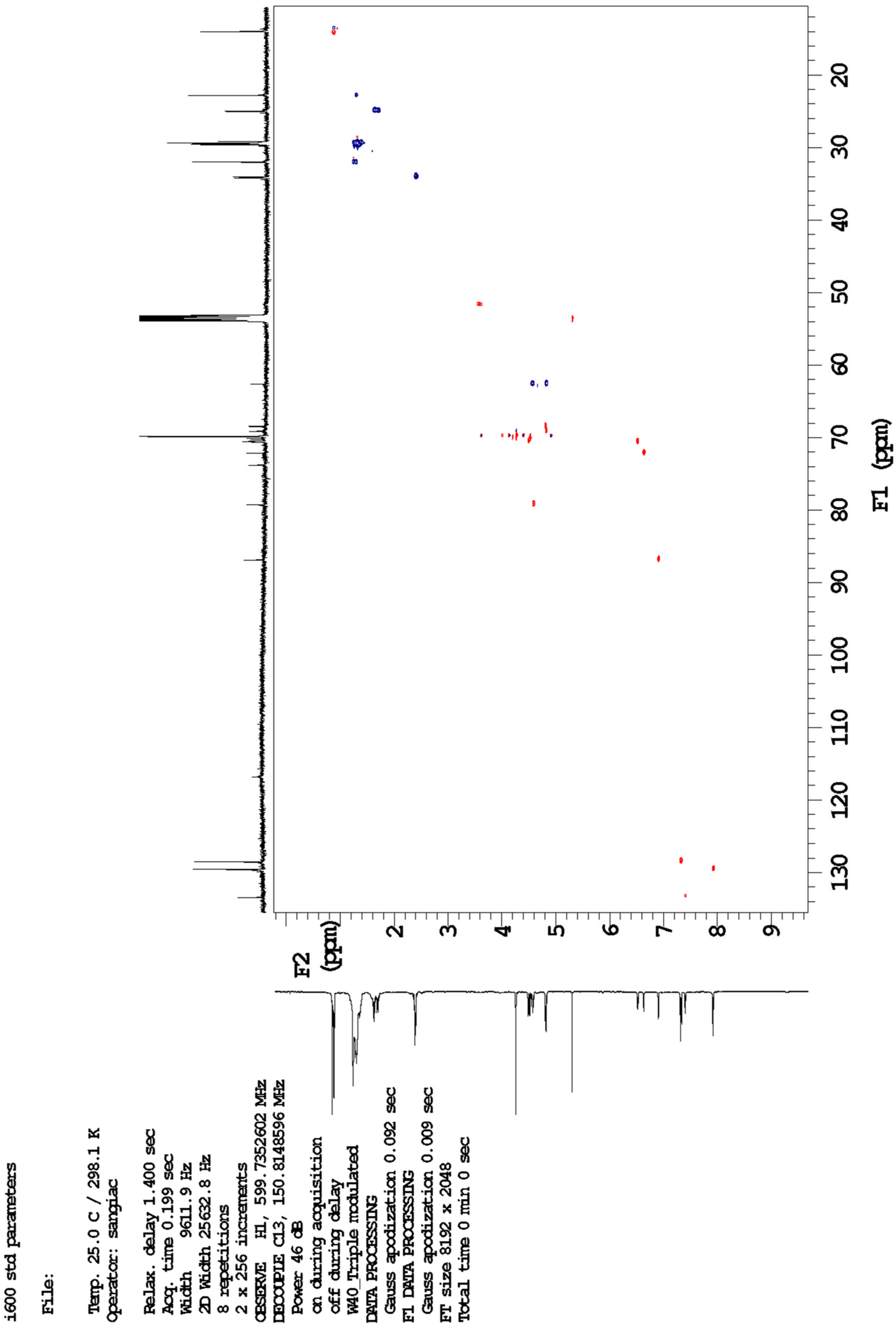

gHSQC spectrum (CD_2Cl_2, 600 MHz) of **8Fc5Ph**

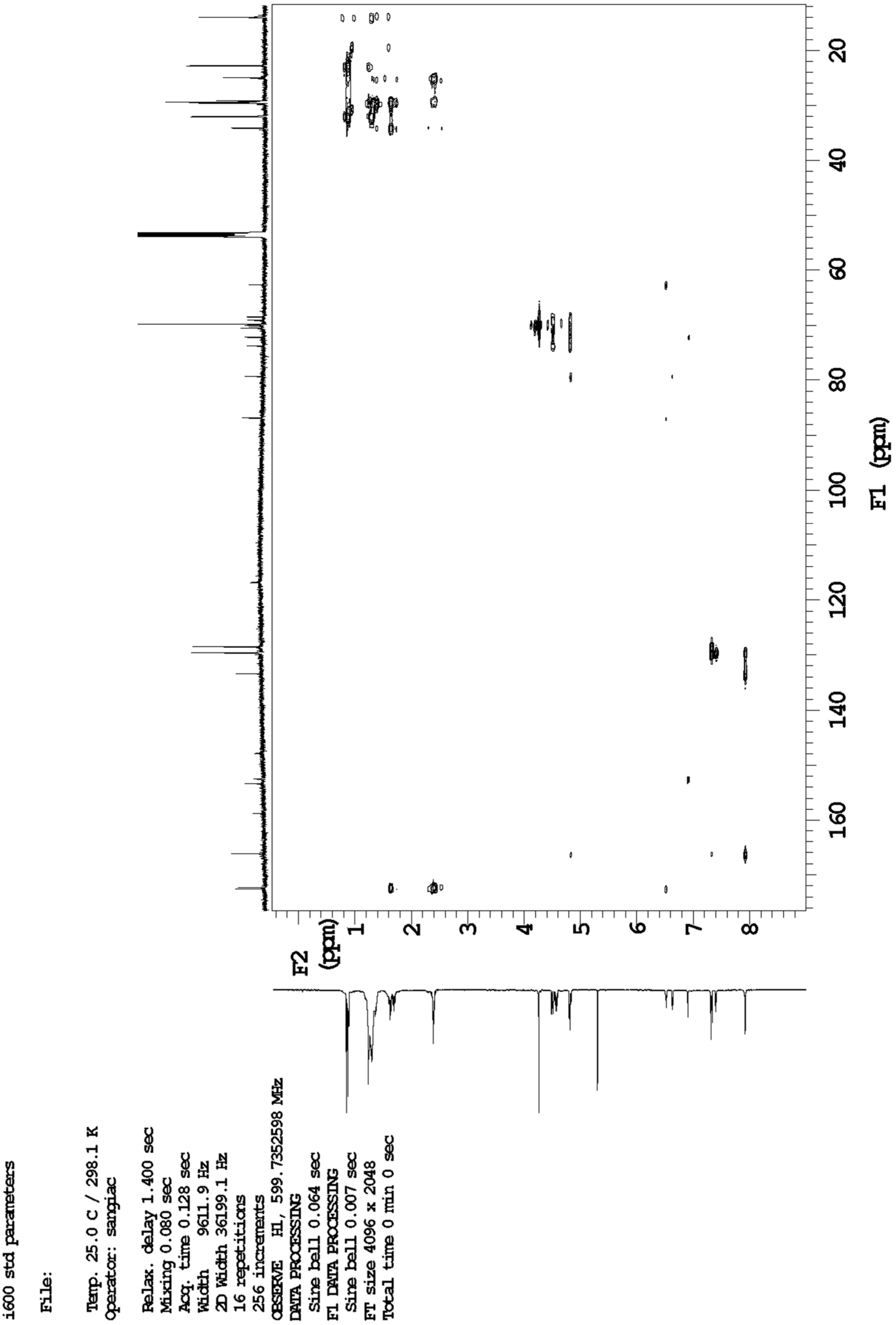

gHMBC spectrum (CD_2Cl_2, 600 MHz) of **8Fc5P**

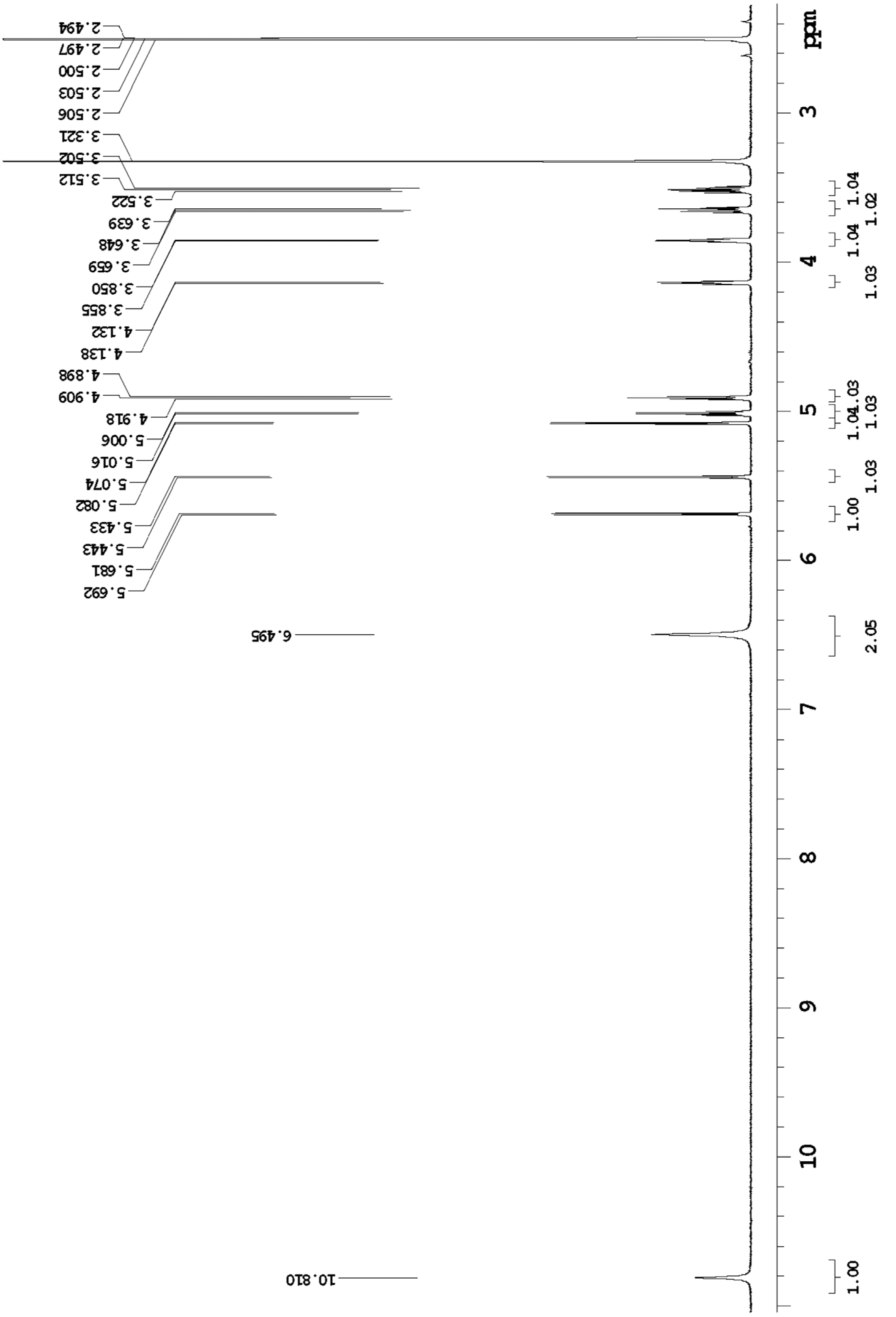

^{1}H-NMR (dmso-d_6, 600 MHz) of **9**

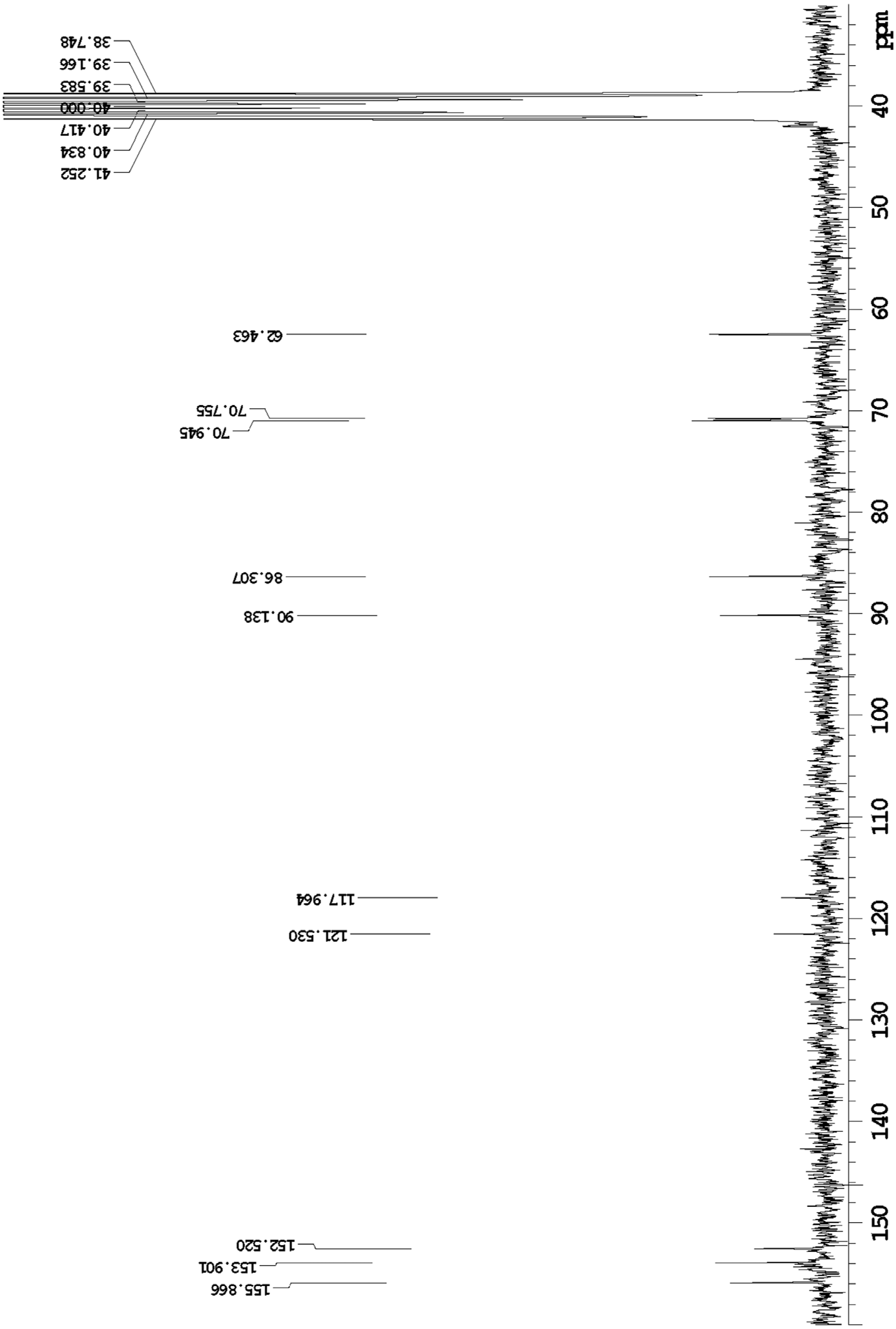

$^{13}C\{1H\}$NMR (dmso-d_6, 75 MHz) of **9**

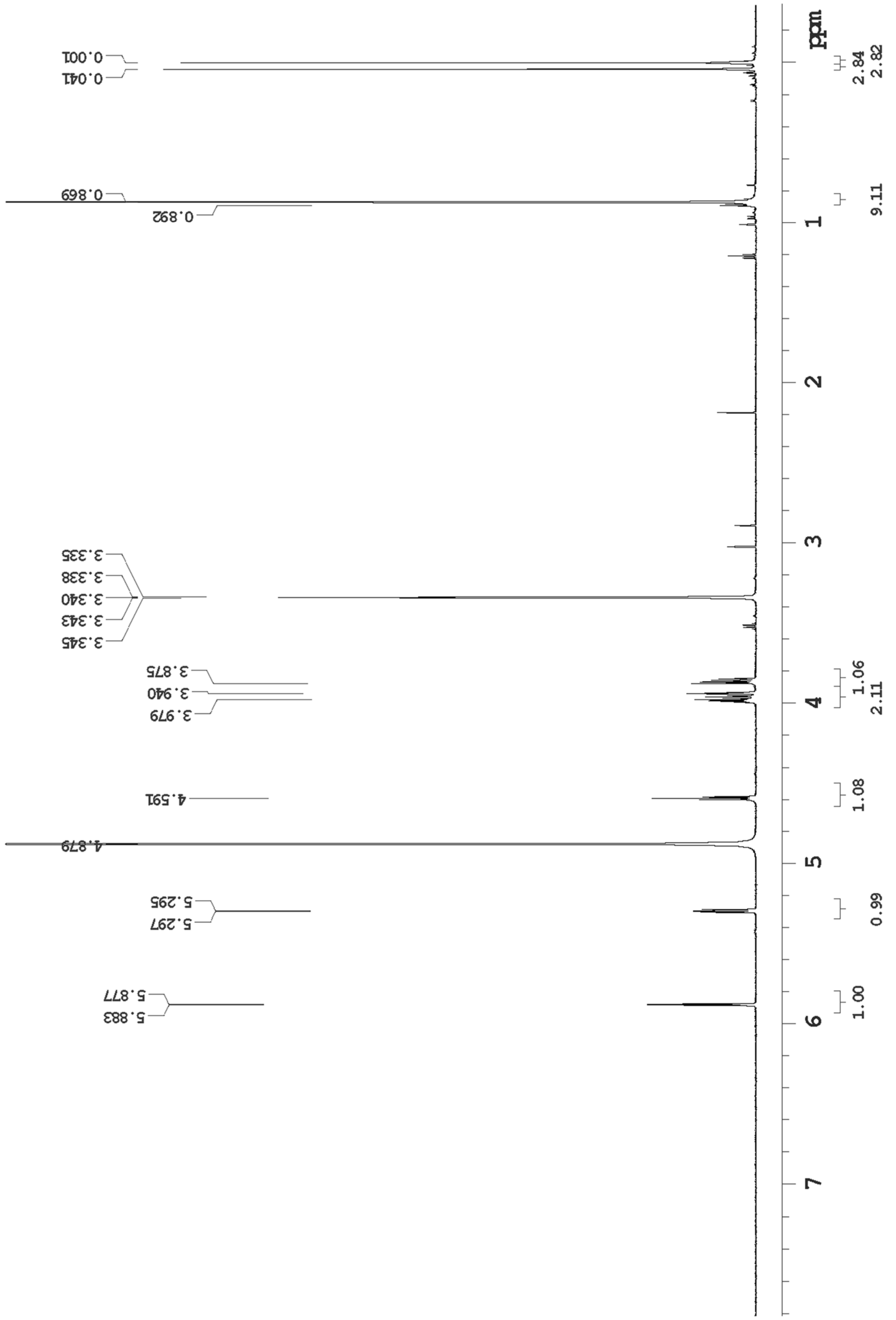

^{1}H-NMR (CD_3OD, 600 MHz) of **10**

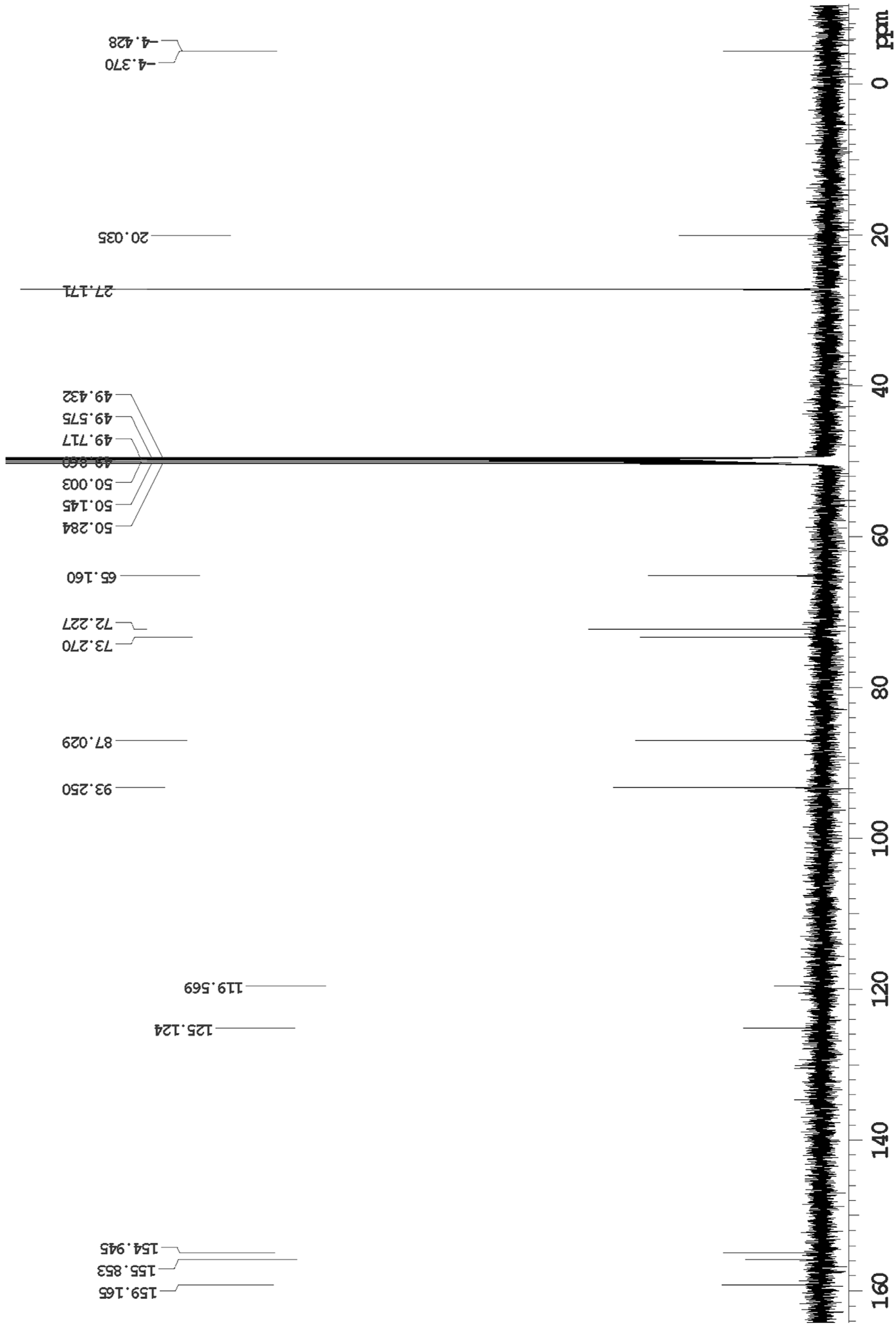

$^{13}C\{1H\}$NMR (CD_3OD, 151 MHz) of **10**

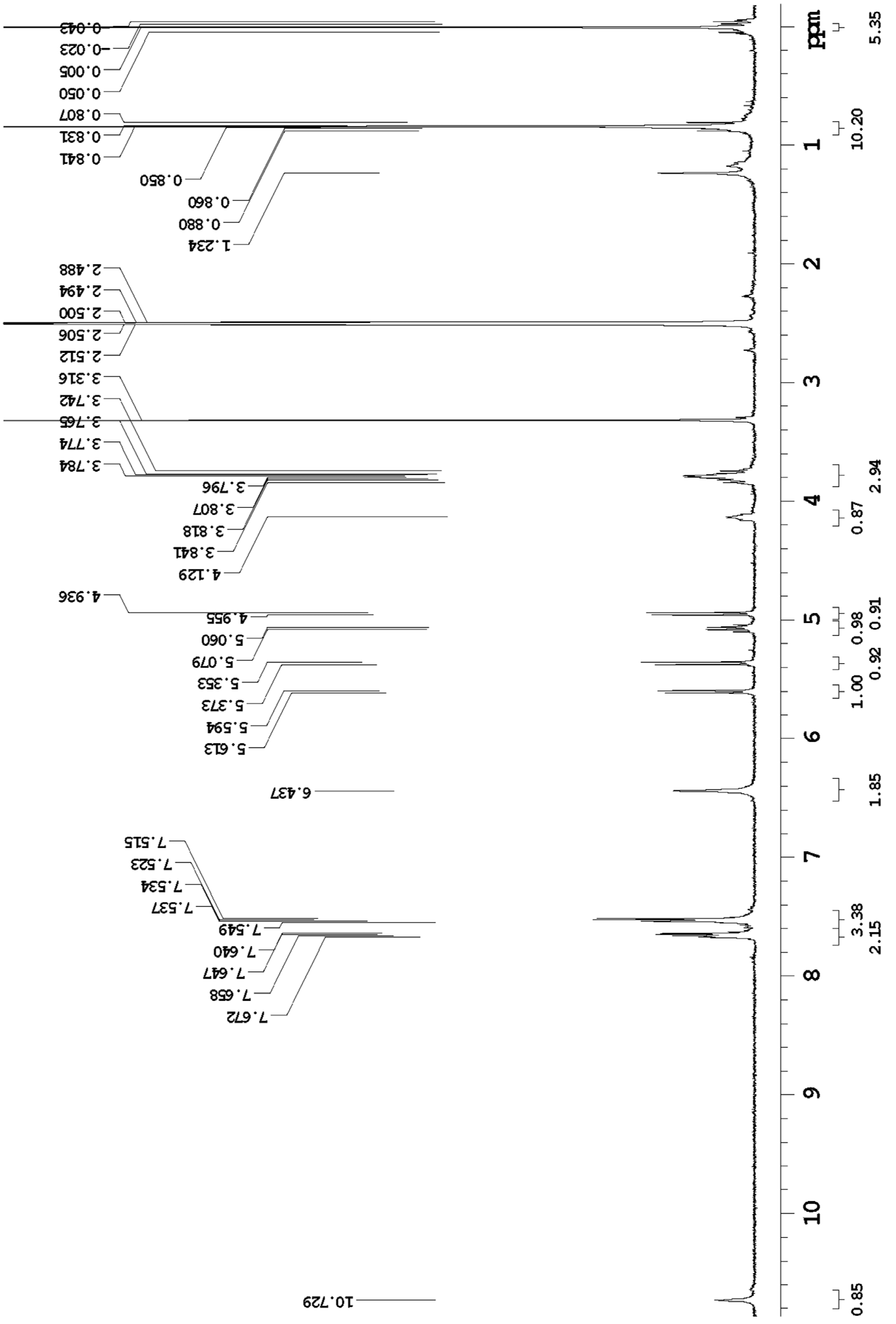

^{1}H-NMR (dmso-d_6, 600 MHz) of **11**

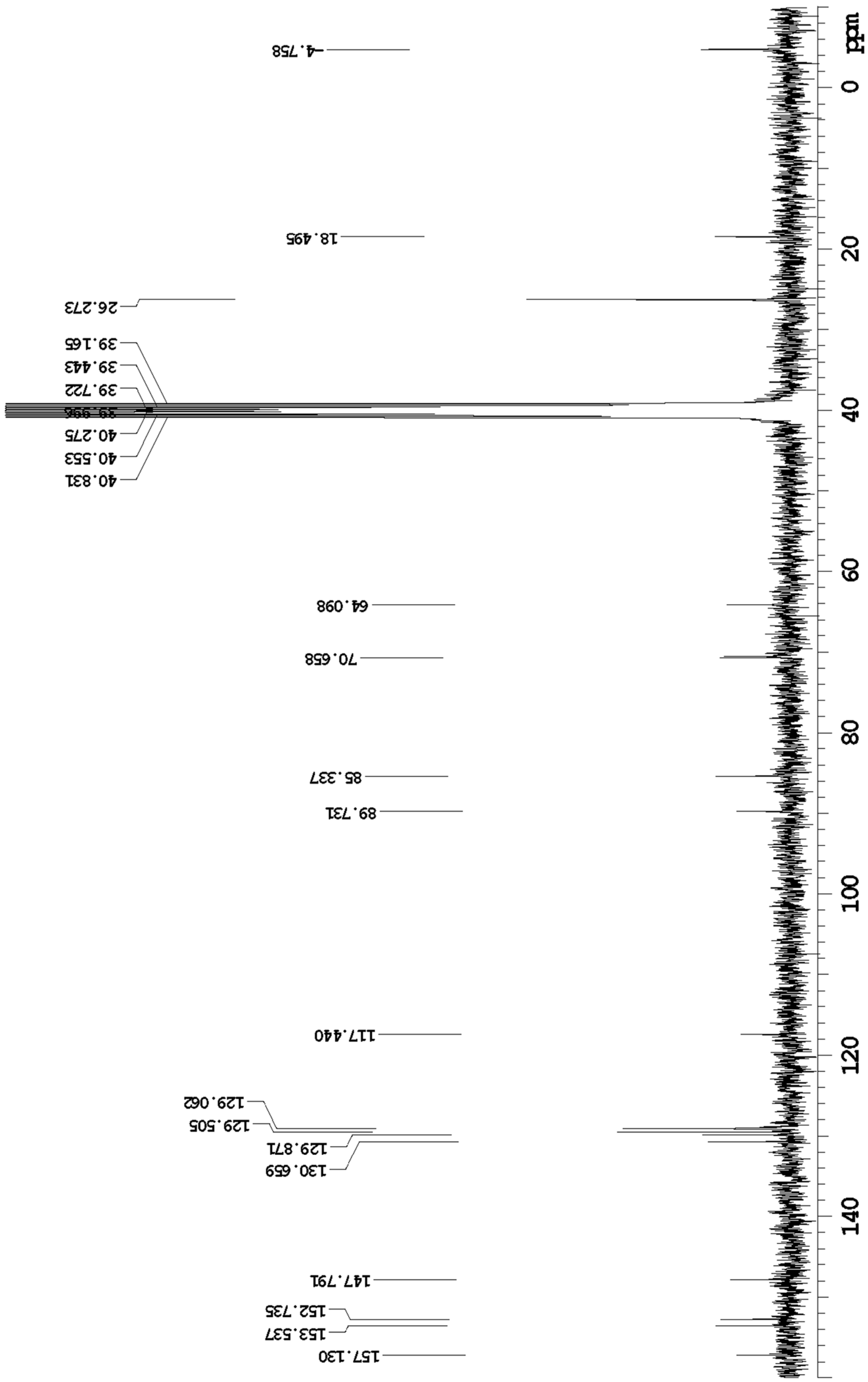

^{13}C{1H}NMR (dmso-d$_6$, 75 MHz) of **11**

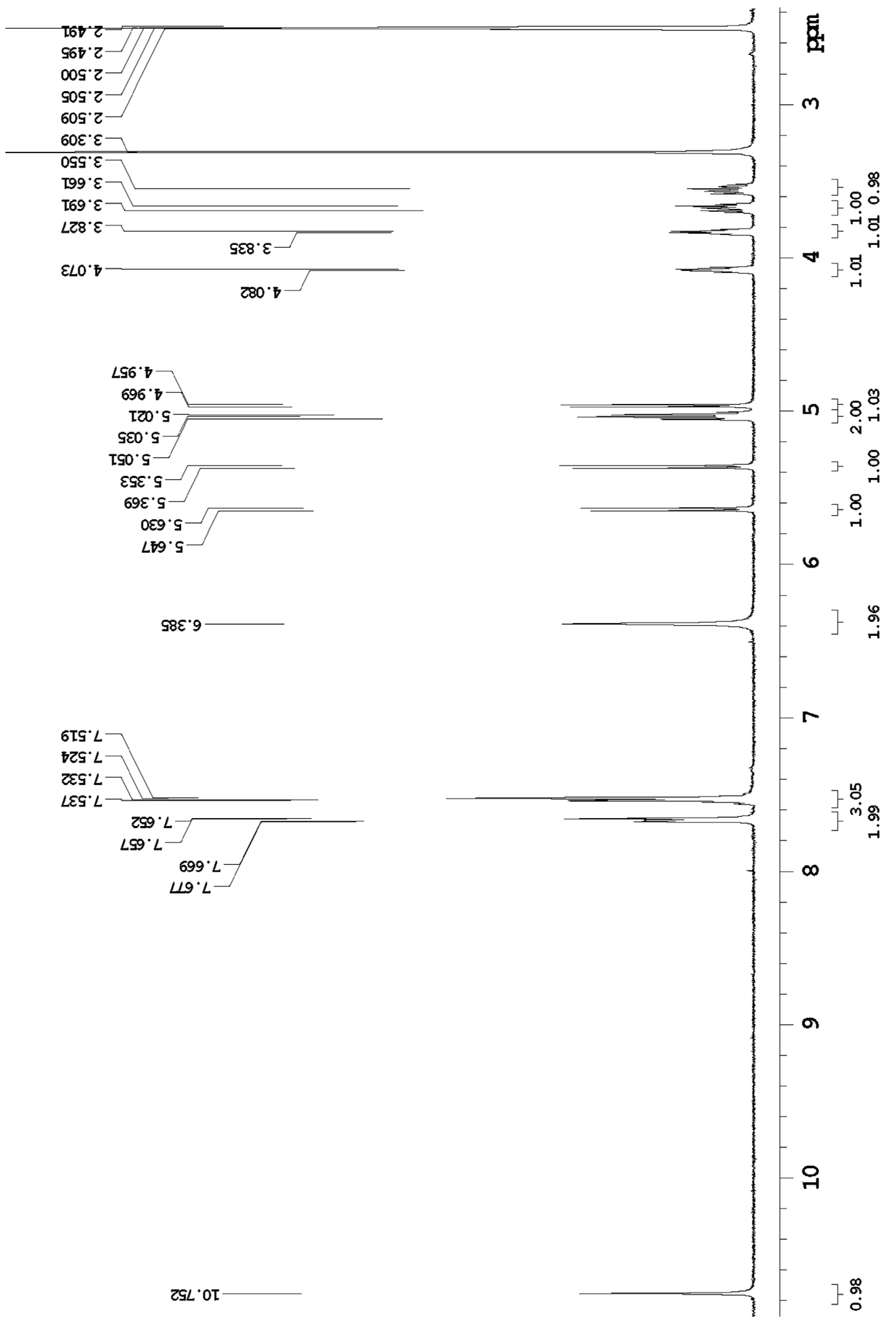

^{1}H-NMR (dmso-d$_6$, 400 MHz) of **12**

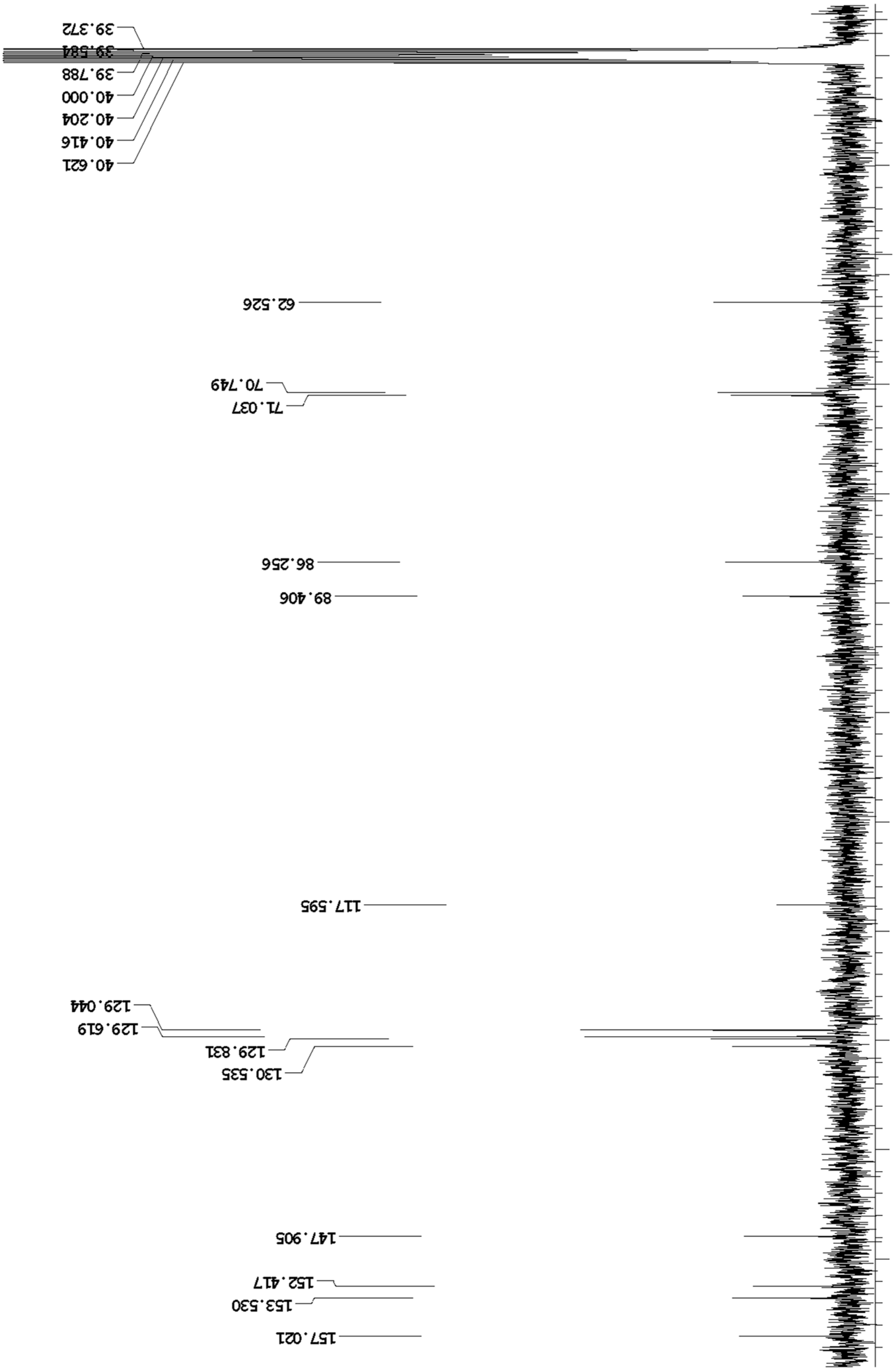

$^{13}C\{1H\}$NMR (dmso-d_6, 101 MHz) of **12**

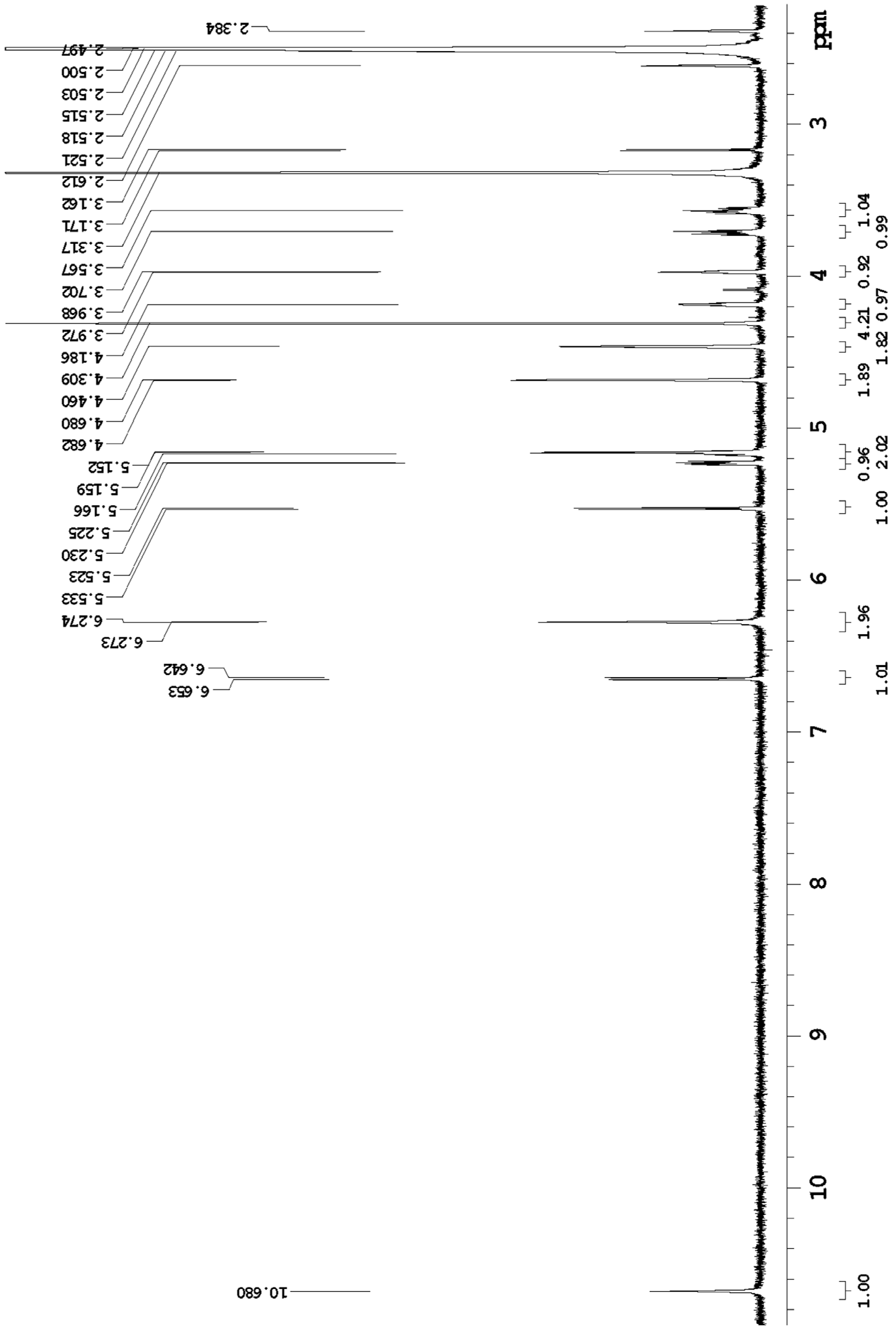

^{1}H-NMR (dmso-d$_6$, 600 MHz) of **13**

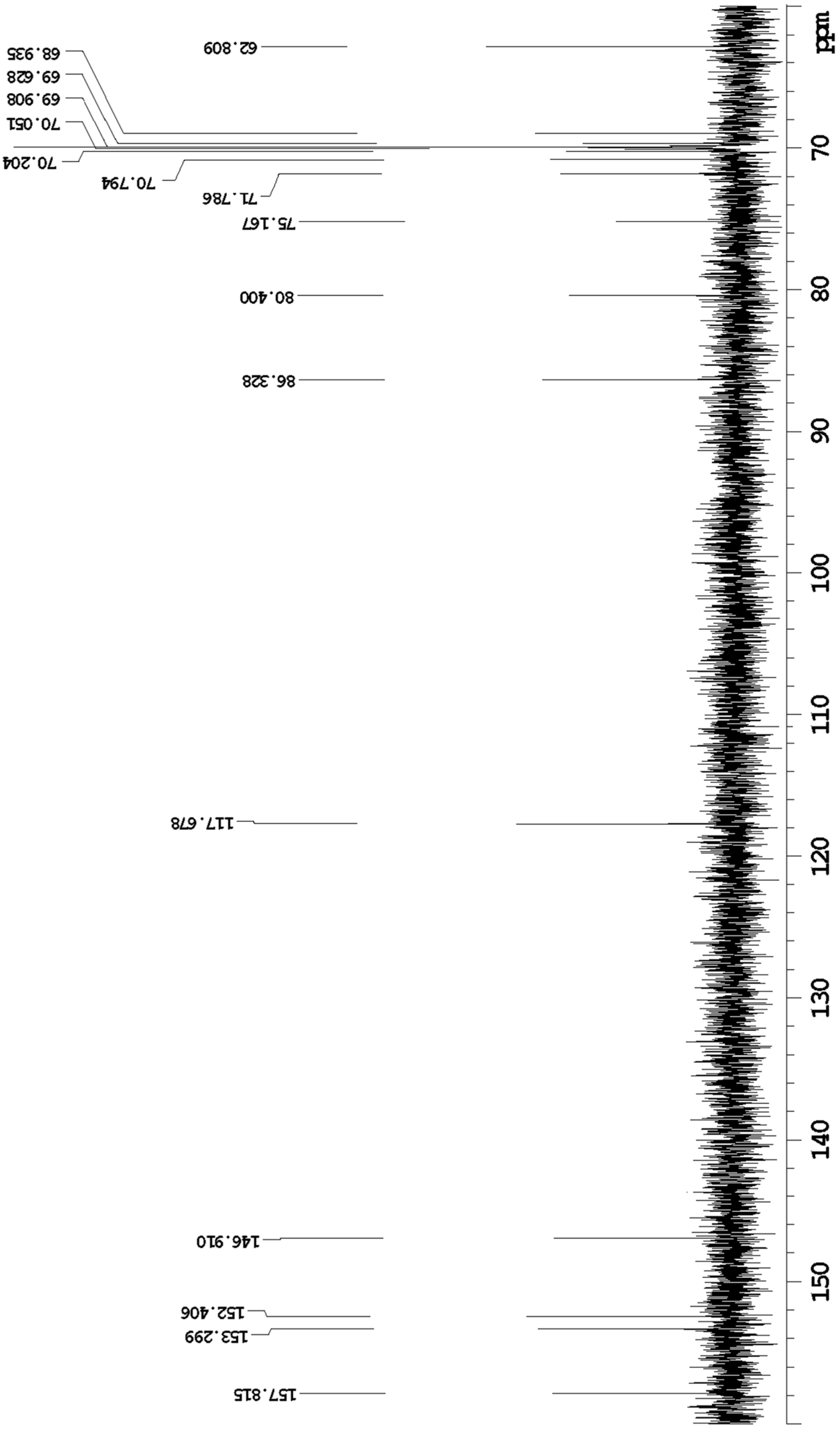

$^{13}C\{1H\}$NMR (dmso-d_6, 151 MHz) of **13**

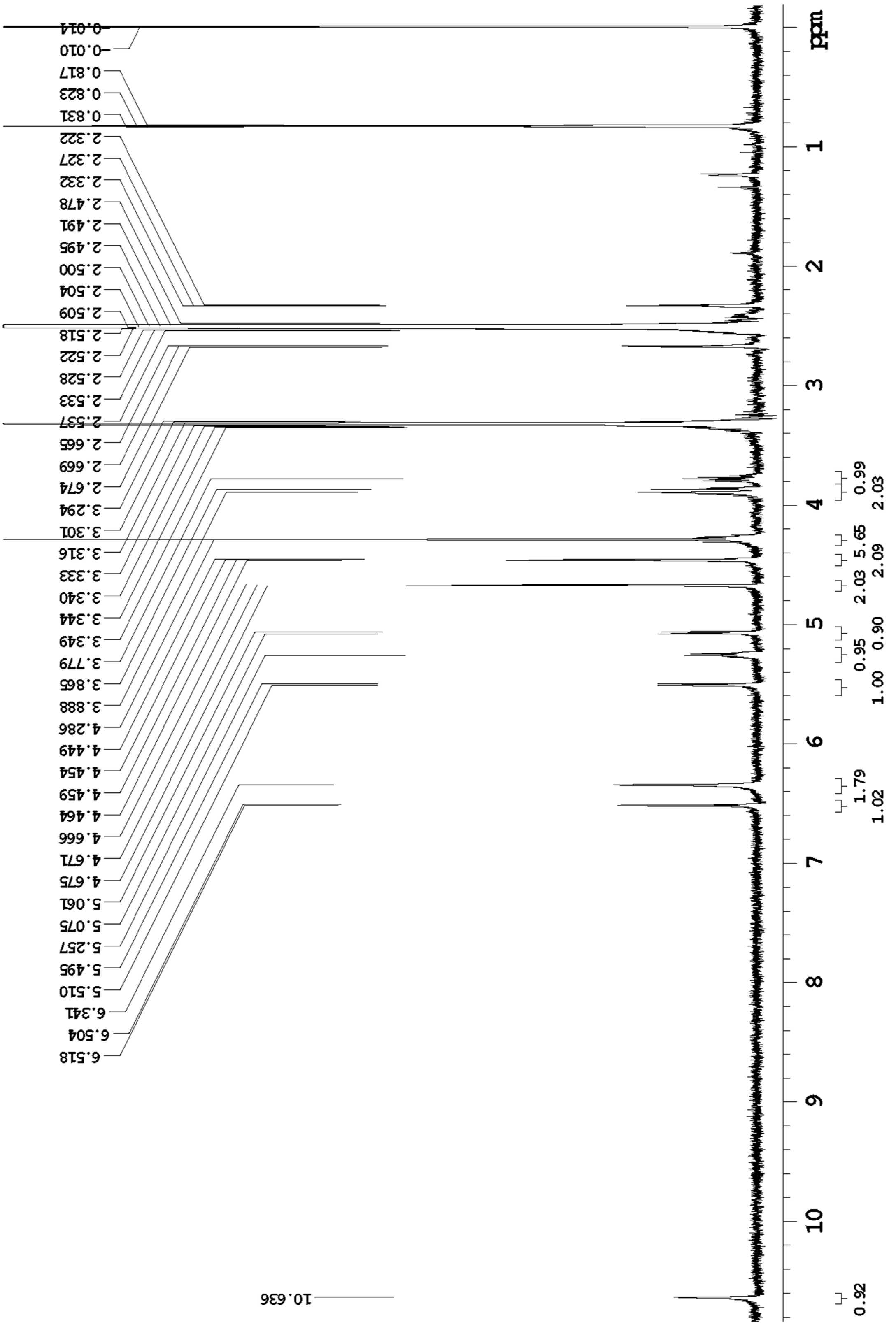

^{1}H-NMR (dmso-d$_6$, 400 MHz) of **14**

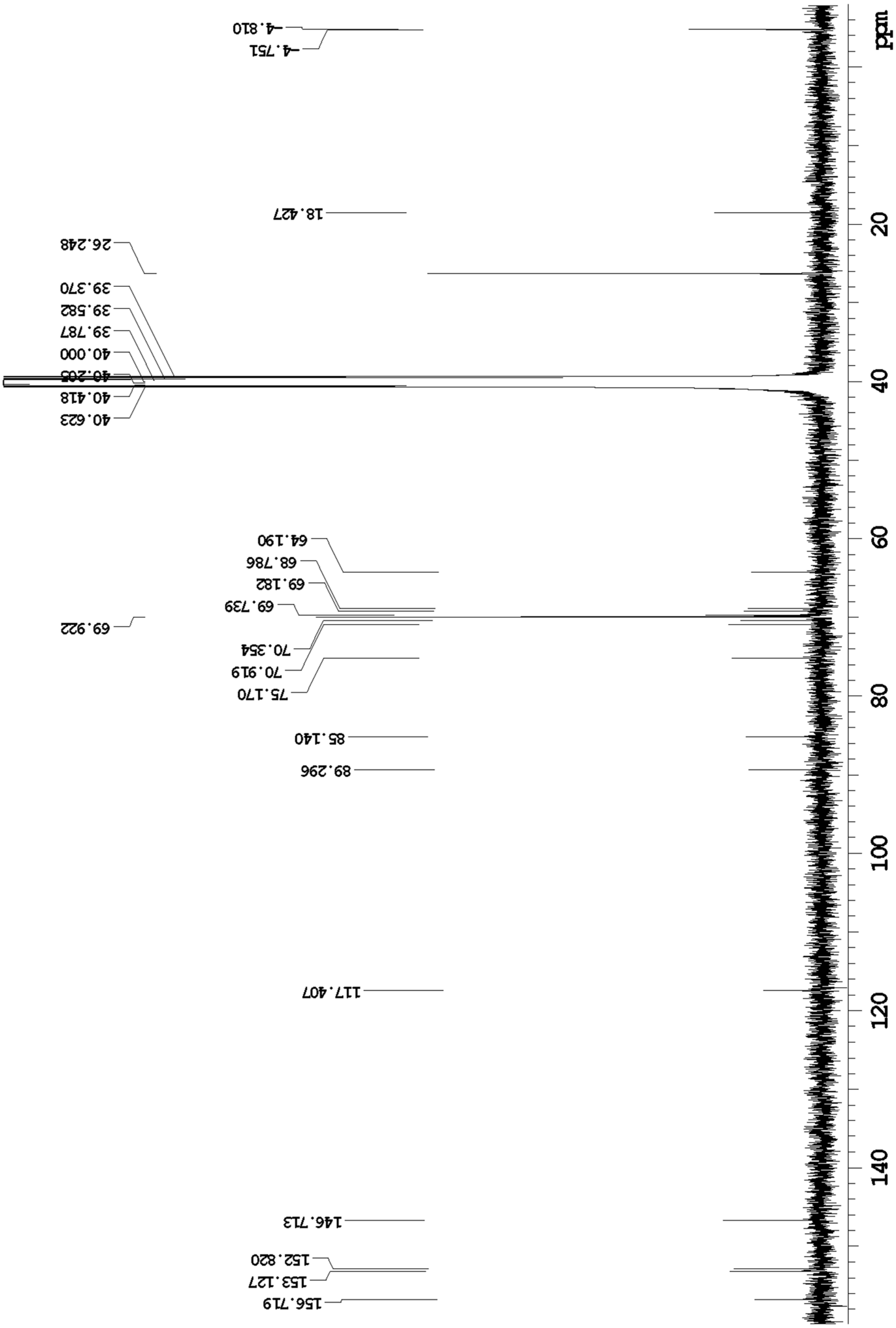

$^{13}C\{1H\}$NMR (dmso-d_6, 101 MHz) of **14**
